# Whole-Cell Transformation of Cannabidiol by Selected Filamentous Fungi into Novel Polar Derivatives

**DOI:** 10.3390/ijms27156884

**Published:** 2026-08-01

**Authors:** Daniel Łój, Tomasz Janeczko, Mariusz A. Bromke, Tomasz Tronina

**Affiliations:** 1 Department of Food Chemistry and Biocatalysis, Wrocław University of Environmental and Life Sciences, Norwida 25, 50-375 Wrocław, Poland; tomasz.janeczko@upwr.edu.pl; 2 Department of Biochemistry and Immunochemistry, Wroclaw Medical University, ul Chałubińskiego 10, 50-368 Wrocław, Poland; mariusz.bromke@umw.edu.pl

**Keywords:** cannabidiol, CBD, biotransformation, filamentous fungi, hydroxylation, glycosylation

## Abstract

Cannabidiol (CBD) is a bioactive phytocannabinoid with considerable pharmacological potential. However, its limited aqueous solubility and high lipophilicity remain significant barriers to its broader pharmaceutical application. In this study, the enzymatic potential of selected filamentous fungi was investigated as a whole-cell biocatalytic platform for the regioselective functionalization of CBD. Sixteen fungal strains were screened, and thirteen microorganisms successfully transformed CBD into more polar derivatives. Four strains showing distinct and promising chromatographic profiles were selected for scale-up biotransformation and product isolation: *Mucor hiemalis* KCh W2, *M. hiemalis* AM 450, *Isaria fumosorosea* KCh J2, and *Metarhizium robertsii* MU4. Eight CBD derivatives were isolated and identified by UHPLC-DAD, NMR spectroscopy, and HRESI-MS, including hydroxylated, glycosylated, and methylglycosylated products. Among them, two metabolites, 2′-O-(4‴-O-methyl-β-D-glucopyranosyl)-cannabidiol and 2′-O-(4‴-O-methyl-β-D-glucopyranosyl)-5″-hydroxycannabidiol, are reported here as previously undescribed CBD derivatives. *I. fumosorosea* KCh J2 and *M. robertsii* MU4 demonstrated the ability to catalyse 4-O-methylglycosylation. An additional experiment using 2′-O-(β-D-glucopyranosyl)-cannabidiol as an intermediate supported a sequential pathway involving initial phenolic O-glycosylation followed by methylation of the sugar moiety. In silico analysis predicted reduced lipophilicity for the newly obtained derivatives compared with CBD; however, these computational results require experimental verification and should not be interpreted as evidence of improved aqueous solubility, bioavailability, or biological activity. These findings demonstrate that filamentous fungi are useful whole-cell biocatalysts for generating structurally diverse CBD derivatives with increased polarity and provide new compounds for future physicochemical and biological evaluation.

## 1. Introduction

Cannabidiol (CBD) (**1**) is the primary non-psychoactive cannabinoid found in *Cannabis sativa* L. plants. Its biological activity is widely documented. These properties include immunomodulatory, anti-inflammatory, and antitumour activities, particularly against gliomas [1,2,3,4,5]. One of the principal pharmacological properties of cannabidiol (**1**) is its anticonvulsant activity [6]. In the United States, purified cannabidiol is approved by the FDA as Epidiolex for the treatment of seizures associated with Lennox–Gastaut syndrome, Dravet syndrome, and tuberous sclerosis complex in patients 1 year of age and older [7,8,9]. A major research challenge concerning cannabinoids, as well as other plant-derived compounds (with flavonoids serving as a prime example [10]), is their poor oral bioavailability, which is frequently attributed to their hydrophobic nature [11]. Enhancing their hydrophilicity may consequently improve their bioavailability as demonstrated in in vivo studies on the absorption of tetrahydrocannabinol (Δ^9^-THC), its hydroxylated derivative (11-hydroxy-Δ^9^-THC), and Δ^9^-THC glycoside from the gastrointestinal tract following oral administration [12]. One strategy to address this limitation involves the conjugation of polar groups, such as sugars and hydroxyl groups, to the cannabinoid molecule in order to increase its polarity and potentially modify its aqueous solubility, including solubility in physiological fluids [13,14]. It has been observed that for the aforementioned flavonoids, the attachment of sugar moieties to the core structure can result in a substantial increase in water solubility [15,16,17]. Furthermore, in addition to passively increasing hydrophilicity, the attachment of a sugar can facilitate active transport within the intestinal tract via SGLT1 and GLUT2 transporter proteins [18,19]. Through this active transport mechanism, the conjugated aglycone enters the bloodstream alongside the sugar moiety, potentially leading to enhanced systemic bioavailability [20]. Studies conducted in mice orally administered CBD-glucoside confirmed the presence of the intact compound in the small intestine, without its premature hydrolysis to free CBD [21]. This suggests that the compound may be absorbed directly, potentially utilizing active transport pathways such as SGLT1 and GLUT2, although this mechanism has not yet been definitively confirmed. It should also be emphasized that increased polarity does not necessarily imply improved pharmacological performance. CBD is a polypharmacological compound whose biological effects have been associated with multiple molecular targets and signalling pathways, including transient receptor potential channels, serotonin 5-HT1A receptors, GPR55, PPARγ, adenosine-related signalling, and modulation of the endocannabinoid system [22,23,24]. Therefore, structural modifications such as O-glycosylation of a phenolic hydroxyl group or additional hydroxylation may affect not only aqueous solubility and membrane permeability, but also target recognition, metabolic stability, cellular uptake, tissue distribution, and pharmacodynamics. In particular, masking a free phenolic group by glycosylation may alter interactions with biological targets. Consequently, the biological activity of glycosylated or hydroxylated CBD derivatives cannot be inferred directly from the activity of CBD itself and requires experimental verification. Beyond modifying physicochemical properties, structural modification of cannabidiol (**1**) may also alter its biological activity [25,26,27,28]. While methods for the chemical synthesis and enzymatic biosynthesis of glycosylated and hydroxylated cannabidiol derivatives are already documented [29,30], this study employs a whole-cell biotransformation approach. The aim of this study was to investigate the enzymatic potential of filamentous fungi for the biotransformation of cannabidiol (**1**) and to obtain and identify derivatives with increased polarity. The use of whole cells—specifically filamentous fungi, which have highly diverse enzymatic machinery resulted in the generation of previously undescribed derivatives and facilitated the synthetic process through the regioselective addition of functional groups.

## 2. Results and Discussion

**Figure 1:**
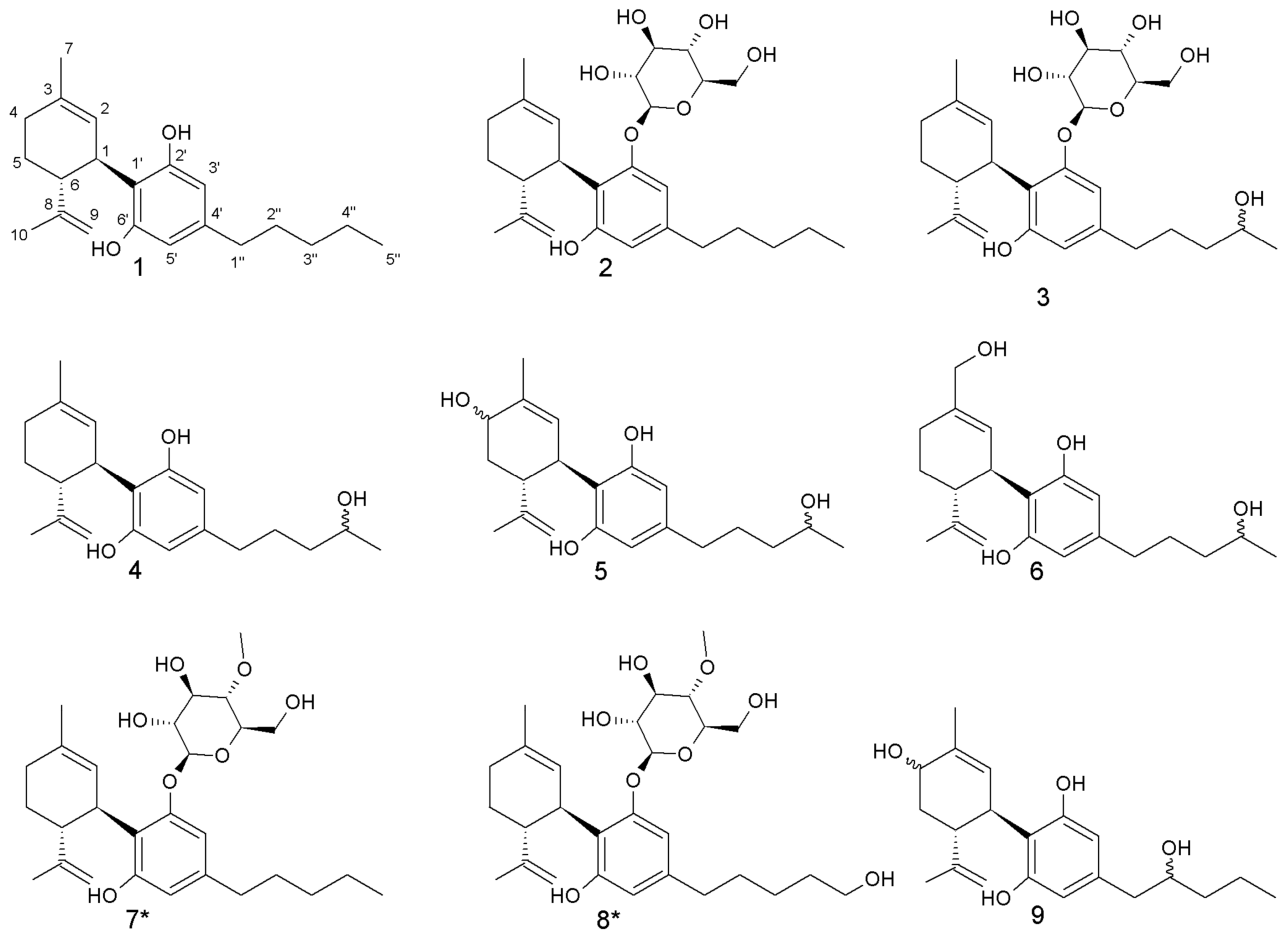
Structures of cannabidiol (**1**) and its isolated fungal biotransformation products (**2**–**9**). The starting material was natural (-)-trans-(3R,4R)-cannabidiol, and retained stereochemical elements of the CBD core are shown. Newly formed stereogenic centres in the aliphatic side chain for which the configuration could not be established are marked as configurationally undetermined. * Compounds **7** and **8** are previously undescribed derivatives.

Our previous studies have demonstrated that filamentous fungi, particularly entomopathogenic fungi belonging to the genera *Beauveria*, *Isaria*, and *Metarhizium*, are capable of the *O*-glycosylation and 4-*O*-methylglycosylation of plant-derived compounds, such as flavonoids [31,32,33]. This prompted the investigation into the biotransformation of cannabidiol (**1**), another bioactive plant-derived molecule containing a phenol group and terpene moiety. To expand the screening panel, strains from the genera *Mucor*, *Penicillium*, and *Absidia* were also included in the study. Based on the available literature, the expected outcome of using these specific strains was the formation of hydroxylated derivatives [34,35,36]. The application of the diverse enzymatic machinery of the investigated fungi resulted in the isolation and identification of eight cannabidiol derivatives (**2**–**9**), two of which, to the best of our knowledge, were novel and previously undescribed metabolites **7** and **8** (Figure 1).

The starting material was natural (-)-*trans*-(*3R*,*4R*)-cannabidiol, and the stereochemical configuration of the CBD core was retained in the assigned metabolite structures. The configuration of the newly formed stereogenic centre could not be determined and is indicated as configurationally undetermined.

### 2.1. Screening Studies

Selected strains of filamentous fungi were investigated to assess their biotransformation capabilities. Due to the lack of authentic standards for all metabolites and potential variations in UV response factors and extraction efficiencies among CBD, its hydroxylated products, and glycosides, UHPLC-DAD peak-area data are reported only as semi-quantitative chromatographic estimates. The values should be interpreted as apparent substrate depletion and the relative chromatographic composition estimated by UHPLC-DAD analysis based on mean of peak areas (±standard deviation) of the ethyl acetate extracts, rather than as absolute conversion, product yield, or mass balance. The obtained results demonstrate the capacity of the investigated fungal strains to biotransform CBD.

The apparent substrate depletion determined via UHPLC analysis is illustrated in Table 1. Among the tested strains, thirteen showed detectable transformation of cannabidiol (**1**), whereas three strains showed no detectable activity under the applied conditions. Among the active strains, entomopathogenic fungi of the genera *Metarhizium* and *Isaria* showed pronounced CBD depletion and diverse metabolite profiles, indicating that their enzymatic repertoire is suitable for the transformation of plant-derived compounds. Initially, three strains were selected for further studies: *Metarhizium robertsii* MU4, *Isaria fumosorosea* KCh J2, and *Mucor hiemalis* KCh W2. The results obtained for another strain of the *M. hiemalis* species, AM 450, also supported its selection for scale-up because of its distinct metabolite profile and satisfactory substrate depletion, and provided an opportunity to compare enzymatic capabilities within a single species. Ultimately, four strains were selected for scaled-up biotransformation to isolate and characterize the metabolites—the transformation products of cannabidiol (**1**).

The selection of strains for scale-up biotransformation was not based solely on the apparent CBD depletion values shown in Table 1. Because these values are semi-quantitative and do not represent absolute conversion or product yield, the decision also considered the chromatographic profile of the extracts, reproducibility between biological replicates, presence of assigned or potentially new CBD-derived metabolites, biomass growth, abundance of endogenous fungal metabolites and pigments, and practical feasibility of product isolation. Thus, *M. hiemalis* KCh W2 and *M. hiemalis* AM 450 were selected because they showed pronounced CBD depletion, good biomass growth, relatively clean extract profiles, and accumulation of glycosylated and hydroxylated derivatives that could be isolated efficiently. *I. fumosorosea* KCh J2 was selected because its chromatographic profile indicated the formation of methylglycosylated metabolites, including the previously undescribed compounds **7** and **8**. *M. robertsii* MU4 was selected despite the presence of abundant endogenous metabolites and pigments because it showed a distinct profile involving methylglycosylation and hydroxylation, leading to compounds **7** and **9**. Other active strains, including *I. farinosa* KCh KW 1.1 and *I. tenuipes* MU35, showed detectable CBD transformation but were not prioritized for scale-up because their profiles were less favourable for isolation under the applied conditions and/or showed higher variability or lower selectivity toward target metabolites.

**Figure 2:**
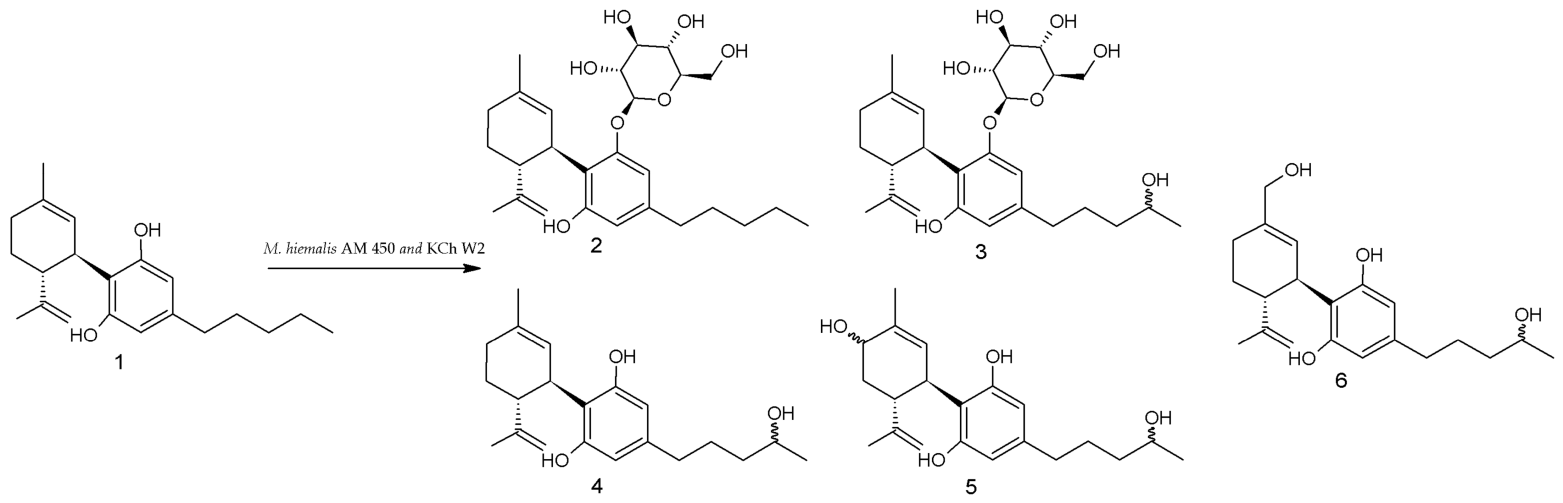
Biotransformation of CBD (**1**) by *M. hiemalis* strains AM 450 and KCh W2.

It is noteworthy that *M. hiemalis* KCh W2 strain showed the greatest relative depletion of the CBD chromatographic peak among the tested strains. This strain produced a dominant glycosylated derivative under the applied conditions. Strains *M. hiemalis* AM 450 and KCh W2 in addition to producing the major glycosylated derivative also showed the ability to perform regioselective hydroxylation. A total of five biotransformation products were isolated and identified: glycoside: 2′-*O*-(β-D-glucopyranosyl)-cannabidiol (**2**); hydroxylated derivatives: 4″-hydroxycannabidiol (**4**), 4,4″-dihydroxycannabidiol (**5**), and 7,4″-dihydroxycannabidiol (**6**); hydroxylated and glycosylated derivative: 2′-*O*-(β-D-glucopyranosyl)-4″-hydroxycannabidiol (**3**) (Figure 2).

In addition to pronounced CBD peak depletion, both *M. hiemalis* strains exhibited a broad spectrum of enzymatic capabilities. A further advantage of utilizing these microorganisms is their substantial biomass growth, as well as the simplicity of the extraction and purification steps. Unlike the other evaluated microorganisms, these strains produced minimal amounts of pigments and endogenous metabolites, which significantly facilitated the isolation and purification of the reaction products.

**Figure 3:**
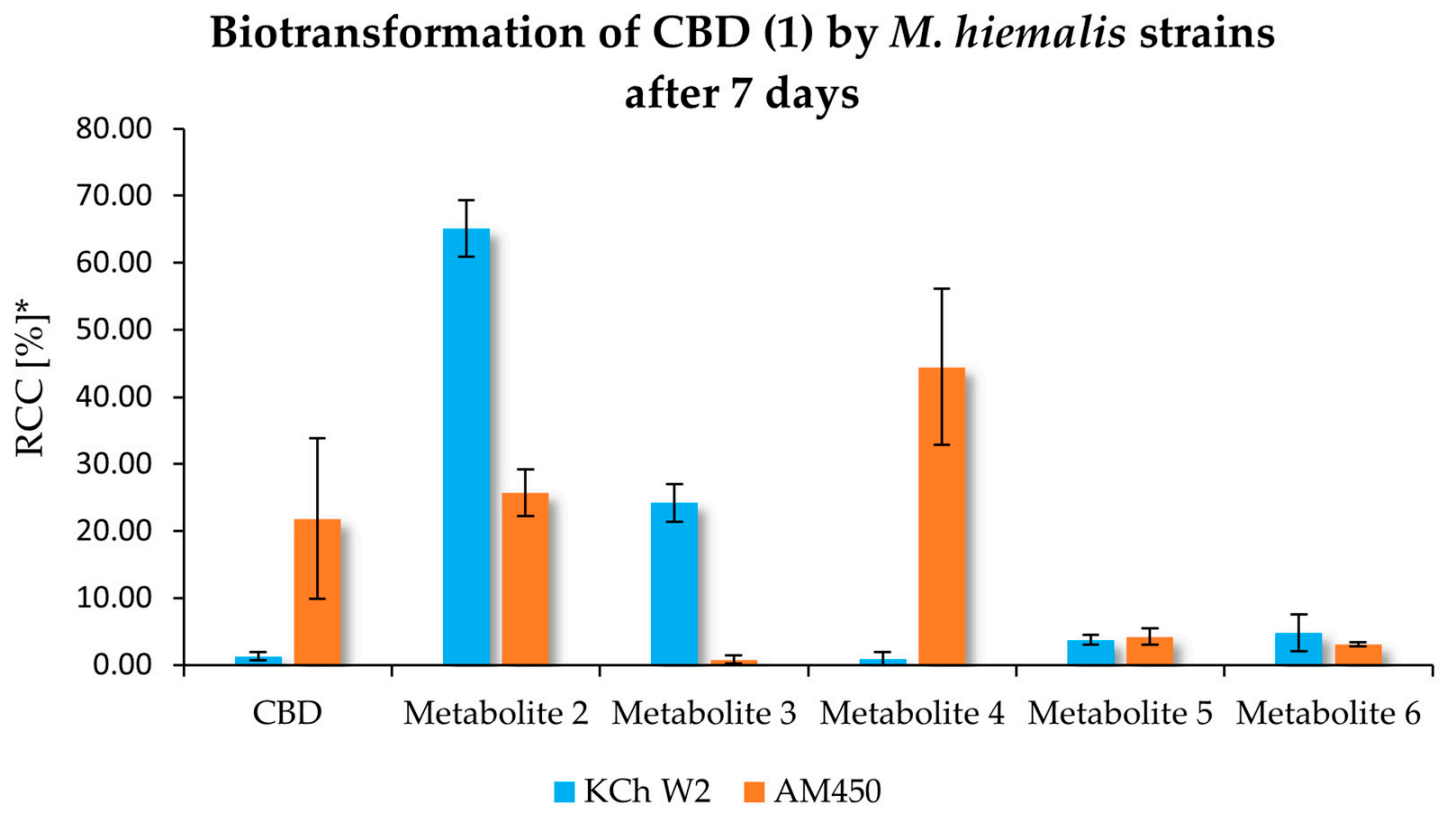
Biotransformation of CBD (**1**) by *M. hiemalis* strains after 7 days; * Data expressed as mean of Relative Chromatographic Composition (RCC) ± SD from three independent biological replicates according to the UHPLC-DAD analyses.

Figure 3 shows that, under the applied conditions, *M. hiemalis* KCh W2 caused a significantly greater relative depletion of the CBD (**1**) chromatographic peak than *M. hiemalis* AM 450 (*p* = 0.003). Only the day-7 profile is shown for the *M. hiemalis* strains because this time point was used for comparison of the final metabolite distribution and for selecting conditions for product isolation. In addition, the relative UHPLC-DAD peak areas of metabolites **2** and **3** were significantly higher in the chromatographic profile obtained for *M. hiemalis* KCh W2 (*p* = 0.002 and *p* = 0.024, respectively). Conversely, strain *M. hiemalis* AM 450 favoured the formation of metabolite **4**, exhibiting a significantly higher relative UHPLC-DAD peak-area contribution for this specific derivative (*p* = 0.014). No statistically significant differences between the investigated strains were observed regarding the production of metabolites **5** and **6** (*p* > 0.05) (Supplementary Table S5). In summary, *M. hiemalis* KCh W2 showed a more pronounced apparent CBD-depletion profile under the applied conditions for cannabidiol (**1**) biotransformation; the resulting metabolic profiles demonstrate considerable intra-species variability. By contrast, another strain of the same species, *M. hiemalis* AM 729, exhibited no catalytic activity. This suggests that the capacity for CBD (**1**) biotransformation is a strain-dependent trait rather than a characteristic common to the entire species.

**Figure 4:**
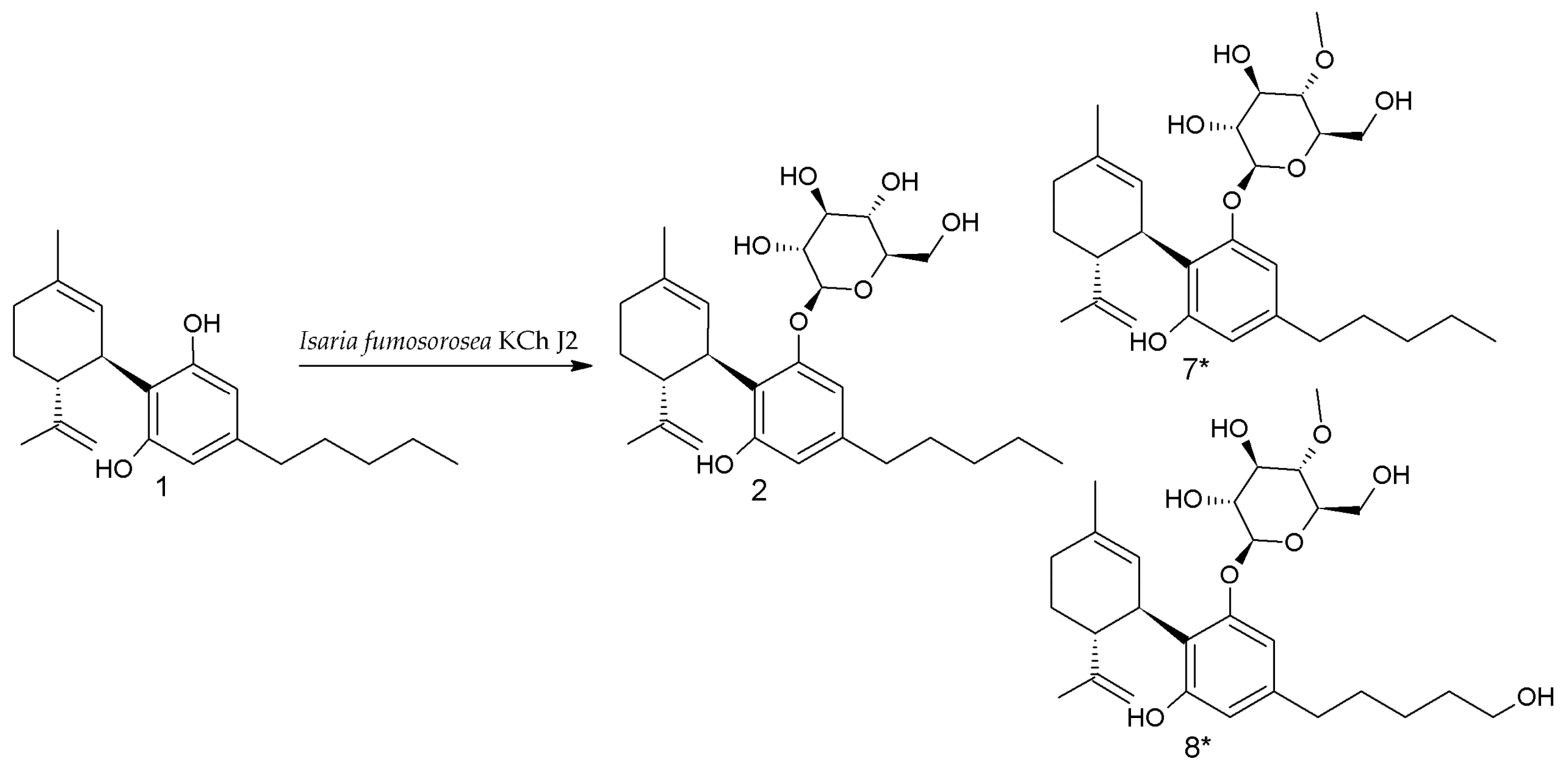
Biotransformation of CBD (**1**) by *Isaria fumosorosea* KCh J2 * Previously undescribed compounds.

To the best of our knowledge, two previously undescribed cannabidiol derivatives, **7** and **8**, were obtained during these studies. The two previously undescribed metabolites identified as 2′-O-(4‴-O-methyl-β-D-glucopyranosyl)-cannabidiol (**7**) and 2′-O-(4‴-O-methyl-β-D-glucopyranosyl)-5″-hydroxycannabidiol (**8**) were obtained in the culture of *Isaria fumosorosea* KCh J2 (Figure 4). Additionally, metabolite **8** underwent hydroxylation, which further contributes to the increased hydrophilicity of the derivative, thereby fulfilling the primary objective of the study.

**Figure 5:**
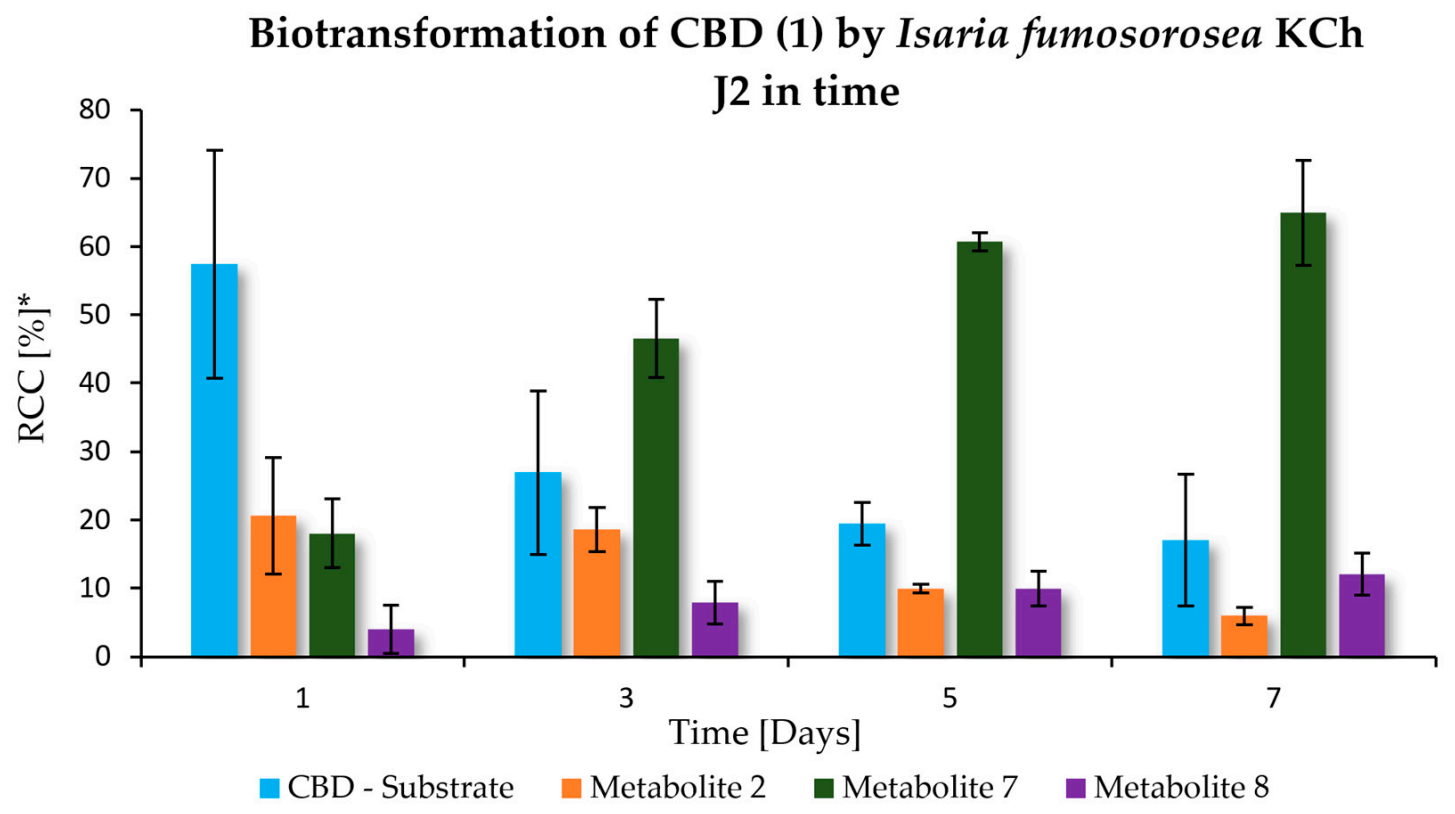
Biotransformation of CBD (**1**) by *Isaria fumosorosea* KCh J2 in time; * Data expressed as mean of Relative Chromatographic Composition (RCC) ± SD from three independent biological replicates according to the UHPLC-DAD analyses.

**Figure 6:**
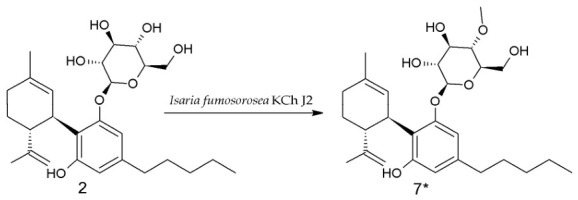
Biotransformation of metabolite **2** to metabolite **7** via *I. fumosorosea* KCh J2; * Previously undescribed compound.

Notably, metabolite **2** was formed as early as the first day; its concentration decreased over time (Figure 5); statistical analysis revealed a significant difference between its concentration on day 1 and day 7 (*p* < 0.05) (Supplementary Table S2). Conversely, for metabolite 7, a significant increase in production was observed over the course of the process (*p* < 0.05) (Supplementary Table S4). This trend suggests that metabolite **2** serves as an intermediate, indicating that the enzymatic methylglycosylation process does not occur through a single synergistic mechanism but rather proceeds through sequential stages. Furthermore, although an upward trend for metabolite **8** is visible on the graph, statistical analysis showed no significant difference between its production levels on day 1 and day 7 (Supplementary Table S3). To test the intermediate-compound hypothesis, a short experiment was conducted in which small amounts of 2′-*O*-(β-D-glucopyranosyl)-cannabidiol (**2**) (obtained from processes using *Mucor* strains) were added to a pre-grown culture of *Isaria fumosorosea* KCh J2. After two days, samples were collected for UHPLC analysis. The results strongly support that the first step of cannabidiol (**1**) metabolism by the tested strain is the glycosylation of the phenolic position, followed by methylation of the added sugar moiety at the C-4‴ position (Figure 6). The conversion of metabolite **2** into metabolite **7** after 2 days was 90.66 ± 4.52%.

The sequential mechanism involving the initial glycosylation of phenolic hydroxyl groups followed by the methylation of the C4-OH position on the glucose moiety by entomopathogenic fungi aligns well with reported literature data [37,38]. Sun et al. demonstrated that the formation of methylglycosides by insect-pathogenic fungi is responsible for detoxifying phenolic compounds accumulated by herbivores [39]. Plant defense compounds possessing antimicrobial properties can enhance herbivore immunity against pathogens, including fungal infections. In turn, methylglycosylation catalyzed by entomopathogenic fungi serves as a vital detoxification pathway for phenolic substrates such as flavonoids and stilbenes. This process operates via a two-step mechanism consisting of the glycosylation of phenolic hydroxyl groups mediated by UDP-dependent glycosyltransferases (GTs), followed by the methylation of the C4 hydroxyl group of the sugar moiety catalyzed by methyltransferases (MTs), which renders the resulting compounds non-toxic to fungi and resistant to hydrolysis by β-glucosidases [39]. This pathway is encoded by functional GT-MT (glycosyltransferase–methyltransferase) gene pairs [37,38]. Although most investigated fungi within the order *Hypocreales* harbor these gene pairs to form a functional methylglucosylation module, their catalytic efficiency and substrate specificity vary depending on the strain and cultivation conditions [37]. For example, the whole-cell biotransformation of kaempferol by *B. bassiana* and *M. robertsii* yields methylglucosides exclusively, whereas cultures of *I. fumosorosea* produce both glucosides and methylglucosides. These discrepancies are suggested to arise from several factors, including: variations in methyltransferase (MT) gene expression levels that prevent immediate methylation of the newly formed glucosides; in situ deglycosylation by endogenous fungal glycosidases that hydrolyze glycoside intermediates back to aglycones prior to methylation; and differences in cellular substrate permeability that restrict the availability of intermediate products to intracellular enzymes [37].

**Figure 7:**
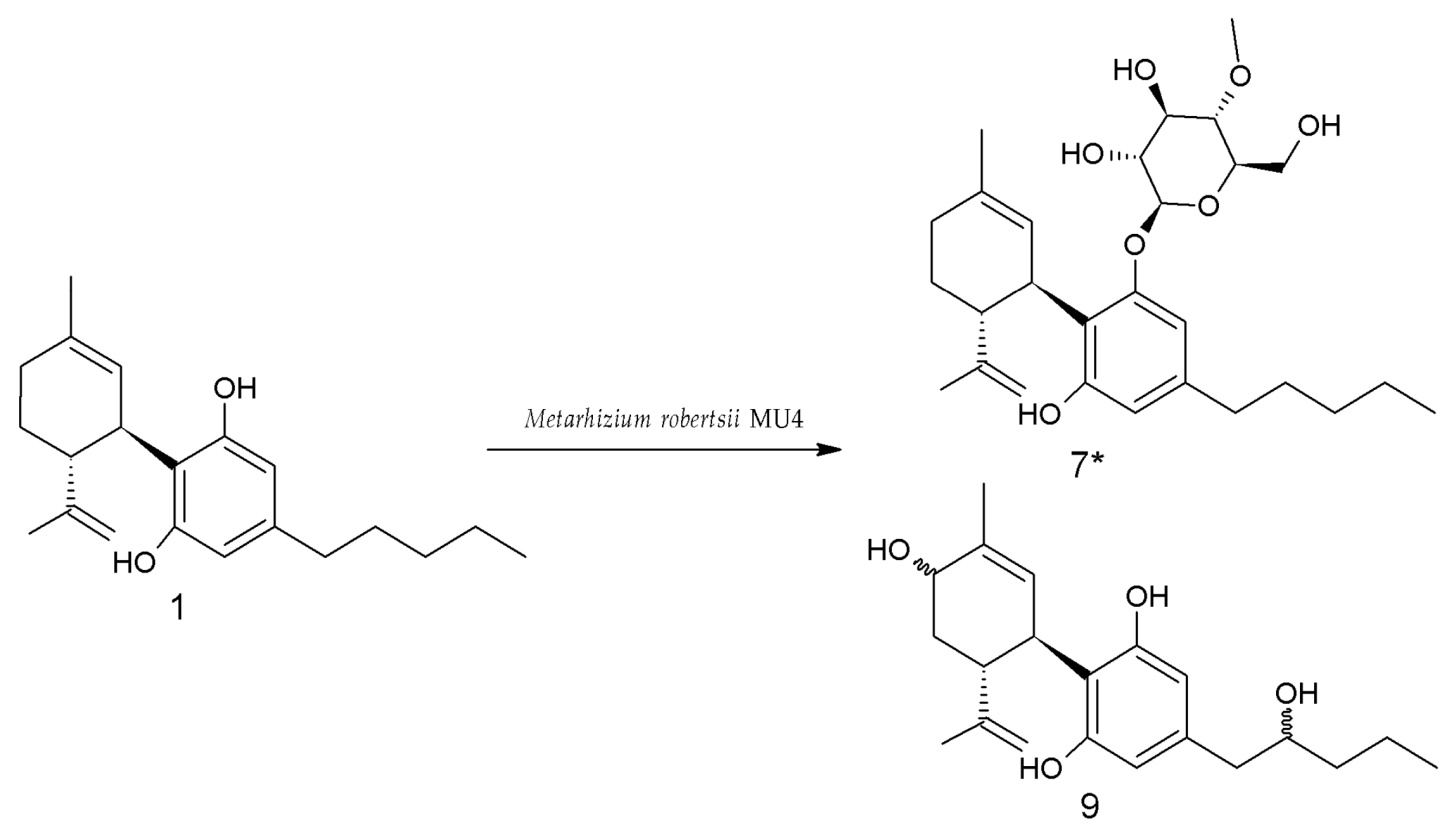
Biotransformation of CBD **1** by *M. robertsii* MU4; * Previously undescribed compound.

**Figure 8:**
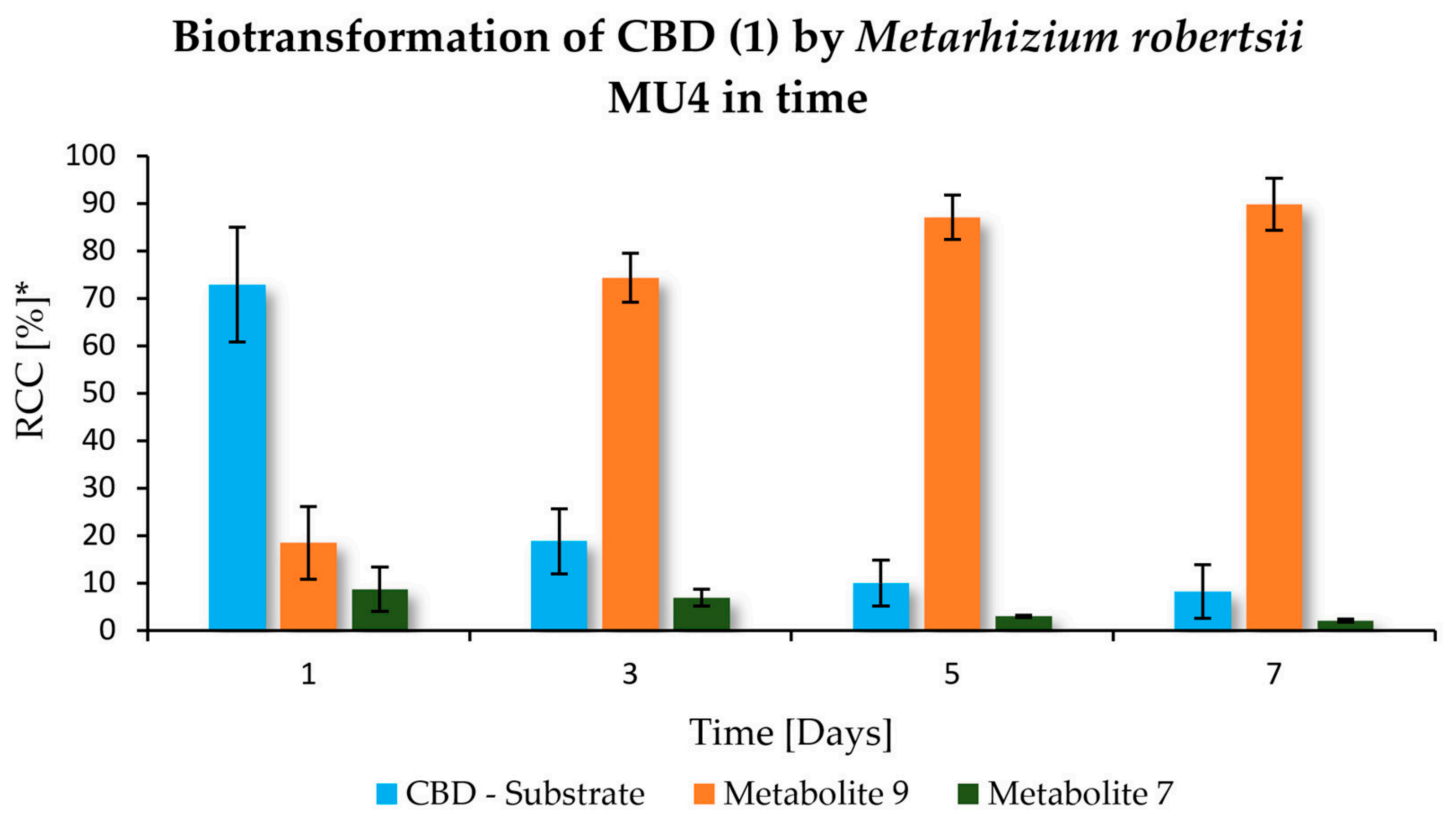
Biotransformation of CBD (**1**) by *Metarhizium robertsii* MU4 in time; * Data expressed as mean of Relative Chromatographic Composition (RCC) ± SD from three independent biological replicates according to the UHPLC-DAD analyses.

The final strain selected for the isolation and identification of biotransformation products was *Metarhizium robertsii* MU4. Like the other fungal strains investigated, this microorganism produced a characteristic profile of endogenous metabolites; however, in the case of *M. robertsii* MU4, the abundance of these components and pigments significantly complicated product isolation and purification. Comparison with the corresponding fungal control culture, together with analysis of the DAD UV absorption profiles, enabled CBD-derived chromatographic peaks to be distinguished from the endogenous fungal background. Despite these difficulties, two derivatives were successfully identified among the isolated compounds: the aforementioned novel, previously undescribed 2′-*O*-(4‴-*O*-methyl-β-D-glucopyranosyl)-cannabidiol (**7**) and 4,2″-dihydroxycannabidiol (**9**) (Figure 7). This finding corroborates the previously described capability of numerous entomopathogenic fungi to perform 4-*O*-methylglycosylation of plant-derived compounds such as flavonoids [31,33,40]; cannabidiol proved to be no exception to this established pattern. The biotransformation of CBD by *M. robertsii* MU4 in time is presented in Figure 8.

As shown in Figure 8, the relative contribution of metabolite **7** decreased during the *M. robertsii* MU4 time course, whereas metabolite **9** became the dominant detected product. This trend may reflect further metabolism of compound **7**, differences in extraction efficiency, compound-dependent partitioning, or analytical variability in the semi-quantitative UHPLC-DAD profiles. Because no complete mass-balance analysis of the aqueous phase and fungal biomass was performed, this decrease should be interpreted cautiously and cannot be assigned unambiguously to a specific transformation pathway.

The primary objective of this study was successfully achieved, resulting in the generation of more polar derivatives of cannabidiol **1**. In addition to the two entirely previously undescribed compounds (**7** and **8**) other products (**2**–**6** and **9**) were also obtained. Consequently, this study demonstrates the significant advantages of highly regioselective enzymatic reactions over the complexities of traditional synthetic chemistry, aligning with the principles of green chemistry. It should be emphasized that the isolated yields reported below refer to purified compounds obtained after scale-up biotransformation, extraction, fractionation, and chromatographic purification. These values should not be directly compared with the semi-quantitative UHPLC-DAD peak-area data obtained during screening. In the present study, the purification strategy was focused primarily on obtaining compounds of sufficient purity for reliable structural elucidation by NMR spectroscopy and HRESI-MS, rather than on optimizing isolated yield. Therefore, the relatively low isolated yields of some metabolites may reflect, at least in part, losses during multistep purification and the prioritization of compound purity over recovery. The isolated yields of the purified metabolites and the spectroscopic data of novel compounds **7** and **8** are as follows:

**Compound 7:** Isolated as a pale yellow amorphous solid; 34.4 mg (22.4%, UHPLC purity 99.3%) (*Isaria fumosorosea* KCh J2) and 6.9 mg (1.1%, UHPLC purity 98,0%) (*Metarhizium robertsii* MU4). Spectral data (Supplementary Figures S55–S64):

^1^H NMR (800 MHz, DMSO-d_6_) δ [ppm]: 6.36 (d, *J* = 1.6 Hz, 1H, H-3′), 6.23 (br s, 1H, H-5′), 5.12 (brs, 1H, H-2), 4.71 (d, *J* = 7.8 Hz, 1H, H-1‴), 4.53 (d, *J* = 2.9 Hz, 1H, H-9a), 4.38 (brs, 1H, H-9b), 3.97 (brd, *J* = 8.7 Hz, 1H, H-1), 3.63 (dd, *J* = 9.8, 5.0 Hz, 1H, H-6‴a), 3.53–3.48 (m, 1H, H-6‴b), 3.46 (s, 3H, 4‴-OCH_3_), 3.43–3.38 (m, 1H, H-3‴), 3.33–3.27 (m, 1H, H-5‴), 3.26–3.21 (m, 1H, H-2‴), 3.15–3.08 (m, 1H, H-6), 3.03 (t, *J* = 9.3 Hz, 1H, H-4‴), 2.41–2.34 (m, 2H, H-1″), 2.16–2.09 (m, 1H, H-4a), 1.96–1.91 (m, 1H, H-4b), 1.71–1.67 (m, 1H, H-5a), 1.63 (td, *J* = 12.4, 5.4 Hz, 1H, H-5b), 1.61 (brs, 3H, H-7), 1.58 (s, 3H, H-10), 1.51 (p, *J* = 7.7 Hz, 2H, H-2″), 1.33–1.23 (m, 4H, H-3″, H-4″ [overlapping signals]), 0.87 (t, *J* = 7.2 Hz, 3H, H-5″).

^13^C NMR (200 MHz, DMSO-d_6_) δ [ppm]: 156.9 (C-2′), 156.4 (C-6′), 149.3 (C-8), 141.1 (C-4′), 130.7 (C-3), 127.4 (C-2), 117.2 (C-1′), 110.6 (C-9), 109.9 (C-5′), 106.8 (C-3′), 101.5 (C-1‴), 79.7 (C-4‴), 76.8 (C-2‴), 76.2 (C-3‴), 70.4 (C-5‴), 60.8 (C-6‴), 60.1 (4‴-OCH_3_), 44.5 (C-6), 36.1 (C-1), 35.6 (C-1″), 31.5 (C-3″), 30.8 (C-4), 30.6 (C-2″), 29.8 (C-5), 23.7 (C-7), 22.4 (C-4″), 19.8 (C-10), 14.4 (C-5″). HRESI-MS: [M + H]+; *m*/*z* 491.3008 (calcd. 491.3003; mass error = −1.02 ppm). UV (MeOH) λ_max_: 207.1, 274.5 nm.

**Compound 8:** Isolated as a pale yellow amorphous solid; 2.8 mg (1.7%, UHPLC purity 97.4%) (*Isaria fumosorosea* KCh J2). Spectral data (Supplementary Figures S65–S73):

^1^H NMR (800 MHz, DMSO-d_6_) δ [ppm]: 6.36 (s, 1H, H-5′), 6.23 (brs, 1H, H-3′), 5.12 (s, 1H, H-2), 4.71 (d, *J* = 7.7 Hz, 1H, H-1‴), 4.53 (d, *J* = 2.7 Hz, 1H, H-9a), 4.38 (brs, 1H, H-9b), 3.97 (d, *J* = 10.6 Hz, 1H, H-1), 3.63 (d, *J* = 11.8 Hz, 1H, H-6‴a), 3.50 (dd, *J* = 12.0, 5.0 Hz, 1H, H-6‴b), 3.46 (s, 3H, 4‴-OCH_3_), 3.42 (t, *J* = 8.9 Hz, 1H, H-3‴), 3.39 (t, *J* = 6.6 Hz, 2H, H-5″), 3.36–3.20 (m, 2H, H-2‴ and H-5‴ [overlapping signals]), 3.13–3.06 (m, 1H, 6-H), 3.04 (t, *J* = 9.3 Hz, 1H, H-4‴), 2.41–2.36 (m, 2H, H-1″), 2.16–2.10 (m, 1H, H-4a), 1.96–1.91 (m, 1H, H-4b), 1.70–1.66 (m, 1H, H-5a), 1.64 (dt, *J* = 12.3, 5.4 Hz, 1H, H-5b), 1.61 (s, 3H, H-7), 1.58 (s, 3H, H-10), 1.51 (p, *J* = 7.7 Hz, 2H, H-2″), 1.43 (dt, *J* = 14.4, 6.7 Hz, 2H, H-4″), 1.29 (tt, *J* = 9.5, 6.1 Hz, 2H, H-3″).

^13^C NMR (201 MHz, DMSO-d_6_) δ [ppm]: 157.0 (C-2′), 156.3 (C-6′), 149.4 (C-8), 141.1 (C-4′), 131.2 (C-3), 127.4 (C-2), 117.3 (C-1′), 110.4 (C-9), 110.3 (C-5′), 106.9 (C-3′), 101.3 (C-1‴), 79.8 (C-4‴), 76.9 (C-3‴), 76.2 (C-5‴), 74.5 (C-2‴), 61.2 (C-5″), 60.9 (C-6‴), 60.0 (4‴-OCH_3_), 44.1 (C-6), 36.2 (C-1), 35.7 (C-1″), 32.9 (C-4″), 30.8 (C-2″ and C-4 [overlapping signals]), 29.8 (C-5), 25.8 (C-3″), 23.7 (C-7), 19.9 (C-10). HRESI-MS: [M + H]+; *m*/*z* 507.2956 (calcd. 507.2952; mass error = −0.79 ppm). UV (MeOH) λ_max_: 209.7, 274.1 nm.

The reported values should be interpreted as isolated yields of purified metabolites obtained after scale-up biotransformation, extraction, fractionation, and chromatographic purification. These values should not be interpreted as total product formation or directly compared with the semi-quantitative UHPLC-DAD peak-area data obtained during screening. Standard extraction methods were employed, and the purification conditions were established empirically. However, the optimization of downstream processing was beyond the scope of this study. Given that the remaining compounds, with the exception of the to the best of our knowledge, previously undescribed structures **7** and **8**, are already known and have been previously characterized [30], further investigations were focused exclusively on the newly discovered metabolites.

### 2.2. Structural Identification of 2′-O-(4‴-O-Methyl-β-D-glucopyranosyl)-cannabidiol (***7***)

For the preliminary identification of substrate derivatives, i.e., cannabidiol (**1**), the UV spectra (λ_max_= 210 and 275 nm, Supplementary Figure S2) recorded during UHPLC analysis were compared. The analysis of samples collected from the biotransformation using the *I. fumosorosea* KCh J2 strain yielded unequivocal results. The appearance of a new peak with a highly similar UV absorption spectrum confirmed that the product belongs to the cannabinoid class (Supplementary Figure S56). Under the applied reversed-phase UHPLC conditions, compound 7 exhibited a shorter retention time than CBD (Rt = 3.02 versus 3.60 min), which is consistent with increased chromatographic polarity (Supplementary Figures S1 and S51). Following the isolation and purification of compound **7** to a purity level sufficient for a comprehensive NMR analysis the following structural features were elucidated: the NMR spectra of the obtained metabolite **7** differed from those of the substrate **1** primarily by the appearance of seven additional carbon signals in the ^13^C NMR spectrum in the range of δ 60–105 ppm (Supplementary Figure S60). These carbons correlated with proton signals in the δ 2.90–5.10 ppm region in the ^1^H NMR spectrum, which was confirmed by the HSQC correlation NMR (Supplementary Figure S62). The presence of these signals in the NMR spectra corroborates the attachment of a sugar moiety. Furthermore, in addition to the six aforementioned carbon signals, a characteristic singlet at a chemical shift of δ 3.46 ppm, integrating to three protons (3H), was observed in the ^1^H NMR spectrum (Supplementary Figure S58). These spectral features confirm the presence of a methoxy (-OCH_3_) group. The acquired spectral data confirm that the attached sugar moiety is 4-*O*-methyl-glucopyranose. To assign the carbon signals to their respective proton signals and to determine the chemical shifts for the specific carbon atoms within the sugar ring, an HSQC NMR correlation spectrum was recorded (Supplementary Figure S62). The structure of compound **7** was further supported by detailed 2D NMR analysis. HSQC correlations allowed the assignment of the glucopyranosyl carbon–proton pairs, including the anomeric signal H-1‴/C-1‴ at δH 4.71 ppm/δC 101.5 ppm and the oxygenated sugar carbons in the δC 60.8–79.7 ppm range (Supplementary Figure S62). The anomeric proton appeared as a doublet with *J* = 7.8 Hz, confirming the β-configuration of the glucopyranosyl unit. The COSY spectrum (Supplementary Figure S61) showed the expected sequential correlations within the sugar spin system, H-1‴/H-2‴, H-2‴/H-3‴, H-3‴/H-4‴, H-4‴/H-5‴, and H-5‴/H-6‴, confirming the glucopyranose skeleton. The presence of a methoxyl group was confirmed by the singlet at δH 3.46 ppm, integrating for three protons, and by the corresponding HSQC correlation with the carbon signal at δC 60.1 ppm (Supplementary Figure S62). Its location at C-4‴ of the sugar moiety was supported by the HMBC correlation from the methoxyl group protons to C-4‴ (δC 79.7 ppm) (Supplementary Figure S63). The attachment of the sugar moiety to the phenolic oxygen at C-2′ of cannabidiol was confirmed by the diagnostic HMBC correlation from the anomeric proton H-1‴ to C-2′ (Supplementary Figure S63). Taken together, these data established compound **7** as 2′-O-(4‴-O-methyl-β-D-glucopyranosyl)cannabidiol. A structure and keyword-based search of chemical and scientific literature databases did not reveal any previous reports of compounds identical to compound **7**. Therefore, to the best of our knowledge, 2′-O-(4‴-O-methyl-β-D-glucopyranosyl)cannabidiol (**7**) has not been previously described.

### 2.3. Structural Identification of 2′-O-(4‴-O-Methyl-β-D-glucopyranosyl)-5″-hydroxycannabidiol (***8***)

As with the previous compound **7**, the primary diagnostic criterion was the similarity of the UV spectra of the metabolites to that of the substrate (**1**). In addition to the previously described metabolite **7**, the chromatogram obtained from the biotransformation using the *I. fumosorosea* KCh J2 strain revealed the presence of a second peak exhibiting a UV spectrum closely resembling that of substrate **1** (Supplementary Figures S2 and S66). This biotransformation product displayed a significantly shorter retention time (Rt = 2.4 min vs. 3.6 min) using C-18 UHPLC analysis (Supplementary Figures S1 and S65). Under the applied reversed-phase UHPLC conditions, compound **8** exhibited a shorter retention time than both CBD and compound **7** (Rt = 2.40, 3.60, and 3.02 min, respectively), which is consistent with increased chromatographic polarity (Supplementary Figures S55 and S65). Performed ^1^H NMR analysis of compound **8** demonstrated similarity to the spectrum of metabolite **7**, with a minor structural difference localized within the aliphatic chain region (C1″–C5″). Specifically, the characteristic triplet corresponding to the terminal methyl group (-CH_3_) observed at δ 0.87 ppm in ^1^H NMR spectrum of compound **7** (Supplementary Figure S58) was absent in ^1^H NMR spectrum of compound **8** (Supplementary Figure S68). Based on the 2D NMR COSY correlation spectra, the missing signal was successfully located (Supplementary Figure S71). The protons of the terminal group resonate at δ 3.39 ppm in the ^1^H NMR spectrum, while their corresponding carbon atom appears at δ 61.2 ppm, as determined by the HSQC NMR analysis (Supplementary Figure S72). Such chemical shifts indicate the attachment of a hydroxyl group, resulting in the formation of a primary alcohol. Furthermore, the COSY spectrum revealed a cross-peak correlating the protons at δ 3.39 ppm with a broad singlet at δ 4.26 ppm (Supplementary Figure S71). This observation confirms the presence of a hydroxyl group at the terminal position (C-5″) of the aliphatic chain. The assignment of compound **8** was further supported by comparison with the 2D NMR data of compound **7**. HSQC correlations confirmed the presence of the anomeric signal H-1‴/C-1‴ at δH 4.71 ppm/δC 101.3 ppm, the methoxy group at δH 3.46 ppm/δC 60.0 ppm, and the hydroxymethyl group assigned to C-5″ at approximately δH 3.38–3.39 ppm/δC 61.0–61.2 ppm (Supplementary Figure S72). The COSY spectrum showed sequential correlations within the glucopyranosyl unit, as well as the aliphatic-chain correlation H-4″/H-5″, supporting the terminal position of the hydroxylated methylene group (Supplementary Figure S71). The HMBC correlation from the methoxy protons to C-4‴ confirmed methylation of the glucopyranosyl unit at C-4‴, while the diagnostic HMBC correlation from H-1‴ to C-2′ supported glycosylation at the phenolic group of cannabidiol. In addition, HMBC correlations from H-5″ to C-4″ and C-3″ further supported the terminal hydroxylation at C-5″ (Supplementary Figure S73). Therefore, compound **8** was identified as 2′-O-(4‴-O-methyl-β-D-glucopyranosyl)-5″-hydroxycannabidiol. The same structure- and keyword-based literature and database search did not reveal any previous reports of compounds identical to compound **8**. Therefore, to the best of our knowledge, 2′-O-(4‴-O-methyl-β-D-glucopyranosyl)-5″-hydroxycannabidiol (**8**) has not been previously described [40]. In addition, comparison with previously reported chemically synthesized and enzymatically obtained CBD derivatives did not reveal any reports of compounds **7** and **8**, supporting their assignment as previously undescribed cannabidiol derivatives.

### 2.4. In Silico Studies

**Figure 9:**
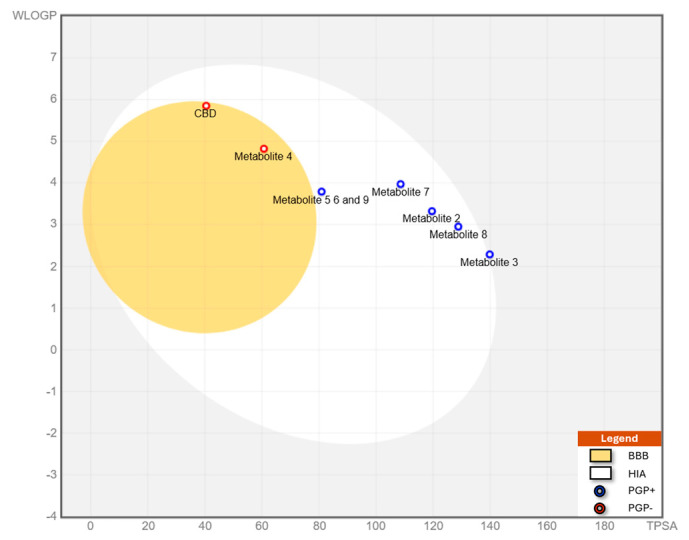
The BOILED-Egg predictive model. The model illustrating the potential human intestinal absorption and brain penetration of the evaluated metabolites. The plot was generated using the SwissADME web tool [43] BBB: blood–brain barrier; HIA: human intestinal absorption; PGP-(red dots): molecules not subjected to active efflux by P-glycoprotein; PGP+ (blue dots): molecules actively effluxed by P-glycoprotein.

To investigate the predicted physicochemical properties and ADME profiles of the novel compounds **7** and **8**, a series of bioinformatic analyses was conducted, starting with pharmacokinetic ADME screening using the SwissADME tool [41]. The objective was to compare the results of in silico studies of obtained biotransformation products with those of the substrate, cannabidiol **1**. It should be emphasized that SwissADME, BOILED-Egg, and PASS Online are predictive tools based on computational models and training datasets. Their outputs should therefore be interpreted as preliminary estimates that depend on model assumptions and applicability domains, rather than as experimentally validated physicochemical, pharmacokinetic, or biological properties. The derivative **7** is characterized by a lower predicted lipophilicity coefficient (Log P_o/w_ = 3.76 compared to CBD (**1**) Log P_o/w_ = 4.3). Unlike CBD (**1**), product **7** is predicted not to passively penetrate the blood–brain barrier (BBB). The obtained product meets the druglikeness criteria according to the Lipinski, Veber, Egan, and Muegge rules. Its bioavailability was calculated as 0.55, which aligns with the value obtained for the substrate **1**. It is noteworthy that while CBD **1** violates Muegge rules, product **7** fulfills them. The lower predicted LogP value of compound **7** compared with CBD indicates reduced predicted lipophilicity and is consistent with the introduction of a polar carbohydrate moiety. However, this result should not be interpreted as experimental evidence of increased aqueous solubility [42]. A similar study was applied to compound **8**. The ADME analysis revealed a significantly lower lipophilicity coefficient (Log P_o/w_= 2.49) compared to both the substrate **1** and product **7**. This lower predicted LogP value is consistent with the presence of the additional hydroxyl group and increased molecular polarity. Nevertheless, the actual aqueous solubility of compounds 7 and 8 remains to be determined experimentally. The hydroxylation is predicted not to significantly alter the molecule’s ability to cross the blood–brain barrier (BBB); however, the attachment of the sugar moiety is expected to effectively hinder passive permeation across this barrier due to a substantial increase in molecular mass and size. Metabolite **8** is predicted to comply with the drug-likeness rules of Lipinski, Egan, and Muegge. SwissADME assigned the same model-based bioavailability score of 0.55 to CBD (**1**) and compounds **7** and **8**. However, this categorical prediction should not be interpreted as evidence that these compounds have equivalent or improved in vivo bioavailability. To complement the in silico ADME analysis, the predictive BOILED-Egg model was applied to visualize the probable pharmacokinetic profile of all obtained metabolites (Figure 9). This graphical representation allows for the simultaneous evaluation of their potential for passive human intestinal absorption (HIA) and blood–brain barrier (BBB) permeation.

According to the predictions of this model, CBD (**1**) and metabolite **4** exhibit the potential for both high gastrointestinal absorption and efficient blood–brain barrier (BBB) permeation, as indicated by their location within the yellow region (BBB). The model further predicts that neither compound is subject to active efflux by P-glycoprotein (indicated by red dots, PGP-), which may favor their accumulation in brain tissue. This prediction for CBD (**1**) aligns with previous in vivo findings in mice, which confirmed that CBD is not a substrate for P-glycoprotein [44].

In contrast, for metabolites **2**–**3** and **5**–**9**, SwissADME analysis suggests that despite high predicted intestinal absorption (presence within the white HIA region), their capacity to cross the blood–brain barrier is low. Furthermore, the model forecasts that these molecules act as P-glycoprotein substrates (blue dots, PGP+), theoretically indicating active cellular efflux and further restricting their cerebral availability. Notably, all these insights are derived from in silico simulations integrating molecular lipophilicity (WLOGP) and topological polar surface area (TPSA), thereby illustrating only their potential in vivo pharmacokinetic behavior.

To determine the potential biological activities of the newly obtained compounds, an in silico analysis was performed using the PASS Online (Way2Drug) predictive tool [45]. The obtained results are summarized in Table 2.

PASS predictions suggested that compounds **7** and **8** may warrant future screening against selected biological targets. However, these predictions are computational and hypothesis-generating only. They do not provide evidence of biological activity, pharmacological efficacy, or mechanism of action. Overall, the reversed-phase UHPLC retention behaviour and the SwissADME predictions consistently indicate increased polarity and reduced predicted lipophilicity of compounds **7** and **8** relative to CBD. Nevertheless, these parameters do not directly establish aqueous solubility, intestinal permeability, systemic bioavailability, or pharmacological activity. Experimental solubility measurements, permeability studies, pharmacokinetic analyses, and biological assays are required to determine the actual effects of the introduced hydroxyl and methylglycosyl groups.

## 3. Materials and Methods

### 3.1. Chemicals

Reagents and solvents (analytical and HPLC grade) were purchased from Sigma-Aldrich (Merck Group, Darmstadt, Germany) and POCH (Avantor Performance Materials Poland, Gliwice, Poland). Reagents for microbiological media were obtained from POCH (Avantor Performance Materials Poland, Gliwice, Poland). The reaction substrate was purchased commercially from Biosynth (Staad, Switzerland).

### 3.2. Microorganisms

To investigate the potential for the enzymatic structural transformation of cannabidiol (**1**), sixteen strains of filamentous fungi were tested. The microorganisms were obtained from the culture collection of the Department of Food Chemistry and Biocatalysis at the Wrocław University of Environmental and Life Sciences (Wrocław, Poland). The following strains were used in the study: *Metarhizium robertsii* MU4, *Isaria farinosa* KCh KW 1.1, *I. tenuipes* MU35, *I. fumosorosea* KCh J2, *Beauveria bassiana* KCh BBT, *B. bassiana KCh* J 1.5, *B. caledonica* KCh J3.3, *Lecanicillium lecanii* NK3, *Mucor hiemalis* KCh W2, *M. hiemalis* AM 450, *M. hiemalis* AM 729, *Fusarium culmorum* AM 196, *Absidia cylindrospora* AM 336, *Aspergillus glaucus* AM 211, *Penicillium camemberti* AM 83, *Spicaria fusispora* AM 136. The fungal strains were maintained on agar slants at 4 °C. Before biotransformation experiments, the strains were transferred aseptically in a laminar flow cabinet from agar slants into 300 mL Erlenmeyer flasks containing 100 mL of sterile microbiological medium and incubated on a rotary shaker at 25 °C for approximately 3–7 days, depending on the growth rate of the strain. The obtained actively growing cultures were then transferred again into fresh 300 mL Erlenmeyer flasks containing 100 mL of sterile medium and used as pre-cultures for the biotransformation experiments.

### 3.3. Screening Studies

Screening studies were conducted in 300 mL Erlenmeyer flasks containing 100 mL of microbiological medium with the following composition: yeast extract (10 g/L), peptone (10 g/L), and glucose (70 g/L). The screening flasks were inoculated with approximately 0.5 mL of the pre-culture and incubated on a rotary shaker at 25 °C and 120 rpm. After 72 h, 10 mg of cannabidiol dissolved in dimethyl sulfoxide (initial CBD (**1**) concentration in DMSO: 40 mg/mL) was added to the flasks containing the grown culture, corresponding to a nominal substrate loading of 100 mg/L culture medium. On days 1, 3, and 7 of the process, approximately 10 mL of the culture broth was collected to monitor the progress of the biotransformation. To the collected sample, 5 mL of ethyl acetate was added, and the mixture was vigorously shaken using a vortex mixer for 5 min. Following extraction, the samples were centrifuged (Centrifuge 5810R, Eppendorf, Hamburg, Germany; 4000 rpm, 10 min). The separated organic layer was evaporated to dryness, and the residue was dissolved to prepare a sample for HPLC analysis. All experimental trials were performed in triplicate. Control experiments were performed to verify the stability of cannabidiol (CBD) (**1**) under the cultivation conditions and to establish the chromatographic background of the fungal cultures. CBD (**1**) incubated in sterile culture medium in the absence of fungal biomass remained unchanged throughout the incubation period, indicating that no detectable non-enzymatic transformation occurred under the applied conditions. In addition, fungal cultures grown under identical conditions without CBD (**1**) were routinely analysed to determine the endogenous metabolite profiles. These control chromatograms were used during UHPLC-DAD analysis to distinguish endogenous fungal metabolites from CBD biotransformation products.

### 3.4. HPLC Analysis

Chromatographic analyses were performed using an Ultimate 3000 UHPLC+ system (Thermo Scientific, Waltham, MA, USA) equipped with a DGP-3600A dual pump, a TCC3200 thermostatted column compartment, a WPS-3000 autosampler, and a DAD detector. Separation was carried out on a Thermo Scientific Acclaim RSLC Polar Advantage II C-18 UHPLC column (2.1 mm × 100 mm, 2.2 µm; Thermo Scientific). The analysis parameters were as follows: column temperature of 28 °C, flow rate of 0.7 mL/min, and analysis time of 5 min. Gradient elution was applied, where mobile phase A consisted of 0.1% formic acid in water, and mobile phase B consisted of 0.1% formic acid in acetonitrile. The gradient elution was as follows: 0.0–2.0 min: isocratic 30% phase B; 2.0–2.2 min: linear increase in phase B to 98%; 2.2–3.2 min: isocratic step with 98% phase B; 3.2–3.3 min: return to initial conditions (30% phase B); 3.3–5.0 min: column equilibration (30% phase B). Spectra were recorded in the range of λ = 190–450 nm, with wavelengths of 210 nm and 275 nm used as diagnostic for monitoring the substrate and product peaks. Data collection rate was 25 Hz. Relative CBD peak depletion was estimated from UHPLC-DAD peak areas as the percentage decrease in the substrate peak area relative to the total peak area of the substrate and detected biotransformation products. Product distribution was estimated from the relative peak areas of individual metabolites. Because standards were not available for all metabolites, these values should be considered semi-quantitative. The characteristic UV absorption profiles recorded using the DAD detector, together with retention times and comparison with chromatograms of fungal cultures without CBD, were used to distinguish CBD-derived metabolites from endogenous fungal components. The UHPLC-DAD analysis was performed on ethyl acetate extracts of the culture broth. No internal standard was used, and extraction recoveries were not determined for CBD or individual metabolites. The aqueous phase and fungal biomass were not analysed quantitatively for residual, adsorbed, or non-extracted compounds. Therefore, the obtained peak-area data do not provide a complete mass balance and cannot distinguish absolute biotransformation from possible substrate adsorption, incomplete extraction, or compound-dependent partitioning between the aqueous and organic phases. Only peaks assigned to CBD-derived metabolites on the basis of retention time, UV spectra, comparison with fungal controls, and, for isolated compounds, NMR/HRESI-MS confirmation were included in the relative chromatographic analysis. Endogenous fungal peaks and unidentified background components were excluded. Accordingly, the reported values should be interpreted only as apparent CBD depletion and relative UHPLC-DAD peak-area composition of the analysed extracts. The structures of isolated metabolites were subsequently confirmed by NMR spectroscopy and high-resolution mass spectrometry.

### 3.5. Scale-Up Biotransformation

Biotransformation on a larger scale was conducted to obtain a sufficient amount of products for their isolation and structural identification. The process was carried out in 2 L flasks containing 500 mL of microbiological medium (identical composition to the screening studies). The scale-up flasks were inoculated with the corresponding actively growing pre-culture prepared as described above. After biomass growth (incubation time was strain-specific, averaging approximately 48 h), 100 mg of cannabidiol (**1**) dissolved in DMSO (40 mg/mL) was added to the culture. After 7 days, 10 mL of the culture broth was collected, extracted with ethyl acetate, evaporated, and analysed by UHPLC to confirm the reaction progress. The cultures were terminated on the 10th day of the process. The culture broth was extracted three times with ethyl acetate (using 250 mL of solvent each time). After each extraction, the mixture was centrifuged to separate the organic layer. The collected organic phases were combined, dried over anhydrous MgSO_4_, filtered, and concentrated using a rotary evaporator. The resulting crude extracts were used for further studies.

### 3.6. Purification of Biotransformation Products

The crude extracts obtained from the biotransformation were purified using a PuriFlash PF430 automated flash column chromatography system coupled with a UV detector (Interchim, Montluçon, France). The initial separations were performed using PF-30SIHP-30 µm silica columns (Interchim, Montluçon, France) employing a gradient elution, with a mobile phase consisting of chloroform and 0.1% HCOOH in methanol. The elution gradient was adjusted empirically during the chromatographic runs. Fractions were collected according to UV detection and subsequently analysed by UHPLC-DAD; fractions containing the same target metabolite, as indicated by retention time and UV spectrum, were combined and evaporated. When necessary, the collected fractions containing the biotransformation products were subjected to further purification, again using the PuriFlash PF430 system, but with a significantly modified setup. A reversed-phase system was employed, utilizing a C18-15 µm (octadecylsilane, ODS) column and a mobile phase composed of 0.1% HCOOH in water and 0.1% HCOOH in methanol. The application of reversed-phase chromatography enabled the direct replication of the separation conditions previously established during the analytical UHPLC runs. Prior to the preparative separation using the PuriFlash system, UHPLC analyses were conducted under the following conditions: Column used was Thermo Scientific Acclaim RSLC Polar Advantage II C-18 UHPLC column (2.1 mm × 100 mm, 2.2 µm); temperature of 25 °C, flow rate of 0.5 mL/min, and analysis time of 12 min. Gradient elution was applied, where mobile phase A consisted of 0.1% formic acid in water, and mobile phase B consisted of 0.1% formic acid in methanol. The gradient elution was as follows: 0.0–8.0 min: linear increase in phase B from 5% to 100%; 8.0–9.5 min: isocratic step with 100% phase B; 9.5–11.0 min: return to initial conditions (5% phase B); 11.0–12.0 min: column equilibration (5% phase B). Instrumentation was consistent with the description provided in Section 3.4.

### 3.7. Intermediate Metabolite Experiment

To a pre-grown shake-flask culture of *Isaria fumosorosea* KCh J2 (medium volume: 50 mL; composition: 10 g/L bacteriological peptone, 10 g/L yeast extract, and 70 g/L glucose), 1 mg of 2′-*O*-(β-D-glucopyranosyl)-cannabidiol (**2**) dissolved in 25 µL of DMSO was added. After two days of incubation, samples were collected and prepared for UHPLC analysis according to the aforementioned procedure. The experiment was performed in triplicate.

### 3.8. NMR and HRESI-MS Analysis

^1^H and ^13^C NMR, DEPT-135, and two-dimensional NMR experiments (^1^H–^1^H COSY as well as ^1^H–^13^C HSQC and HMBC) were recorded using a Bruker DRX Avance II 600 spectrometer (600 MHz; Bruker, Billerica, MA, USA) and Bruker DRX Avance 800 (800 MHz; Bruker, Billerica, MA, USA) using DMSO-d_6_ as solvent. The spectra were processed with MestReNova software (v9.0, Mestrelab Research, Santiago de Compostela, Spain).

The spectroscopic data of substrate **1** and purified metabolites **2**–**6** and **9** as well as isolated yields are as follows:

**Cannabidiol 1:** Spectral data (Supplementary Figures S1–S5): ^1^H NMR (600 MHz, DMSO-d_6_) δ [ppm]: 6.02 (s, 2H, H-3′ and H-5′ [overlapping signals]), 5.09 (brs, 1H, H-2), 4.50 (d, *J* = 2.8 Hz, 1H, H-9b), 4.41 (dq, *J* = 2.9, 1.4 Hz, 1H, H-9a), 3.86–3.81 (m, 1H, H-1), 3.03 (ddd, *J* = 13.1, 10.5, 2.7 Hz, 1H, H-6), 2.30 (t, *J* = 7.6 Hz, 2H, H-1″), 2.15–2.07 (m, 1H, H-4b), 1.95–1.90 (m, 1H, H-4a), 1.72–1.67 (m, 1H, H-5a), 1.62 (td, *J* = 12.4, 5.4 Hz, 1H, H-5b), 1.61 (m, 3H, H-7), 1.59 (s, 3H, H-10), 1.48 (p, *J* = 7.5 Hz, 2H, H-2″), 1.33–1.21 (m, 4H, H-3″ and H-4″ [overlapping signals]), 0.87 (t, *J* = 7.1 Hz, 3H, H-5″).

^13^C NMR (150 MHz, DMSO-d_6_) δ [ppm]: 156.7 (C-2′ and C-6′ [overlapping signals]), 149.6 (C-8), 140.6 (C-4′), 130.5 (C-3), 127.3 (C-2), 114.6 (C-1′), 110.1 (C-9), 107.1 (C-3′ and C-5′ [overlapping signals]), 44.1 (C-6), 36.0 (C-1), 35.4 (C-1″), 31.5 (C-3″), 30.8 (C-4), 30.8 (C-2″), 29.9 (C-5), 23.7 (C-7), 22.5 (C-4″), 19.7 (C-10), 14.4 (C-5″). UV (MeOH) λ_max_: 213.4, 273.0 nm.

**Compound 2:** Isolated as a colourless amorphous solid; 24.3 mg (16.0%, UHPLC purity 97.9%); (*M. hiemalis* AM 450) and 13.7 mg (9.0%, UHPLC purity 97.2%) (*M. hiemalis* KCh W2), respectively. This product was also detected exclusively via UHPLC chromatograms during biotransformation by *Isaria fumosorosea* KCh J2. Spectral data (Supplementary Figures S6–S16):

^1^H NMR (600 MHz, DMSO-d_6_) δ [ppm]: 6.39 (s, 1H, H-3′), 6.24 (s, 1H, H-5′), 5.14 (s, 1H, H-2), 4.69 (d, *J* = 7.3 Hz, 1H, H-1‴), 4.53 (d, 1H, *J* = 2.7 Hz, H-9b), 4.39 (brs, 1H, H-9a), 4.00–3.95 (m, 1H, H-1), 3.70 (ddd, *J* = 11.8, 5.3, 2.1 Hz, 1H, H-6‴a), 3.46 (dt, *J* = 11.9, 6.1 Hz, 1H, H-6‴b), 3.29–3.21 (m, 3H, H-2‴, H-3‴ and, H-4‴ [overlapping signals]), 3.19–3.13 (m, 1H, H-5‴), 3.14–3.04 (m, 1H, H-6) 2.38 (td, *J* = 7.3, 1.5 Hz, 2H, H-1″), 2.17–2.09 (m, 1H, H-4a), 1.93 (dd, 1H, H-4b), 1.72–1.67 (m, 1H, H-5b), 1.64 (td, *J* = 12.3, 5.3 Hz, 1H, H-5a), 1.61 (s, 3H, H-7), 1.59 (s, 3H, H-10), 1.51 (p, *J* = 7.6 Hz, 2H, H-2″), 1.35–1.23 (m, 4H, H-3″ and H-4″ [overlapped signals]), 0.85 (t, *J* = 7.1 Hz, 3H, H-5″).

^13^C NMR (150 MHz, DMSO-d_6_) δ [ppm]: 157.1 (C-2′), 156.4 (C-6′), 149.3 (C-8), 141.1 (C-4′), 131.2 (C-3), 127.4 (C-2), 117.2 (C-1′), 110.4 (C-9), 109.9 (C-5′), 106.9 (C-3′), 101.5 (C-1‴), 77.6 (C-2‴), 77.1 (C-3‴), 74.2 (C-4‴), 70.4 (C-5‴), 61.3 (C-6‴), 44.1 (C-6), 36.2 (C-1), 35.6 (C-1″), 31.5 (C-3″), 30.8 (C-4), 30.6 (C-2″), 29.8 (C-5), 23.7 (C-7), 22.5 (C-4″), 19.9 (C-10), 14.4 (C-5″). HRESI-MS: [M + H]+; *m*/*z* 477.2850 (calcd. 477.2847; mass error = −0.67 ppm). UV (MeOH) λ_max_: 206.0, 274.4 nm.

**Compound 3:** Isolated as a pale yellow glassy film; 15.2 mg (9.7%, UHPLC purity 91.2%) (*M. hiemalis* KCh W2) and 2.5 mg (1.6% UHPLC purity 90.1%) (*M. hiemalis* AM 450), respectively. Spectral data (Supplementary Figures S17–S27):

^1^H NMR (800 MHz, DMSO-*d*_6_) δ [ppm]: 6.37 (s, 1H, H-3′), 6.25 (s, 1H, H-5′), 5.12 (s, 1H, H-2), 4.68 (d, *J* = 7.5 Hz, 1H, H-1‴), 4.53 (brs, 1H, H-9a), 4.38 (s, 1H, H-9b), 3.99–3.94 (m, 1H, H-1), 3.68 (brd, *J* = 11.8 Hz, 1H, H-6‴a), 3.61–3.57 (m, 1H, H-4″), 3.49–3.56 (m, 1H, H-6‴b), 3.28–3.19 (m, 3H, H-2‴, H-3‴ and, H-4‴ [overlapping signals]), 3.17–3.14 (m, 1H, H-5‴), 3.10–3.08 (m, 1H, H-6), 2.41–2.31 (m, 2H, H-1″), 2.12 (m, 1H, H-4a), 1.93 (m, 1H, H-4b), 1.68 (m, 1H, H-5a), 1.63 (td, *J* = 12.4, 5.3 Hz, 1H, H-5b), 1.60 (s, 3H, H-7), 1.58 (s, 3H, H-10), 1.58–1.55 (m, 1H, H-2″a), 1.53–1.46 (m, 1H, H-2″b), 1.38–1.33 (m, 1H, H-3″a), 1.32–1.28 (m, 1H, H-3″b), 1.03 (d, *J* = 6.3 Hz, 3H, H-5″).

^13^C NMR (201 MHz, DMSO-d_6_) δ [ppm]: 157.2 (C-2′), 156.4 (C-6′), 149.3 (C-8), 141.2 (C-4′), 131.2 (C-3), 127.4 (C-2), 115.0 (C-1′), 110.4 (C-9), 110.1 (C-5′), 106.8 (C-3′), 101.5 (C-1‴), 77.1 (C-2‴), 76.8 (C-3‴), 73.7 (C-4‴), 70.1 (C-5‴), 66.2 (C-4″), 61.1 (C-6‴), 44.1 (C-6), 39.3 (C-3″), 36.2 (C-1), 35.8 (C-1″), 30.8 (C-4), 29.8 (C-5), 27.3 (C-2″), 24.1 (C-5″), 23.7 (C-7), 19.8 (C-10). HRESI-MS: [M + H]+; *m*/*z* 493.2804 (calcd. 493.2796; mass error = −1.62 ppm). UV (MeOH) λ_max_: 198.7, 219.1, 274.4 nm.

**Compound 4:** Isolated as a pale yellow glassy film; 2.3 mg (2.2% UHPLC purity 90.6%) (*M. hiemalis* AM 450). This product was also detected exclusively via UHPLC chromatograms during biotransformation by *M. hiemalis* KCh W2. Spectral data (Supplementary Figures S28–S36):

^1^H NMR (800 MHz, DMSO-d_6_) δ [ppm]: 6.02 (s, 2H, H-3′ and H-5′ [overlapping signals]), 5.09 (brs, 1H, H-2), 4.50 (d, *J* = 2.7 Hz, 1H, H-9a), 4.41 (dd, *J* = 2.9, 1.5 Hz, 1H, H-9b), 3.84–3.82 (m, 1H, H-1), 3.62–3.54 (m, 1H, H-4″), 3.03 (ddd, *J* = 13.0, 10.5, 2.7 Hz, 1H, H-6), 2.34–2.24 (m, 2H, H-1″), 2.14–2.07 (m, 1H, H-4a), 1.95–1.90 (m, 1H, H-4b), 1.70–1.67 (m, 1H, H-5a), 1.64–1.61 (m, 1H, H-5b), 1.60 (brs, 3H, H-7), 1.59 (brs, 3H, H-10), 1.55 (dddd, *J* = 14.3, 9.1, 7.5, 3.4 Hz, 1H, H-2″a), 1.50–1.41 (m, 1H, H-2″b), 1.34 (dddd, *J* = 12.5, 10.3, 7.2, 5.1 Hz, 1H, H-3″a), 1.29 (ddd, *J* = 10.6, 5.3, 2.1 Hz, 1H, H-3″b), 1.03 (d, *J* = 6.2 Hz, 3H, H-5″).

^13^C NMR (201 MHz, DMSO-d_6_) δ [ppm]: 156.7 (C-2′ and C-6′ [overlapping signals]), 149.6 (C-8), 140.7 (C-4′), 130.6 (C-3), 127.3 (C-2), 114.7 (C-1′), 110.3 (C-9), 107.2 (C-3′ and C-5′), 66.2 (C-4″), 44.2 (C-6), 39.3 (C-3″), 36.1 (C-1), 35.6 (C-1″), 30.8 (C-4), 30.0 (C-5), 27.4 (C-2″), 24.1 (C-5″), 23.7 (C-7), 19.8 (C-10). HRESI-MS: [M + H]+; *m*/*z* 331.2274 (calcd. 331.2268; mass error = −1.81 ppm). UV (MeOH) λ_max_: 212.2, 275.0 nm.

**Compound 5:** Isolated as a colourless amorphous solid; 6.5 mg (5.9% UHPLC purity 93.8%) (*M. hiemalis* KCh W2) and 1.7 mg (1.5% UHPLC purity 93.6%) (*M. hiemalis* AM 450). Spectral data (Supplementary Figures S37–S45):

^1^H NMR (600 MHz, DMSO-d_6_) δ [ppm]: 6.02 (s, 1H, H-3′ or H-5′), 5.99 (s, 1H, H-3′ or H-5′), 5.06 (q, *J* = 1.7 Hz, 1H, H-2), 4.56 (d, *J* = 6.6 Hz, 1H, 4-OH), 4.48 (d, *J* = 2.8 Hz, 1H, H-9b), 4.40 (dq, *J* = 2.9, 1.4 Hz, 1H, H-9a), 4.32 (d, *J* = 4.7 Hz, 1H, 4″-OH), 4.11–4.05 (m,1H, H-4), 3.87–3.82 (m, 1H, H-1), 3.60–3.54 (m, 1H, H-4″), 3.18 (ddd, *J* = 13.1, 10.4, 2.4 Hz, 1H, H-6), 2.34–2.22 (m, 2H, H-1″), 1.88 (ddd, *J* = 12.0, 5.7, 2.4 Hz, 1H, H-5a), 1.63–1.61 (m, 3H, H-10), 1.58 (brs, 3H, H-7), 1.57–1.49 (m, 2H, H-2″a and H-5b [overlapping signals]), 1.49–1.40 (m, 1H, H-2″b), 1.36–1.21 (m, 2H, H-3″), 1.02 (d, *J* = 6.2 Hz, 3H, H-5″).

^13^C NMR (151 MHz, DMSO-d_6_) δ [ppm]: 157.0 (C-2′ or C-6′), 155.6 (C-2′ or C-6′), 148.2 (C-8), 140.4 (C-4′), 137.7 (C-3), 128.3 (C-2), 113.8 (C-1′), 109.9 (C-9), 107.6 (C-3′ or C-5′), 106.1 (C-3′ or C-5′), 69.1(C-4), 65.7 (C-4″), 43.9 (C-6), 38.8 (C-3″), 36.0 (C-1), 35.1 (C-1″), 31.0 (C-5), 27.5 (C-2″), 24.2 (C-5″), 19.7 (C-7), 19.5 (C-10). HRESI-MS: [M + H]+; *m*/*z* 347.2219 (calcd. 347.2217; mass error = −0.62 ppm). UV (MeOH) λ_max_: 197.3, 216.6, 274.0 nm.

**Compound 6:** Isolated as a colourless amorphous solid; 1.8 mg (1.6% UHPLC purity 87.2%) (*M. hiemalis* KCh W2) and 0.5 mg (0.4%, UHPLC purity 89.0%) (*M. hiemalis* KCh 450). Spectral data (Supplementary Figures S46–S54):

^1^H NMR (800 MHz, DMSO-d_6_) δ [ppm]: 6.03 (s, 2H, H-3′, H-5′), 5.29 (q, *J* = 1.7 Hz, 1H, H-2), 4.52 (brd, *J* = 5.7 Hz, 7-OH), 4.51 (d, *J* = 2.8 Hz, 1H, H-9b), 4.42 (dq, *J* = 3.0, 1.5 Hz, 1H, H-9a), 4.33 (brd, *J* = 4.7 Hz, 4″-OH), 3.88–3.84 (m, 1H, H-1), 3.81–3.74 (m, 2H, H-7), 3.60–3.55 (m, 1H, H-4″), 3.08 (ddd, *J* = 13.0, 10.5, 2.6 Hz, 1H, H-6), 2.35–2.25 (m, 2H, H-1″), 2.07–2.03 (m, 2H, H-4), 1.75–1.71 (m, 1H, H-5a), 1.60 (brs, 3H, H-10), 1.60–1.50 (m, 2H, H-5b and H-2″a [overlapping signals]), 1.50–1.42 (m, 1H, H-2″b), 1.37–1.31 (m, 1H, H-3″a), 1.31–1.26 (m, 1H, H-3″b), 1.03 (d, *J* = 6.2 Hz, 3H, H-5″).

^13^C NMR (200 MHz, DMSO-d_6_) δ [ppm]: 156.6 (C-2′ and C-6′ [overlapping signals]), 149.6 (C-8), 140.8 (C-4′),135.5 (C-3), 126.7 (C-2), 114.4 (C-1′), 110.1 (C-9), 107.0 (C-3′ and C-5′ [overlapping signals]), 66.1 (C-4″), 65.4 (C-7), 44.4 (C-6), 39.2 (C-3″), 35.5 (C-1), 35.4 (C-1″), 29.7 (C-5), 27.3 (C-2″), 26.3 (C-4), 23.9 (C-5″), 19.6 (C-10). HRESI-MS: [M + H]+; *m*/*z* 347.2220 (calcd. 347.2217; mass error = −0.58 ppm). UV (MeOH) λ_max_: 207.3, 274.6 nm.

**Compound 9:** Isolated as a pale yellow glassy film; 1.9 mg (1.5% UHPLC purity 97.9%) (*Metarhizium robertsii* MU4). Spectral data (Supplementary Figures S74–S82):

^1^H NMR (800 MHz, DMSO-d_6_) δ [ppm]: 6.06 (brs, 1H H-3′),6.02 (brs, 1H, H-5′), 5.07 (q, *J* = 1.7 Hz, 1H, H-2), 4.48 (d, *J* = 2.7 Hz, 1H, H-9a), 4.42–4.39 (m, 1H, H-9b), 4.12–4.06 (m, 1H, H-4), 3.86 (dq, *J* = 10.7, 2.8 Hz, 1H, H-1), 3.55–3.50 (m, 1H, H-2″), 3.18 (ddd, *J* = 13.1, 10.4, 2.4 Hz, 1H, H-6), 2.42 (dd, *J* = 13.3, 6.6 Hz, 1H, H-1″a), 2.31 (dd, *J* = 13.4, 6.2 Hz, 1H, H-1″b), 1.89 (ddd, *J* = 12.1, 5.8, 2.5 Hz, 1H, H-5a), 1.63 (brs, 3H, H-7), 1.59 (s, 3H, H-10), 1.58–1.53 (m, 1H, H-5b), 1.45–1.38 (m, 1H, H-4″a), 1.33–1.27 (m, 1H, H-3″a), 1.28–1.20 (m, 2H, H-3″ and H-4″ [overlapping signal]), 0.83 (t, *J* = 7.1 Hz, 3H, H-5″).

^13^C NMR (201 MHz, DMSO-d_6_) δ [ppm]: 157.1 (C-2′ or C-6′),155.9 (C-2′ or C-6′), 148.7 (C-8), 138.3 (C-4′), 135.0 (C-3), 128.8 (C-2), 114.5 (C-1′), 110.3 (C-9),108.6 (C-3′ or C-5′), 107.7 (C-3′ or C-5′), 71.2 (C-2″), 69.6 (C-4), 44.4 (C-1″ and C-6 [overlapping signal]), 40.6 (C-5), 39.3 (C-3″), 36.6 (C-1), 19.6 (C-7, C-10 [overlapping signals]), 18.9 (C-4″), 14.5 (C-5″). HRESI-MS: [M + H]+; *m*/*z* 347.2221 (calcd. 347.2217; mass error = −1.15 ppm). UV (MeOH) λ_max_: 206.3, 275.0 nm.

For the high-resolution mass spectrometry, the analysed compounds were dissolved in methanol with the addition of 0.1% formic acid, and 1 μL aliquots were subsequently concentrated by liquid chromatography on an ACQUITY BEH-C8 column (100 mm × 2.1 mm, 1.7 μm) prior to mass spectrum recording. The high-resolution mass spectra were recorded using a Xevo G3 QToF instrument (Waters Corporation, Milford, CT, USA) equipped with an electrospray ionization source operating in positive ionization mode (ESI+, 3 kV).

The mass errors are expressed as ppm value and were calculated according to the following equation:

$$\Delta m=\left(\;\frac{m_{theoretical\;}-\;m_{measured\;}\;}{m_{theoretical\;}}\right)\times \;10^{6}\;\;\;[ppm]$$

### 3.9. Statistical Analysis

All biotransformation experiments were performed in three independent biological replicates, corresponding to separate shake-flask cultures prepared and processed independently. UHPLC-DAD analysis was performed for each extract obtained from these biological replicates. Results are expressed as mean ± standard deviation (SD). Data normality was assessed using the Shapiro–Wilk test prior to analysis; variables were log-transformed using the natural logarithm to normalize skewed distributions stemming from the experimental design [46]. For comparisons between two groups, Student’s t-test was used when the normality assumption was met. For comparisons involving more than two groups or time points, one-way ANOVA was applied. Differences were considered statistically significant at *p* < 0.05. Statistical analyses were performed using Microsoft Excel with the Analysis ToolPak and Jamovi software, version 2.6.44 [47].

## 4. Conclusions

These studies demonstrated the capability of filamentous fungi to microbially transform cannabidiol (**1**). Among the tested strains, thirteen showed the ability to biotransform CBD under the applied conditions. Notably, strains belonging to the genera *Metarhizium*, *Isaria*, and *Beauveria* showed pronounced relative depletion of the CBD chromatographic peak and diverse metabolite profiles. Furthermore, this research led to the isolation of two previously undescribed cannabidiol derivatives (**7** and **8**), along with six previously reported structures. The filamentous fungi demonstrated the capacity for regioselective hydroxylation, as well as glycosylation, and methylglycosylation, highlighting their usefulness as whole-cell biocatalysts for generating structurally diverse CBD derivatives. These studies also suggest a metabolic pathway for the *Isaria fumosorosea* strain in which methyl glycosides are not formed via a single simultaneous enzymatic process, but rather through two distinct, sequential processes occurring over time. However, the observed biotransformation profiles were strongly strain-dependent, and the present experiments were performed at laboratory shake flask scale. Therefore, direct transfer of these results to larger-scale production should be considered preliminary. Further optimization of fermentation parameters, biomass growth, substrate loading, product recovery, and downstream purification will be required before the practical scalability of this approach can be assessed. In addition, the translational relevance of the obtained metabolites will depend on future experimental verification of their aqueous solubility, permeability, pharmacokinetic behaviour, safety, and biological activity. In conclusion, the biotransformation of cannabidiol (**1**) yielded structurally diverse derivatives with increased polarity, as supported by their reversed-phase UHPLC retention behaviour and lower predicted lipophilicity. However, these findings should not be interpreted as evidence of improved aqueous solubility, bioavailability, or biological activity. Compounds **7** and **8** represent previously undescribed CBD derivatives that warrant further physicochemical, pharmacokinetic, and biological evaluation.

## Figures and Tables

**Table 1:** Apparent CBD depletion estimated from UHPLC-DAD peak areas in ethyl acetate extracts of the tested fungal cultures.

Strain	Substrate Depletion [%] *	Main Products ***
*Metarhizium robertsii* MU4	91.81 ± 5.71 **	**7**, **9**
*Isaria farinosa* KCh KW 1.1	73.01 ± 2.69	**7**
*Isaria tenuipes* MU35	68.26 ± 17.24	**7**
*Isaria fumosorosea* KCh J2	82.95 ± 9.61 **	**2**, **7**, **8**
*Beauveria bassiana* KCh BBT	49.04 ± 18.35	**7**
*Beauveria bassiana* KCh J 1.5	20.57 ± 16.22	**7**
*Lecanicillium lecanii* NK3	6.55 ± 1.58	**2**
*Beauveria caledonica* KCh J33	50.15 ± 13.74	**7**
*Mucor hiemalis* KCh W2	98.74 ± 0.62 **	**2**–**6**
*Mucor hiemalis* AM 450	78.19 ± 11.98 **	**2**–**6**
*Mucor hiemalis* AM 729	0.00 ± 0.00	-
*Fusarium culmorum* AM 196	6.10 ± 0.33	**4**
*Absidia cylindrospora* AM 336	28.64 ± 1.67	**4**
*Aspergillus glaucus* AM 211	0.00 ± 0.00	-
*Penicillium camemberti* AM 83	32.35 ± 9.18	**2**, **4**
*Spicaria fusispora* AM 136	0.00 ± 0.00	-

* The presented results are based on UHPLC-DAD peak areas in ethyl acetate extracts and represent apparent CBD depletion relative to the detected CBD-derived chromatographic peaks. They should not be interpreted as absolute conversion, product yield, extraction recovery, or mass balance. Unidentified endogenous fungal peaks were not included in the calculation. ** Microbial strains were selected for scaled-up biotransformation. *** Detected by UHPLC-DAD analysis.

**Table 2:** PASS Online predictive analysis results obtained for metabolites **7** and **8**.

Potential Biological Activity	Metabolite 7 (Pa/Pi)	Metabolite 8 (Pa/Pi)
Hepatoprotection	0.921/0.002	0.923/0.002
Antiprotozoal (Leishmania)	0.920/0.003	0.931/0.002
Anticarcinogenic	0.849/0.004	0.850/0.004
Antifungal	0.798/0.005	0.791/0.005
Proliferative diseases	0.749/0.004	0.717/0.005
Caspase 3 stimulant	0.808/0.005	—
Antineoplastic	0.799/0.012	0.799/0.012

Pa/Pi—Probability of a compound showing activity (Pa) relative to the probability of it being inactive (Pi).

## Data Availability

The original contributions presented in this study are included in the article/Supplementary Materials. Further inquiries can be directed to the corresponding authors.

## Supplementary Materials

The following supporting information can be downloaded at: https://www.mdpi.com/article/10.3390/ijms27156884/s1.
