# Supplementary material for: Whole-Cell Transformation of Cannabidiol by Selected Filamentous Fungi into Novel Polar Derivatives
Source: Int J Mol Sci. 2026 Aug 1;27(15):6884. doi: 10.3390/ijms27156884 (PMC13467004; doi:10.3390/ijms27156884)
Supplement: Supplementary file 1 [file ijms-27-06884-s001.zip › ijms-4424842-supplementary.pdf]

Supplementary Data

Whole-Cell Transformation of Cannabidiol by Selected Filamentous Fungi into Novel Polar Derivatives

Daniel Łój 1,* , Tomasz Janeczko 1 , Mariusz A. Bromke 2 and Tomasz Tronina 1,*

1 Department of Food Chemistry and Biocatalysis, Wrocław University of Environmental and Life Sciences, Norwida 25, 50-375 Wrocław, Poland; tomasz.janeczko@upwr.edu.pl

2 Department of Biochemistry and Immunochemistry, Wroclaw Medical University, ul Chałubinskiego 10, 50-368 Wrocław, Poland; mariusz.bromke@umw.edu.pl

* Correspondence: daniel.loj@upwr.edu.pl (D.Ł.); tomasz.tronina@upwr.edu.pl (T.T.)

Table of contents

1. Cannabidiol (1) 4

Figure S1. UHPLC-DAD chromatogram of substrate (CBD) (**1**) 4

Figure S2. UV spectrum of substrate (CBD) (**1**) 4

Figure S3. ^{1}H NMR spectrum of cannabidiol **1** 5

Figure S4. ^{1}H NMR (0.5-2.5ppm) spectrum of cannabidiol **1** 5

Figure S5. ^{13}C NMR spectrum of cannabidiol **1** 6

2. Metabolite 2: 2’-O-(β-D-glucopyranosyl)-cannabidiol 7

Figure S6. UHPLC-DAD chromatogram of metabolite **2** 7

Figure S7. UV spectrum of metabolite **2** 7

Figure S8. Mass spectrum of metabolite **2** 7

Figure S9. ^{1}H NMR spectrum of metabolite **2** 8

Figure S10. ^{1}H NMR spectrum of metabolite **2** (0.8-2.5ppm) 8

Figure S11. ^{1}H NMR spectrum of metabolite **2** (2.6-6.4 ppm) 9

Figure S12. ^{13}C NMR spectrum of metabolite **2** 9

Figure S13. COSY (^{1}H-^{1}H) NMR spectrum of metabolite **2** 10

Figure S14. HSQC (1H-13C) NMR spectrum of metabolite **2** 10

Figure S15. HMBC (long distance correlation ^{1}H-^{13}C) spectrum of metabolite **2** 11

Figure S16. HMBC correlation between C1 carbon of glucose and C2 carbon of aromatic ring. 11

3. Metabolite 3: 2’-O-(β-D-glucopyranosyl)-4”-hydroxycannabidiol 12

Figure S17. UHPLC-DAD chromatogram of metabolite **3** 12

Figure S18. UV spectrum of metabolite **3** 12

Figure S19. Mass spectrum of metabolite **3** 12

Figure S20. ^{1}H NMR spectrum of metabolite **3** 13

Figure S21. ^{1}H NMR (0.4-3.0ppm) spectrum of metabolite **3** 14

Figure S22. ^{1}H NMR spectrum of metabolite **3** (3.0-5.2 ppm) 14

Figure S23. 13C NMR spectrum of metabolite **3** 15

Figure S24. COSY (^{1}H-^{1}H) NMR spectrum of metabolite **3** 15

Figure S25. HSQC (^{1}H-^{13}C) NMR spectrum of metabolite **3** 16

Figure S26. HMBC (long distance correlation ^{1}H-^{13}C) spectrum of metabolite **3** 16

Figure S27. HMBC correlation between C1 carbon of glucose and C2 carbon of aromatic ring in metabolite **3** 17
4. **Metabolite 4: 4”-hydroxycannabidiol** 17
Figure S28. UHPLC-DAD chromatogram of metabolite **4** 17
Figure S29. UV spectrum of metabolite **4** 17
Figure S30. Mass spectrum of metabolite **4** 17
Figure S31. ^{1}H NMR spectrum of metabolite **4** 18
Figure S32. ^{1}H NMR (0.7-2.5) spectrum of metabolite **4** 18
Figure S33. 13C NMR spectrum of metabolite **4** 19
Figure S34. COSY (^{1}H-^{1}H) NMR spectrum of metabolite **4** 19
Figure S35. HSQC (^{1}H-^{13}C) NMR spectrum of metabolite **4** 20
Figure S36. HMBC (long distance correlation ^{1}H-^{13}C) spectrum of metabolite **4** 20
5. **Metabolite 5: 4,4”-dihydroxycannabidiol** 21
Figure S37. UHPLC-DAD chromatogram of metabolite **5** 21
Figure S38. UV spectrum of metabolite **5** 21
Figure S39. Mass spectrum of metabolite **5** 21
Figure S40. ^{1}H NMR spectrum of metabolite **5** 22
Figure S41. ^{1}H NMR (0.7-2.5) spectrum of metabolite **5** 22
Figure S42. ^{13}C NMR spectrum of metabolite **5** 23
Figure S43. COSY (^{1}H-^{1}H) NMR spectrum of metabolite **5** 23
Figure S44. HSQC (1H-13C) NMR spectrum of metabolite **5** 24
Figure S45. HMBC (long distance correlation ^{1}H-^{13}C) spectrum of metabolite **5** 24
6. **Metabolite 6: 7,4”-dihydroxycannabidiol** 25
Figure S46. UHPLC-DAD chromatogram of metabolite **6** 25
Figure S47. UV spectrum of metabolite 6 25
Figure S48. Mass spectrum of metabolite **6** 25
Figure S49. ^{1}H NMR spectrum of metabolite **6** 26
Figure S50. ^{1}H NMR (0.8-2.8) spectrum of metabolite **6** 26
Figure S51. ^{13}C NMR spectrum of metabolite **6** 27
Figure S52. COSY (^{1}H-^{1}H) NMR spectrum of metabolite **6** 27
Figure S53. HSQC (^{1}H-^{13}C) NMR spectrum of metabolite **6** 28
Figure S54. HMBC (long distance correlation ^{1}H-^{13}C) spectrum of metabolite **6** 28
7. **Metabolite 7: 2’-*O*-(4’”-*O*-methyl-β-D-glucopyranosyl)cannabidiol** 29
Figure S55. UHPLC-DAD chromatogram of metabolite **7** 29
Figure S56. UV spectrum of metabolite **7** 29
Figure S57. Mass spectrum of metabolite **7** 29
Figure S58. ^{1}H NMR spectrum of metabolite **7** 30
Figure S59. ^{1}H NMR (0.7-2.6) spectrum of metabolite **7** 30

Figure S60. 13C NMR spectrum of metabolite **7** 31

Figure S61. COSY (^1H-^1H) NMR spectrum of metabolite **7** 31

Figure S62. HSQC (^1H-^{13}C) NMR spectrum of metabolite **7** 32

Figure S63. HMBC (long distance correlation ^1H-^{13}C) NMR spectrum of metabolite **7** 32

Figure S64. HMBC correlation between C1 carbon of glucose and C2 carbon of aromatic ring. Correlation between -OCH3 methoxyl group and C4 carbon of glucose. 33

8. Metabolite 8: 2'-*O*-(4'''-*O*-methyl-β-D-glucopyranosyl) 5''-hydroxycannabidiol 34

Figure S65. UHPLC-DAD chromatogram of metabolite **8** 34

Figure S66. UV spectrum of metabolite **8** 34

Figure S67. Mass spectrum of metabolite **8** 34

Figure S68. ^1H NMR spectrum of metabolite **8** 35

Figure S69. ^1H NMR (0.8-2.8) spectrum of metabolite **8** 35

Figure S70. ^{13}C NMR spectrum of metabolite **8** 36

Figure S71. COSY (^1H-^1H) NMR spectrum of metabolite **8** 36

Figure S72. HSQC (^1H-^{13}C) NMR spectrum of metabolite **8** 37

Figure S73. HMBC (long distance correlation ^1H-^{13}C) NMR spectrum of metabolite **8** 37

9. Metabolite 9: 4,2''-dihydroxycannabidiol 38

Figure S74. UHPLC-DAD chromatogram of metabolite **9** 38

Figure S75. UV spectrum of metabolite **9** 38

Figure S76. Mass spectrum of metabolite **9** 38

Figure S77. ^1H NMR spectrum of metabolite **9** 39

Figure S78. ^1H NMR (0.7-2.6 ppm) spectrum of metabolite **9** 39

Figure S79. ^{13}C NMR spectrum of metabolite **9** 40

Figure S80. COSY (^1H-^1H) NMR spectrum of metabolite **9** 40

Figure S81. HSQC (1H-13C) NMR spectrum of metabolite **9** 41

Figure S82. HMBC (long distance correlation ^1H-^{13}C) NMR spectrum of metabolite **9** 41

10. Statistical analysis 42

Table 1. One-way ANOVA – Time significance 42

Table 2. One-way ANOVA and T-Student test – Metabolite **2** over time – *I. fumosorosea* KCh J2 43

Table 3. One-way ANOVA and T-Student test – Metabolite **8** over time - *I. fumosorosea* KCh J2 44

Table 4. One-way ANOVA and T-Student test – Metabolite **7** over time - *I. fumosorosea* KCh J2 45

Table 5. T-Student test – *M. hiemalis* strains 46

1. Cannabidiol (1)

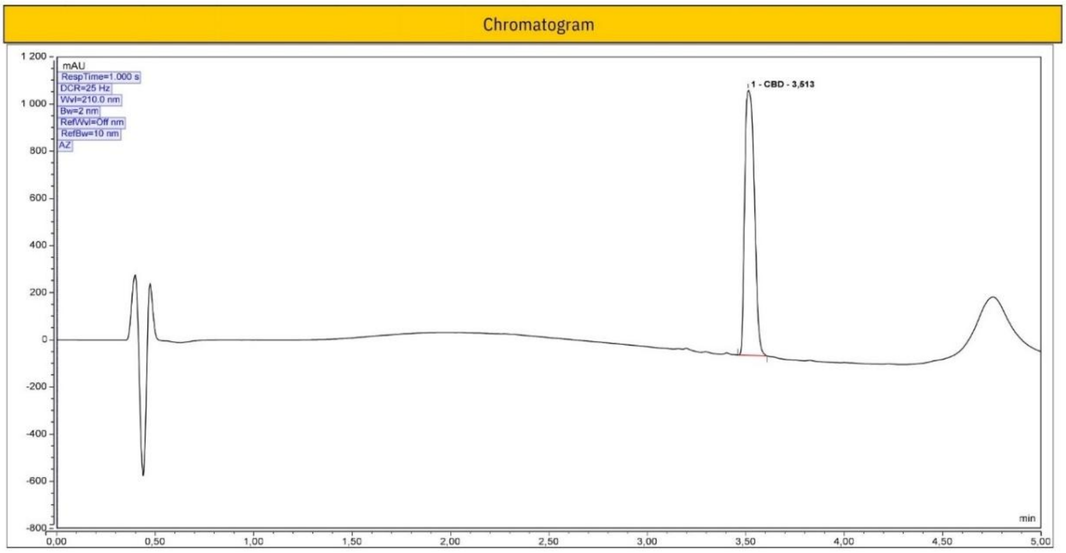

Figure S1. UHPLC-DAD chromatogram of substrate (CBD) (**1**)

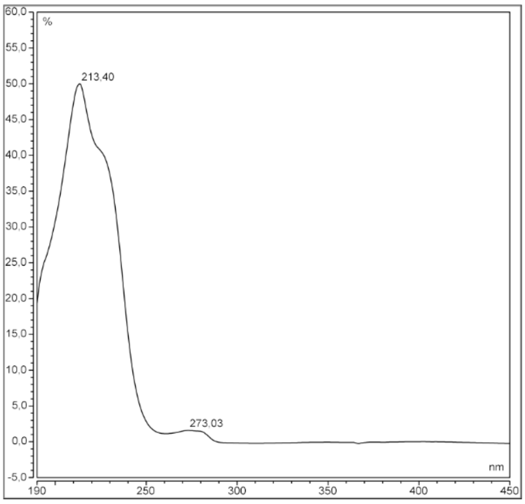

Figure S2. UV spectrum of substrate (CBD) (**1**)

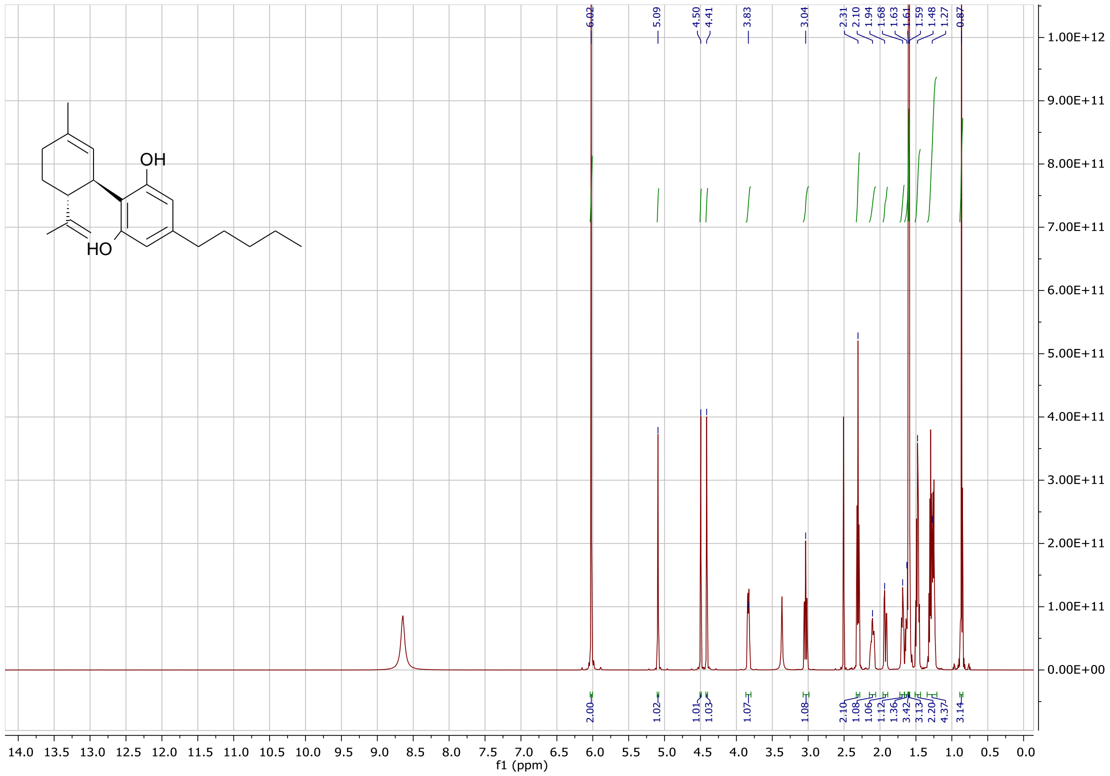

Figure S3. ^{1}H NMR spectrum of cannabidiol **1**

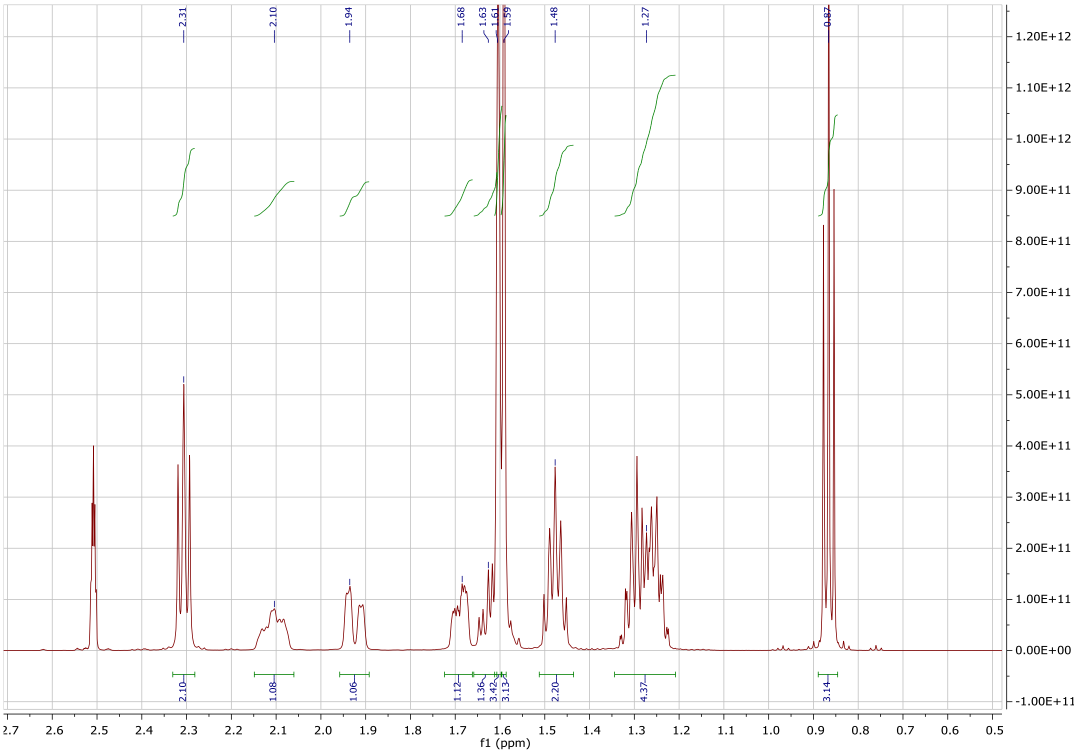

Figure S4. ^{1}H NMR (0.5-2.5ppm) spectrum of cannabidiol **1**

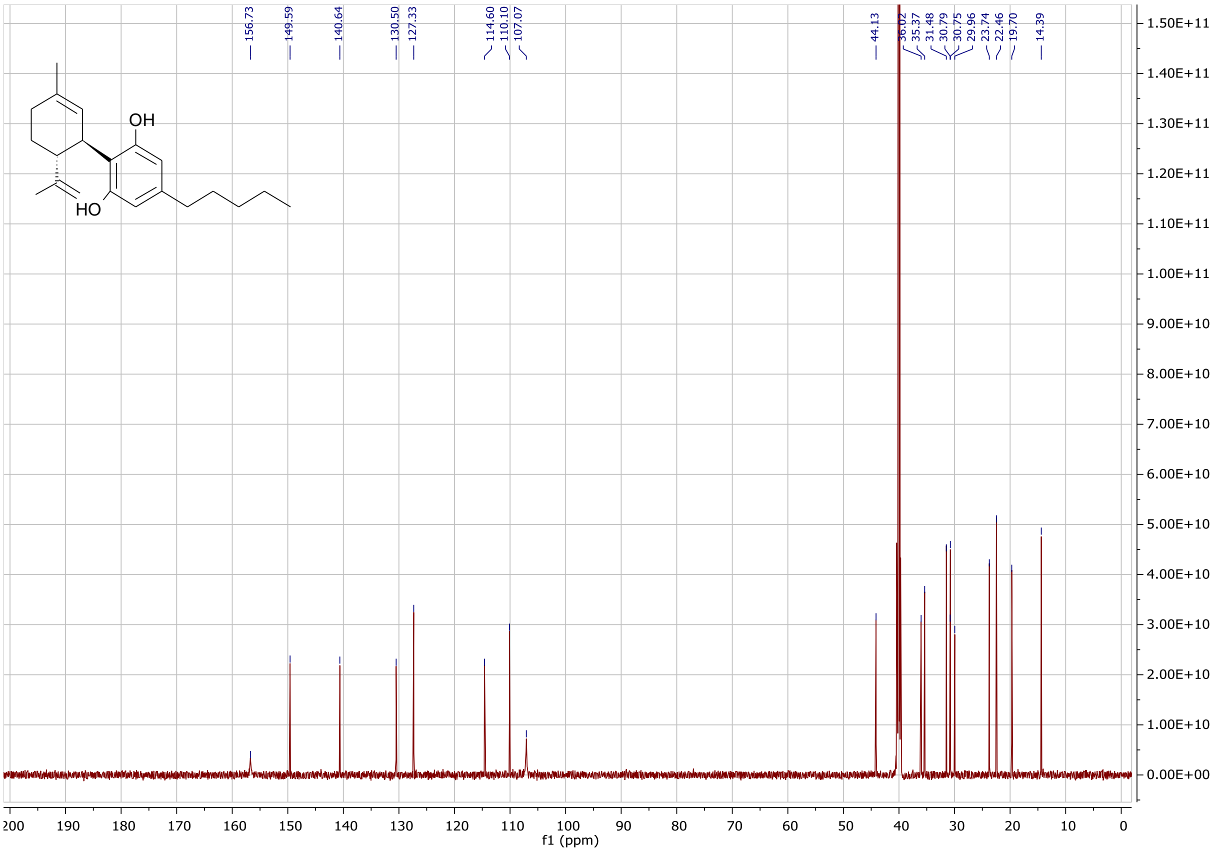

Figure S5. ^{13}C NMR spectrum of cannabidiol **1**

2. Metabolite 2: 2'-O-(β-D-glucopyranosyl)-cannabidiol

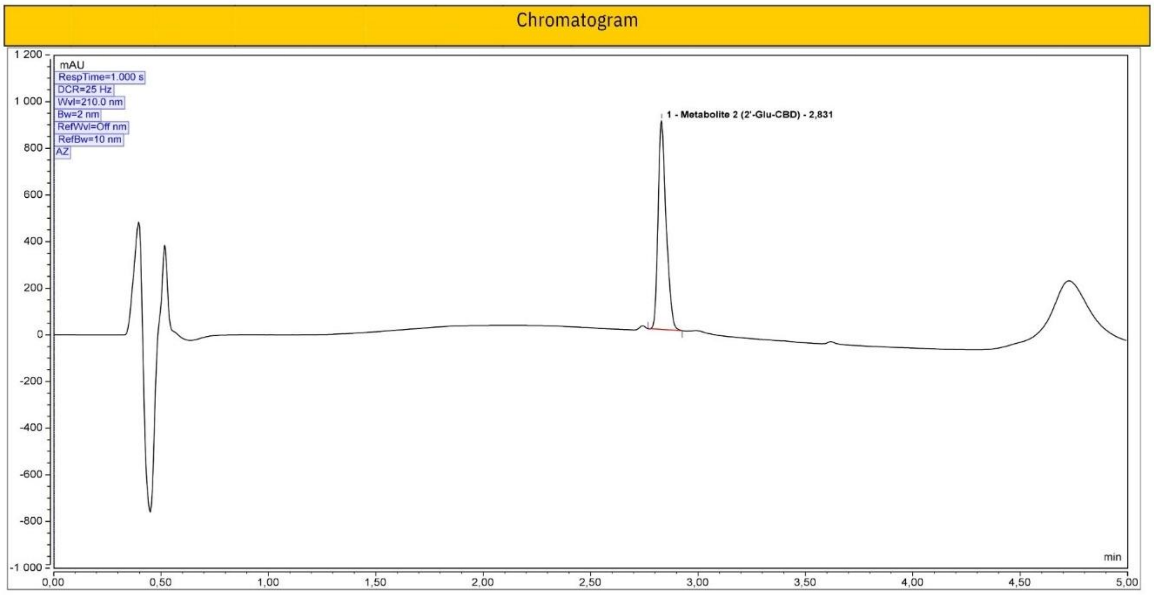

Figure S6. UHPLC-DAD chromatogram of metabolite **2**

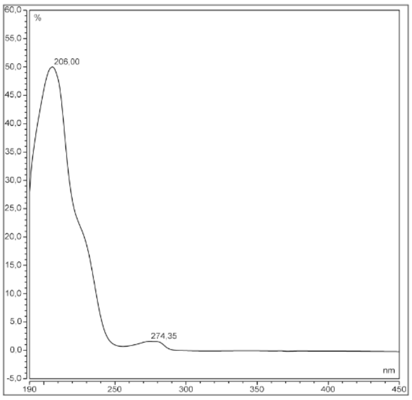

Figure S7. UV spectrum of metabolite **2**

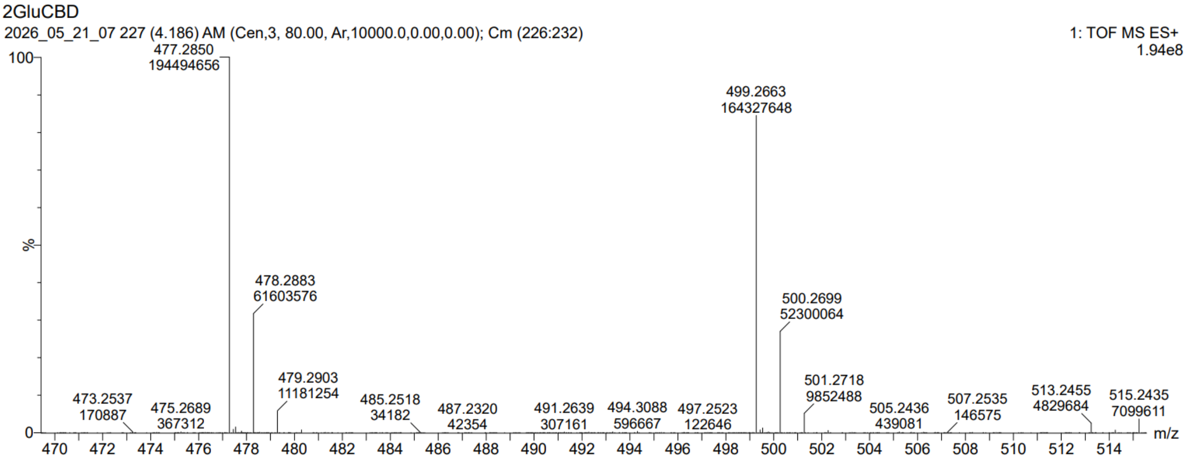

Figure S8. Mass spectrum of metabolite **2**

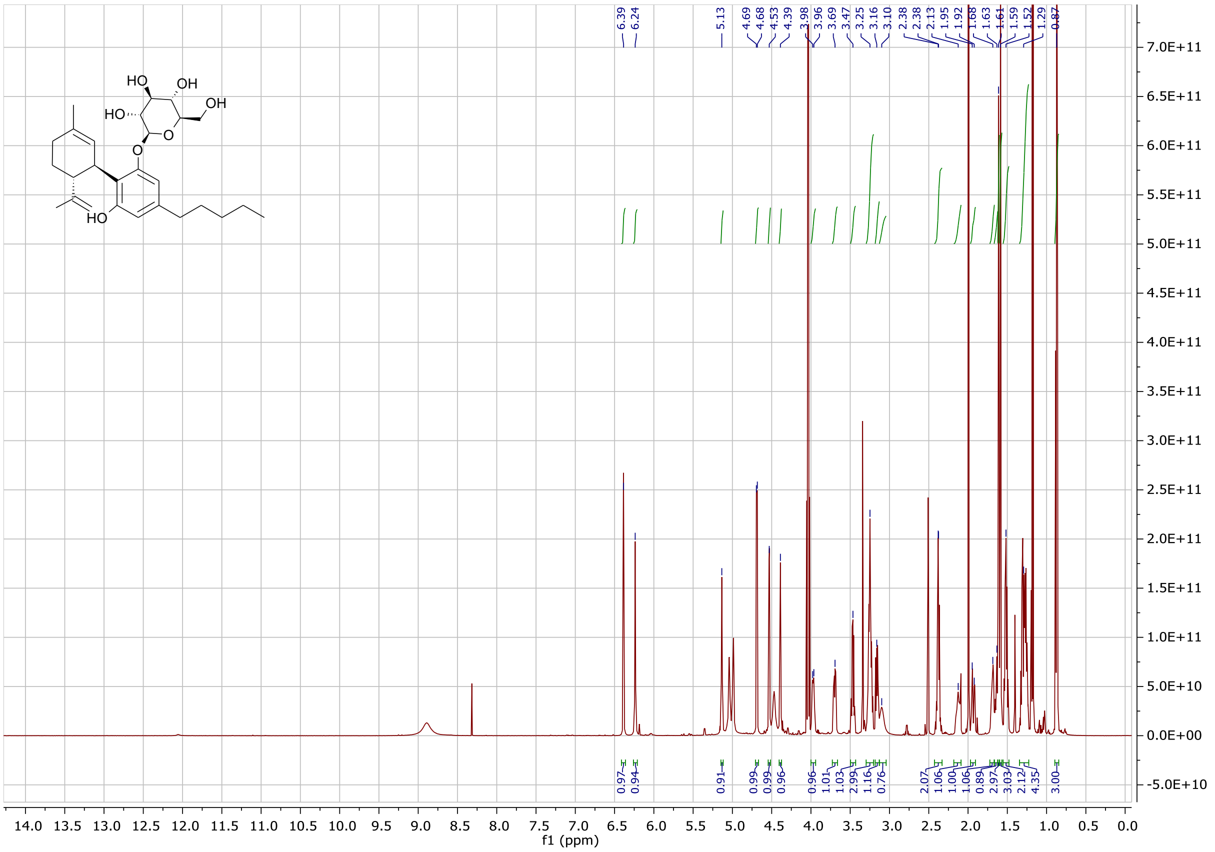

Figure S9. ^{1}H NMR spectrum of metabolite **2**

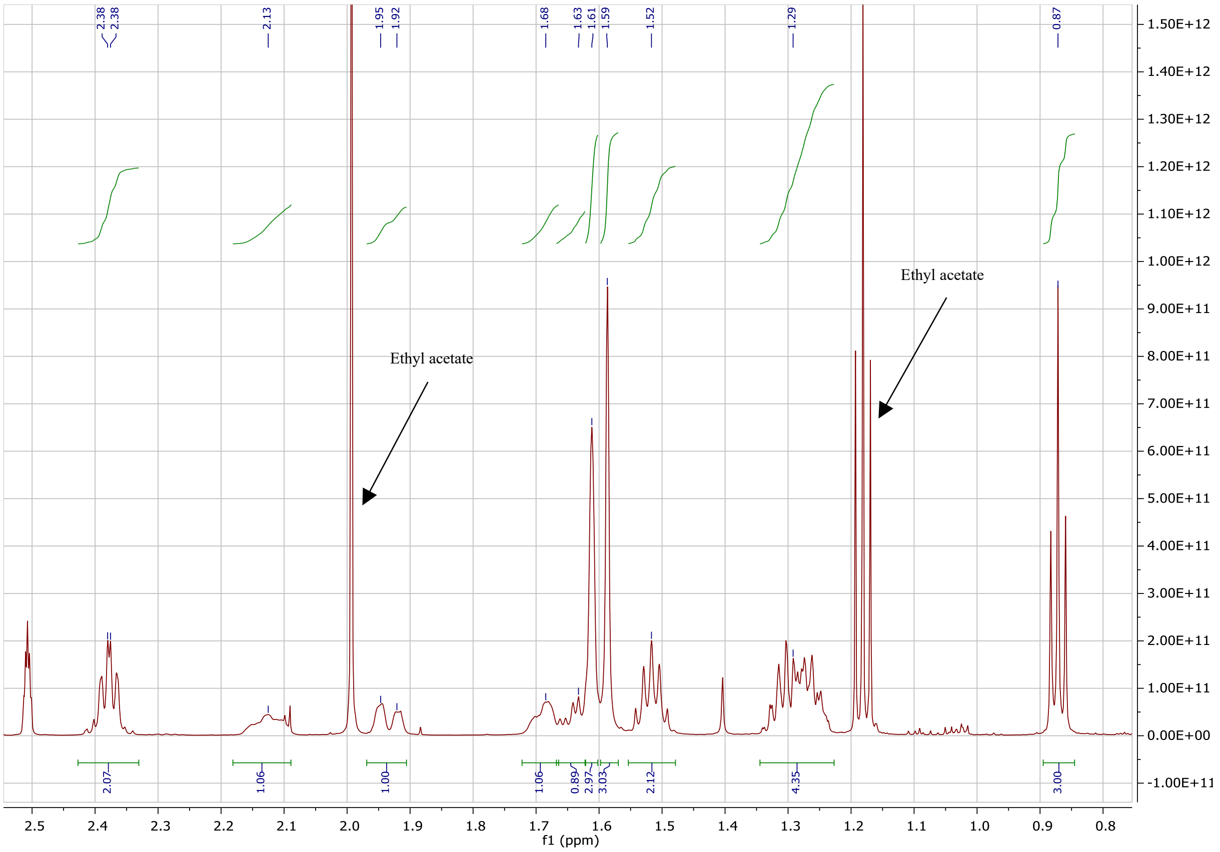

Figure S10. ^{1}H NMR spectrum of metabolite **2** (0.8-2.5ppm)

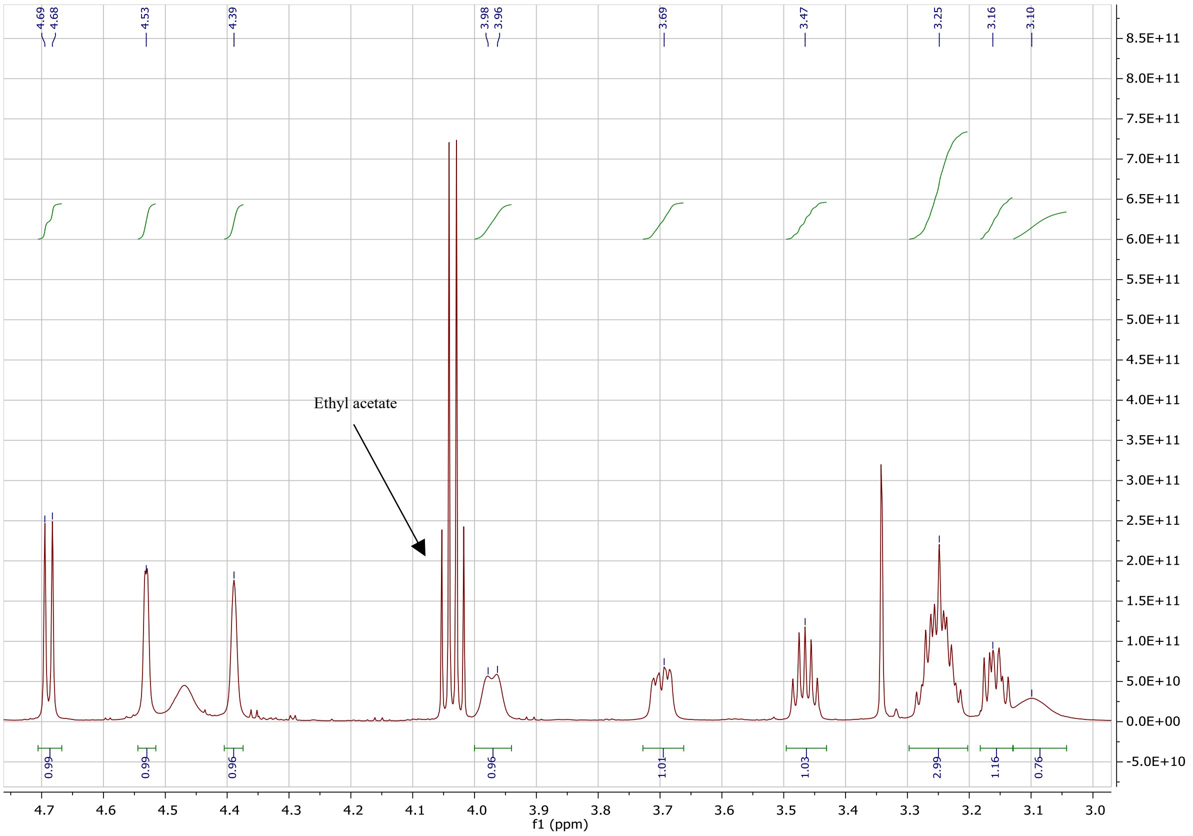

Figure S11. ^{1}H NMR spectrum of metabolite **2** (2.6-6.4 ppm)

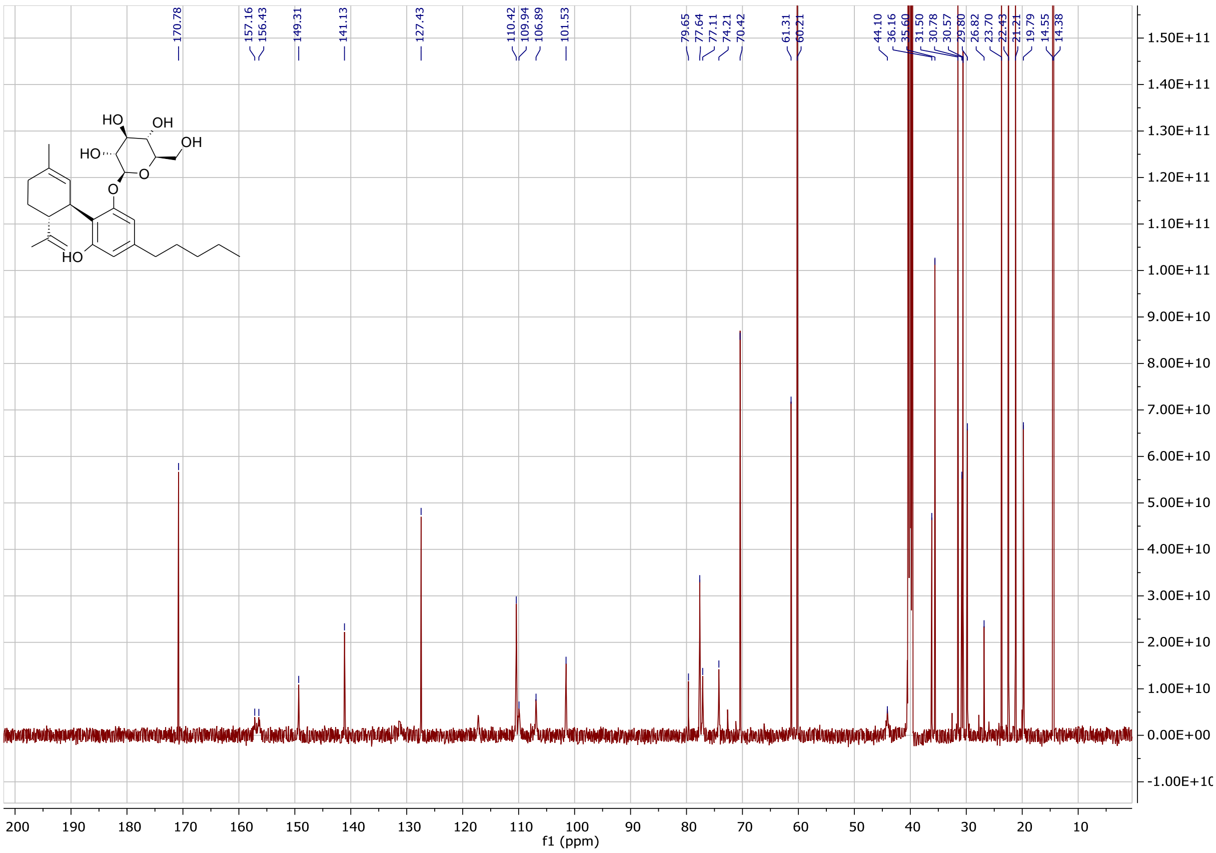

Figure S12. ^{13}C NMR spectrum of metabolite **2**

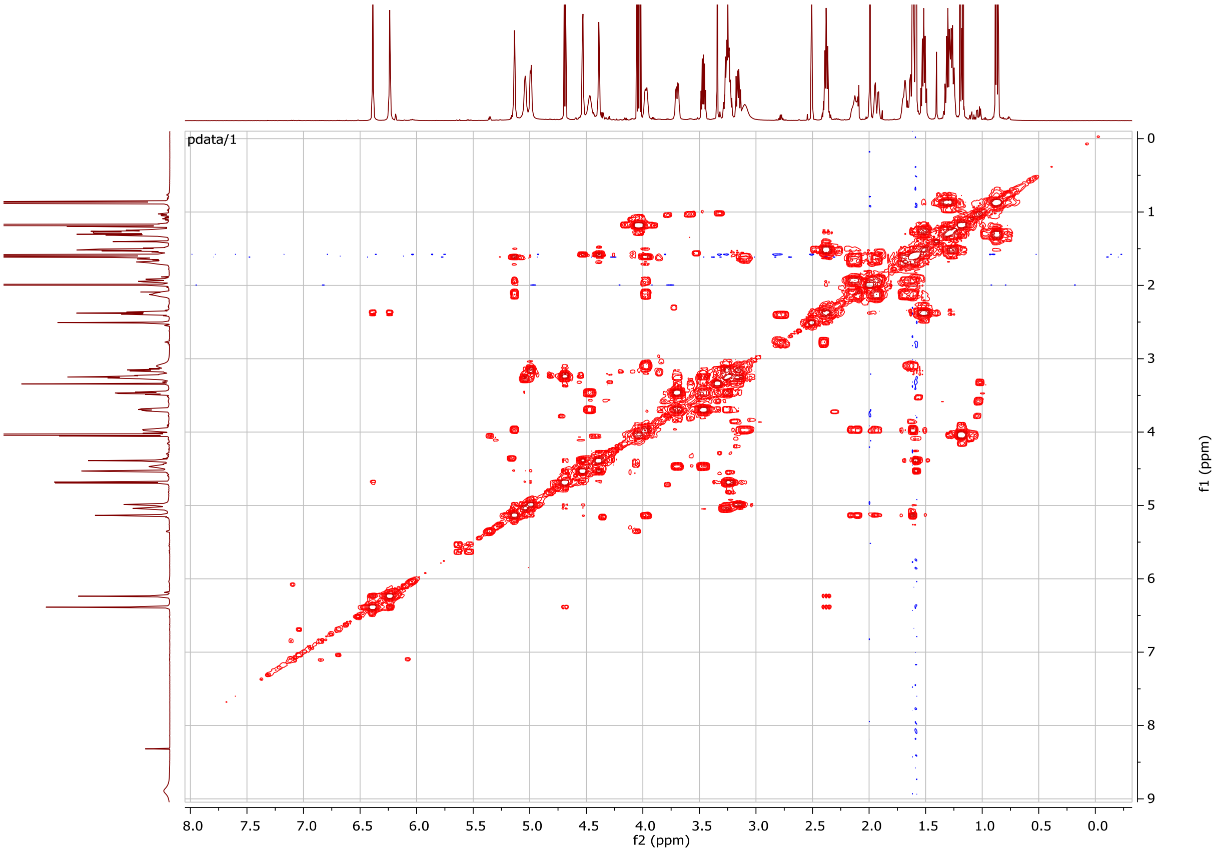

Figure S13. COSY (^{1}H-^{1}H) NMR spectrum of metabolite **2**

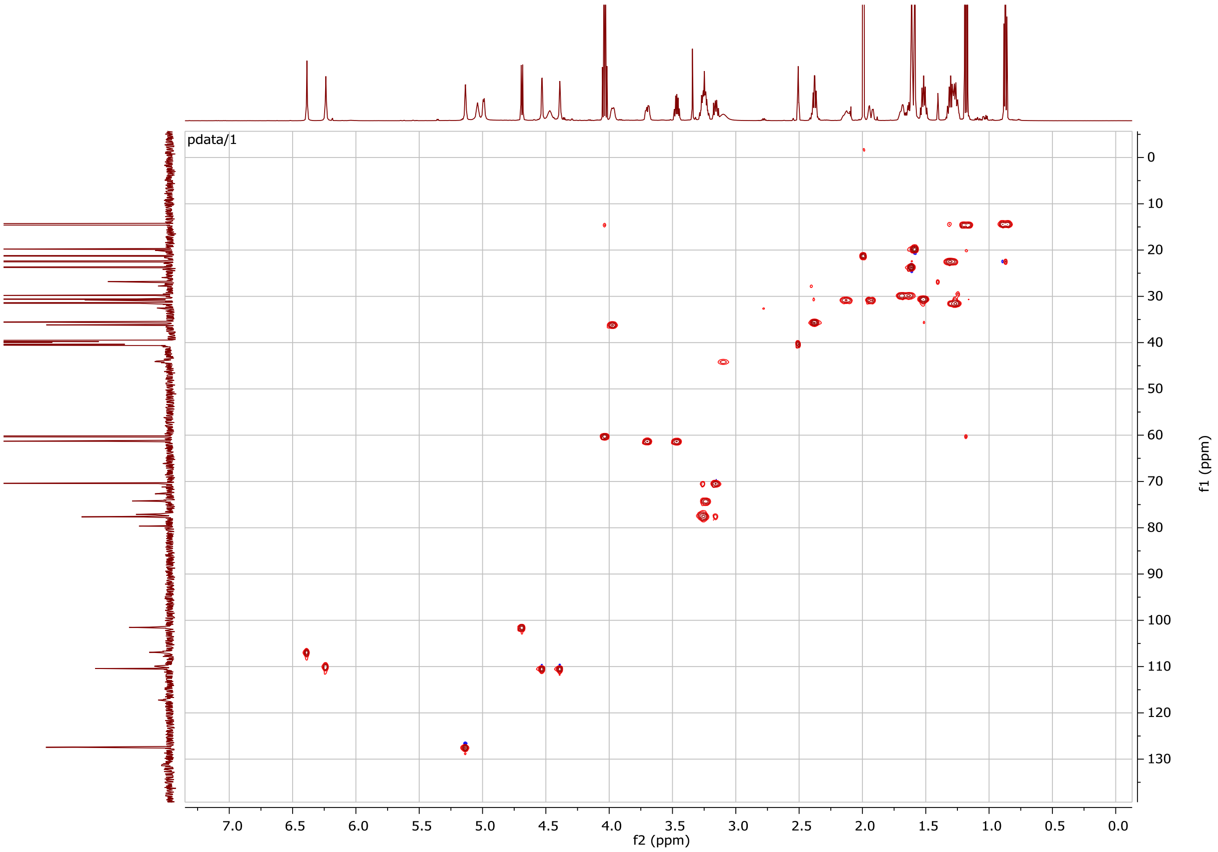

Figure S14. HSQC (1H-13C) NMR spectrum of metabolite **2**

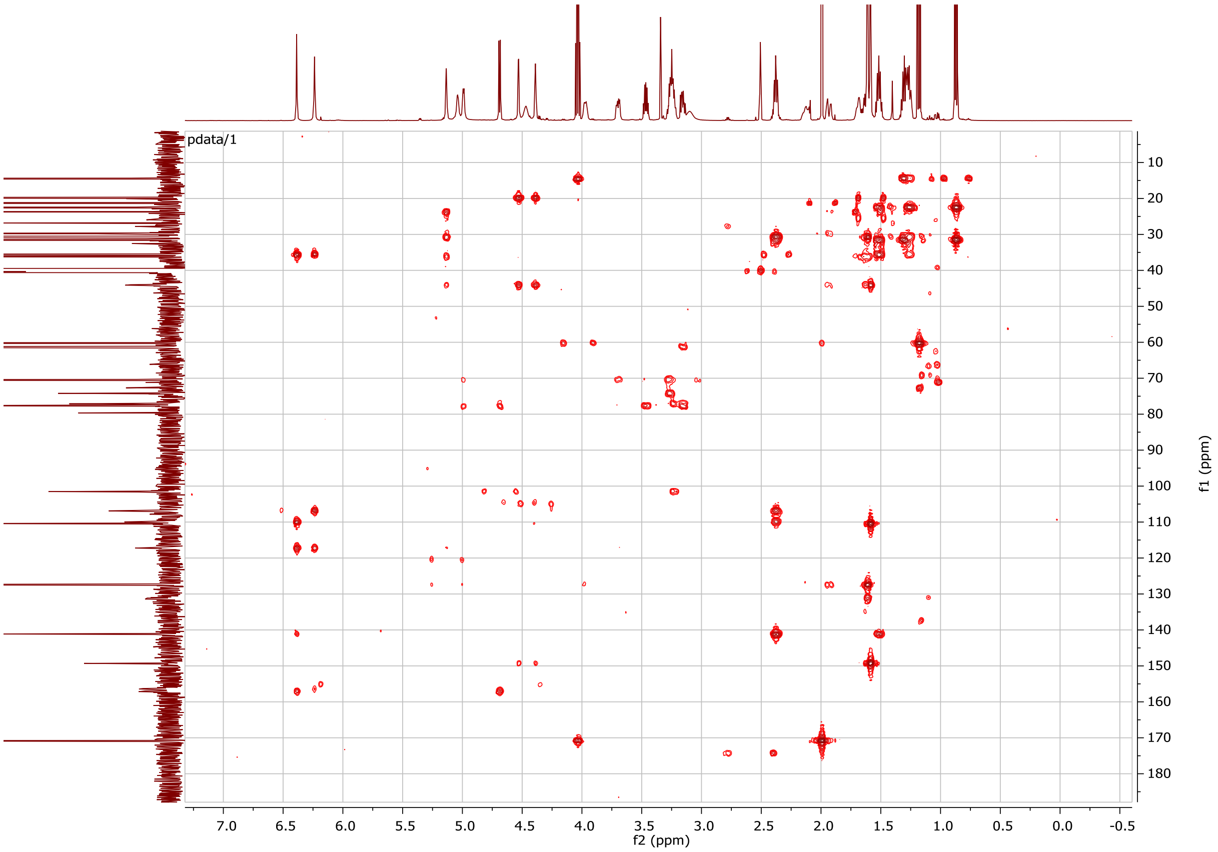

Figure S15. HMBC (long distance correlation ^{1}H-^{13}C) spectrum of metabolite **2**

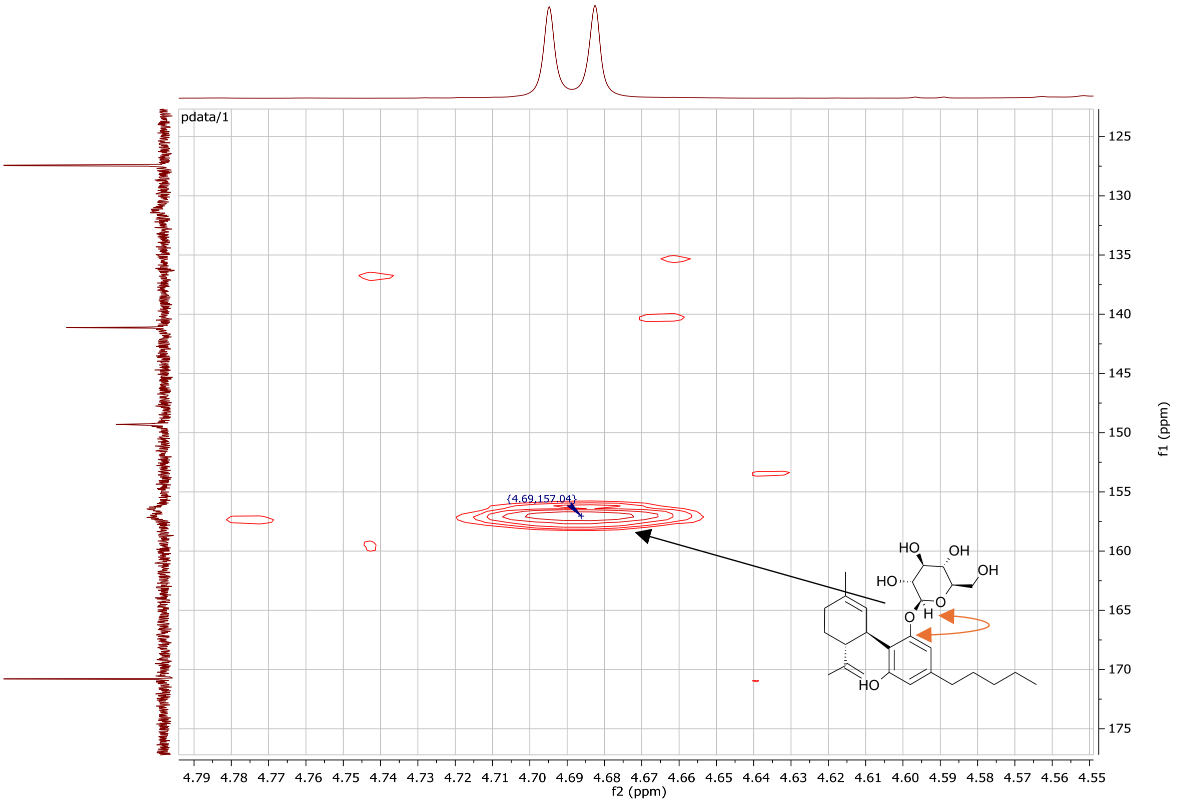

Figure S16. HMBC correlation between C1 carbon of glucose and C2 carbon of aromatic ring.

3. Metabolite 3: 2'-O-(β-D-glucopyranosyl)-4"-hydroxycannabidiol

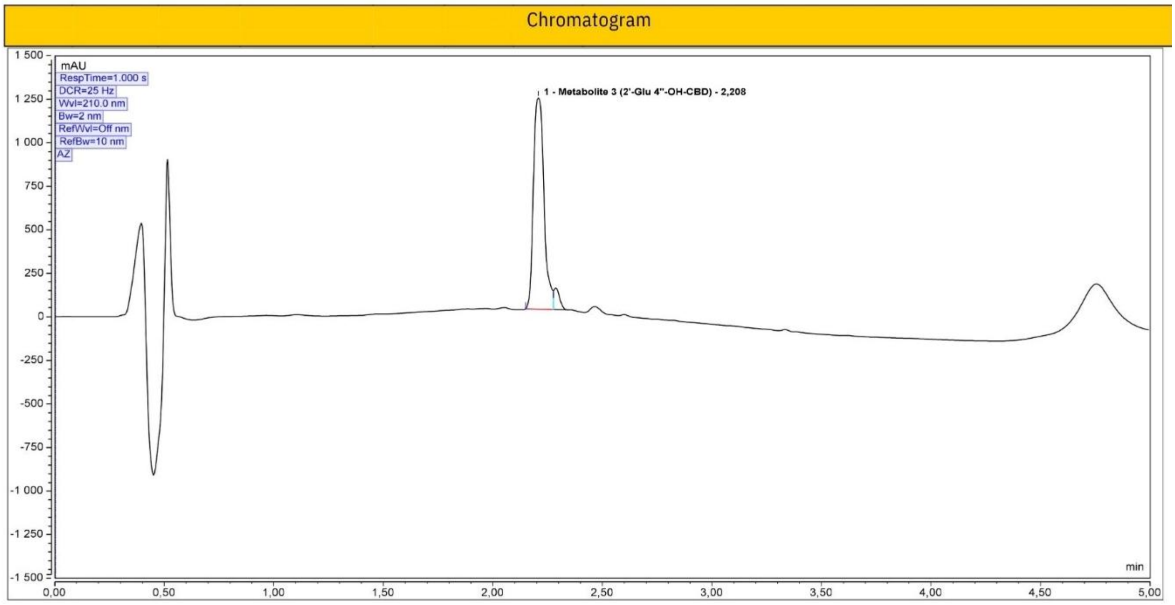

Figure S17. UHPLC-DAD chromatogram of metabolite **3**

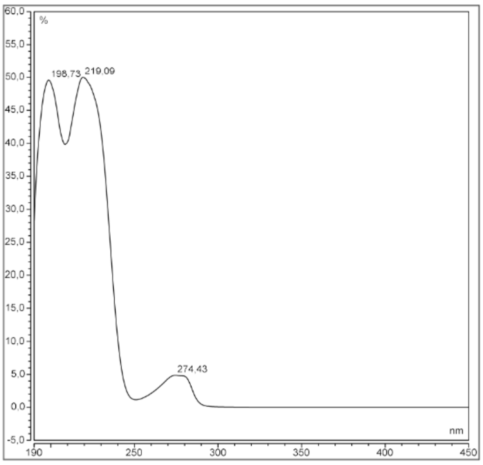

Figure S18. UV spectrum of metabolite **3**

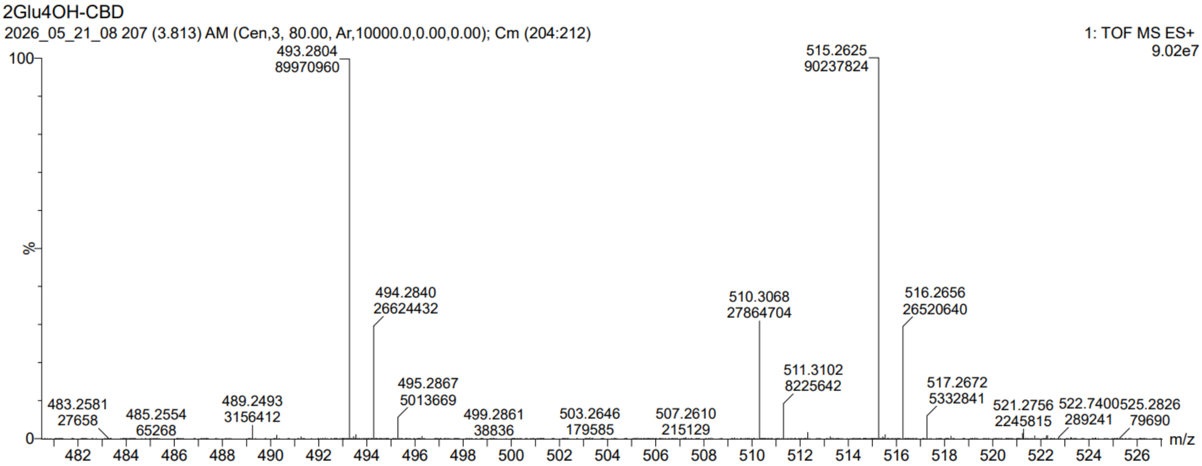

Figure S19. Mass spectrum of metabolite **3**

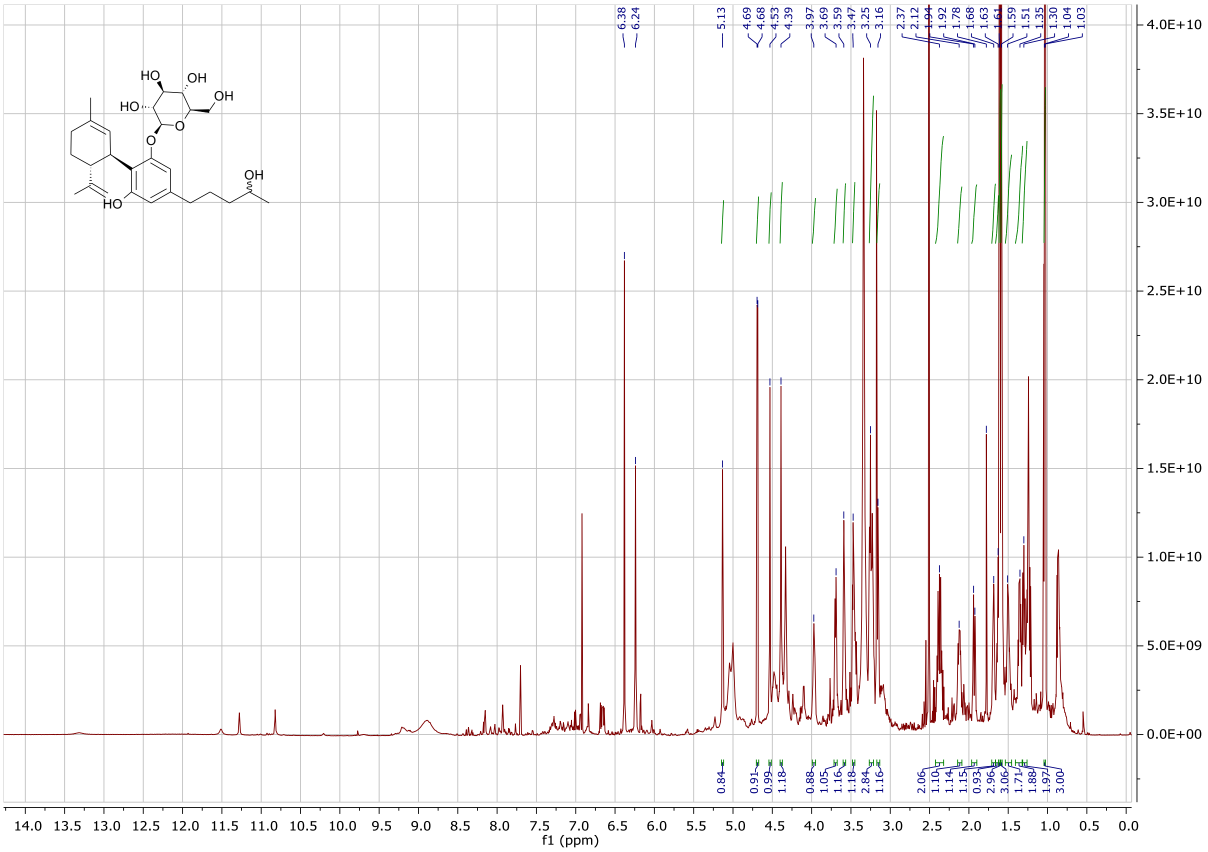

Figure S20. ^{1}H NMR spectrum of metabolite **3**

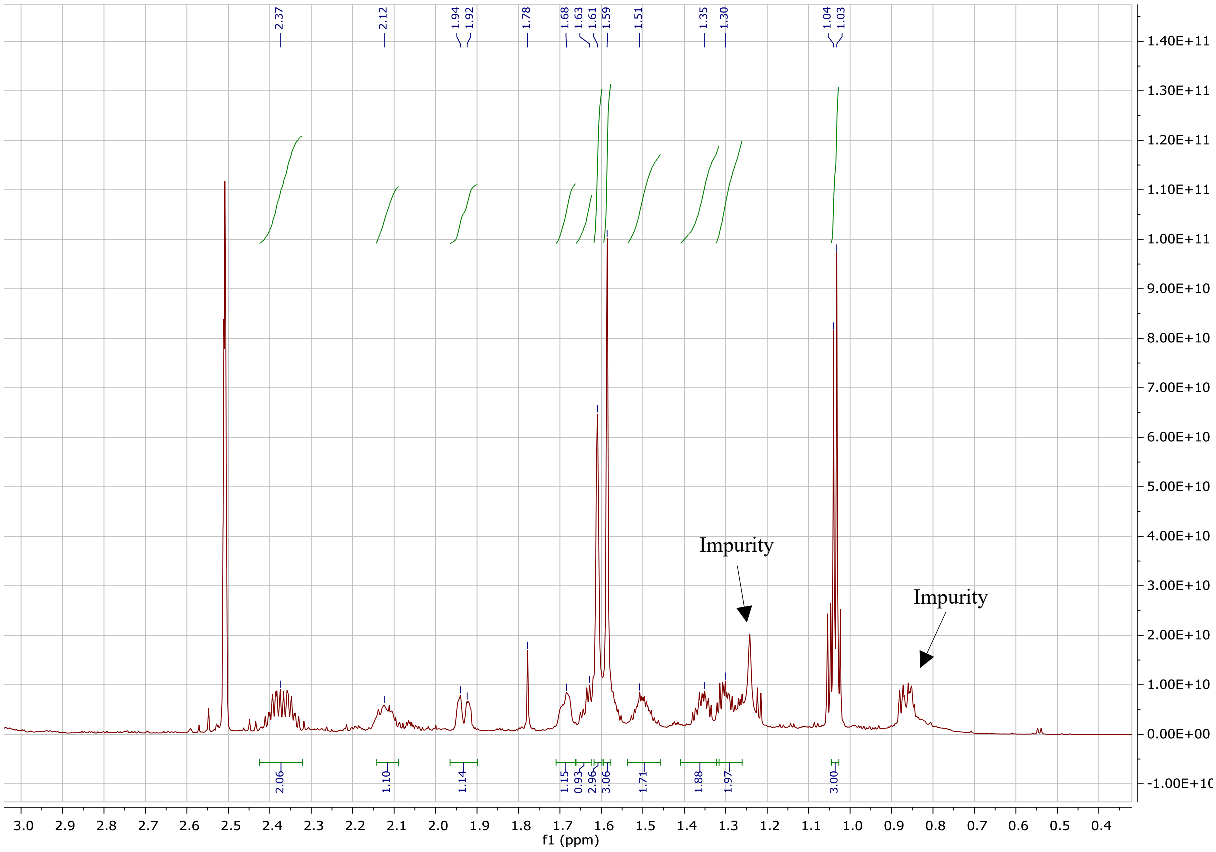

Figure S21. ^{1}H NMR (0.4-3.0ppm) spectrum of metabolite **3**

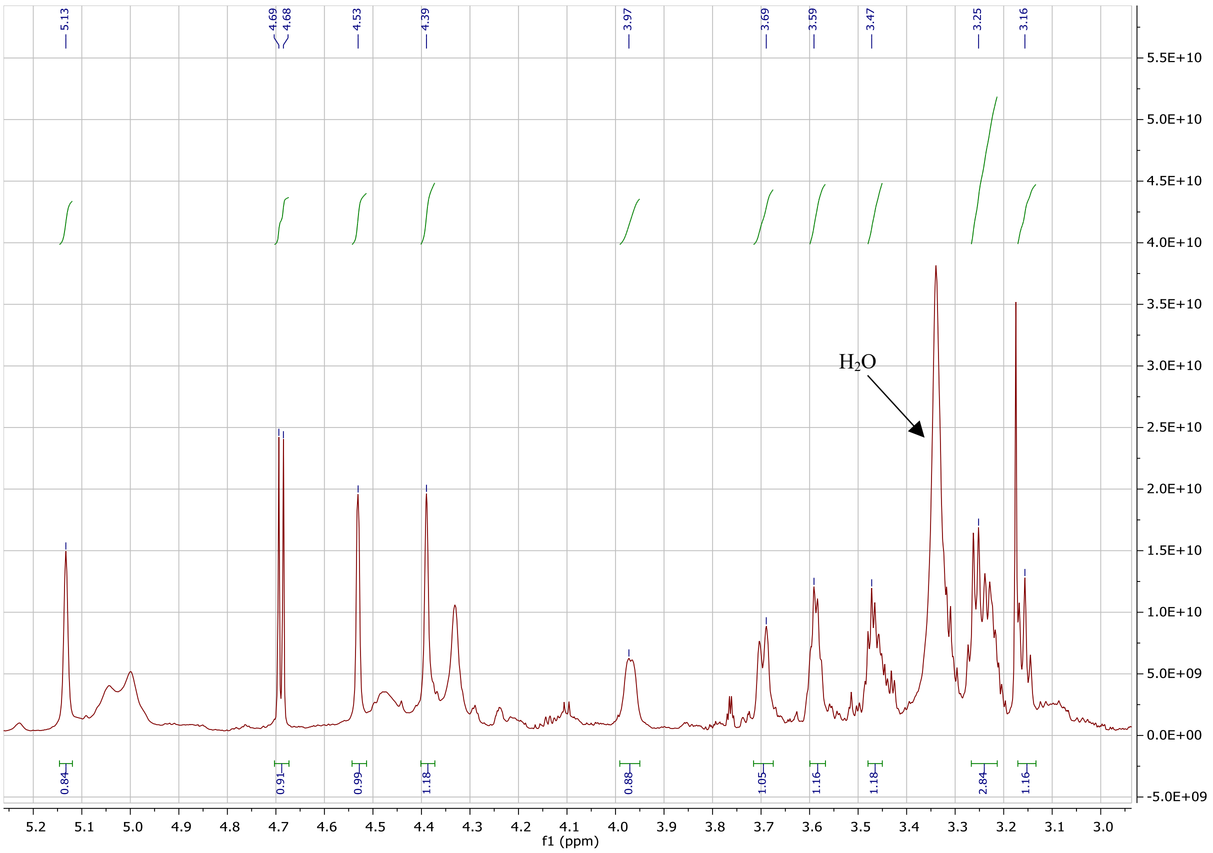

Figure S22. ^{1}H NMR spectrum of metabolite **3** (3.0-5.2 ppm)

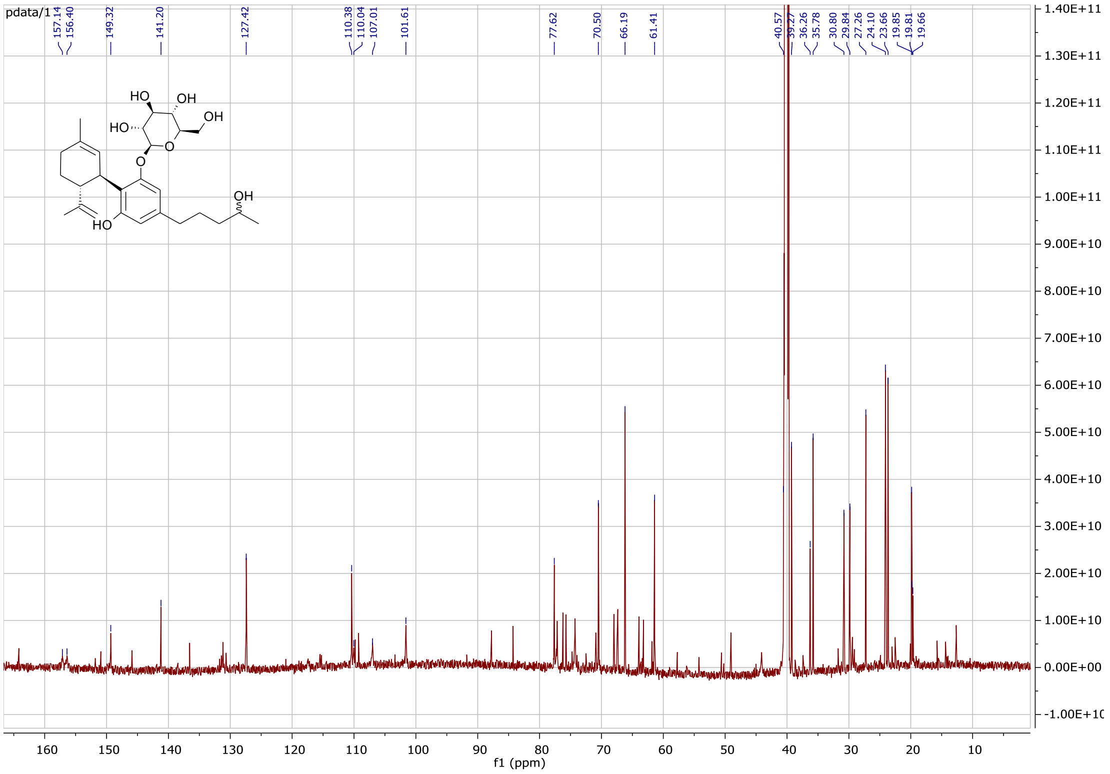

Figure S23. 13C NMR spectrum of metabolite **3**

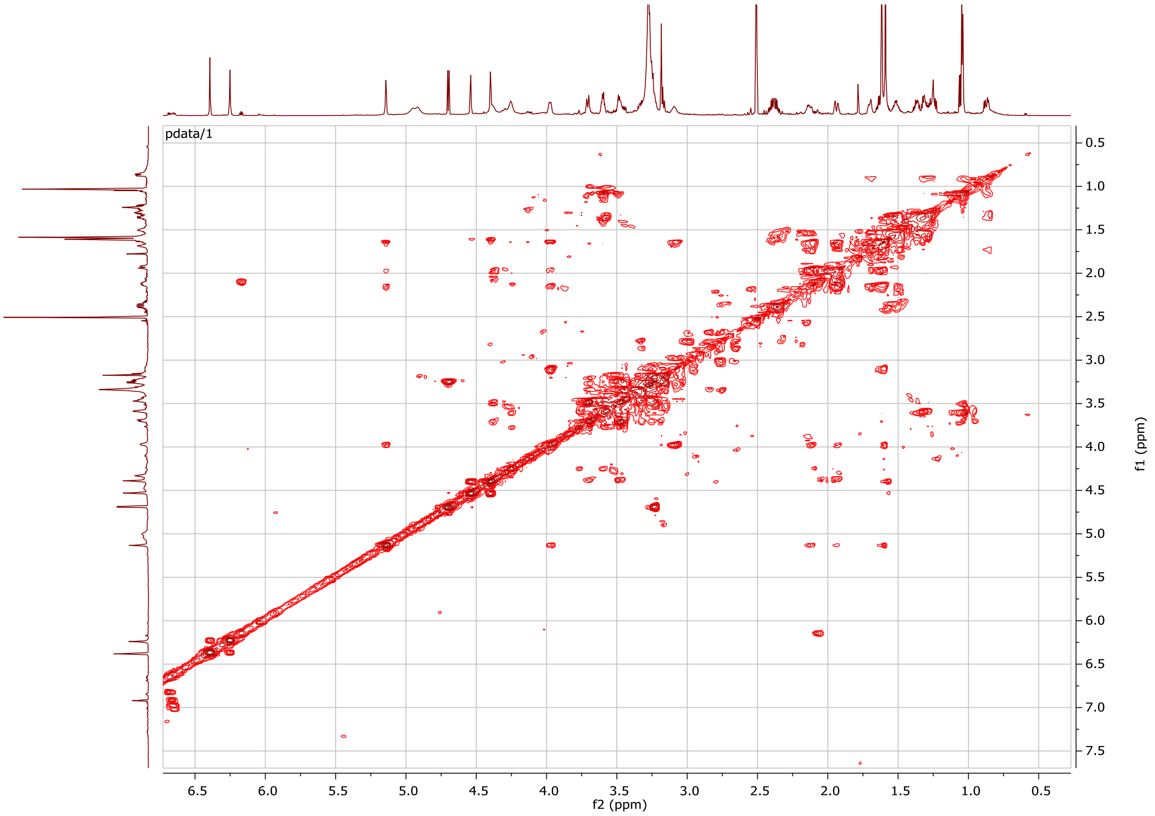

Figure S24. COSY (^{1}H-^{1}H) NMR spectrum of metabolite **3**

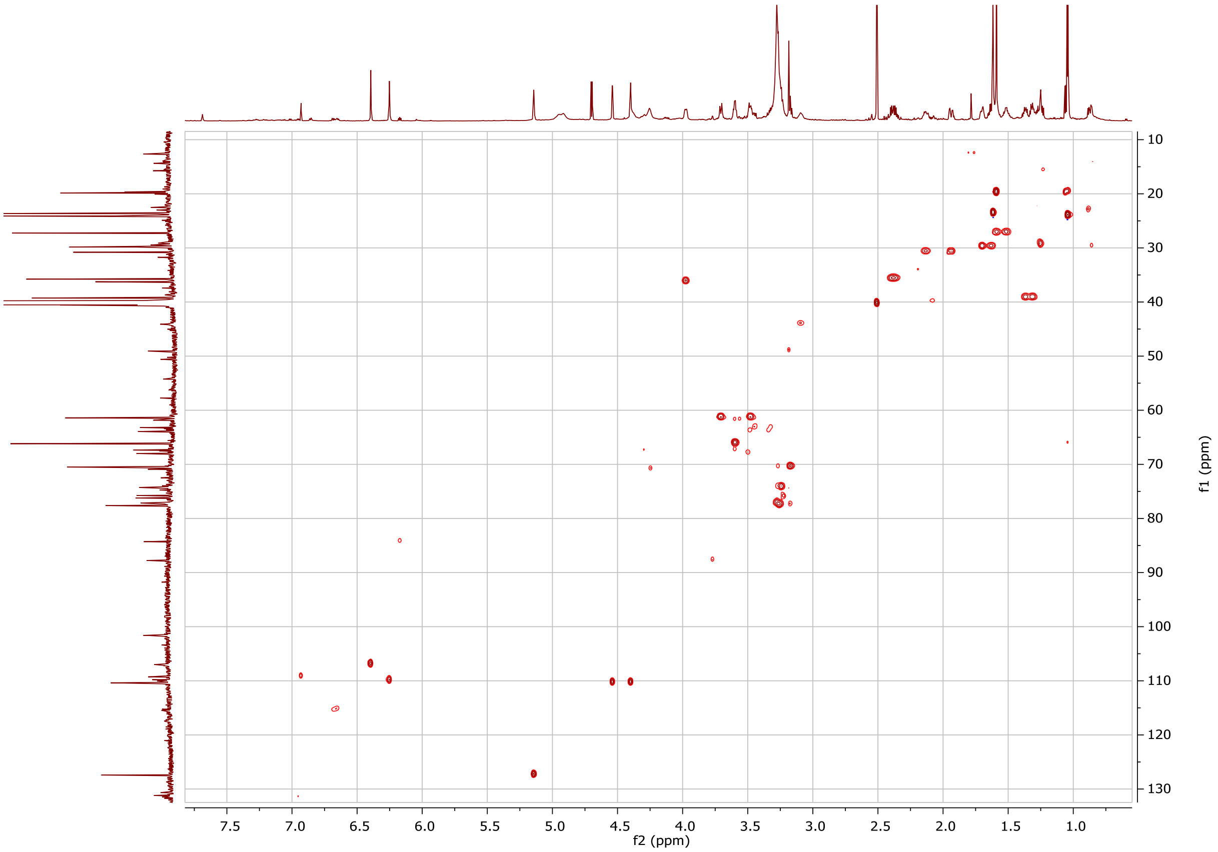

Figure S25. HSQC (^1H-^{13}C) NMR spectrum of metabolite **3**

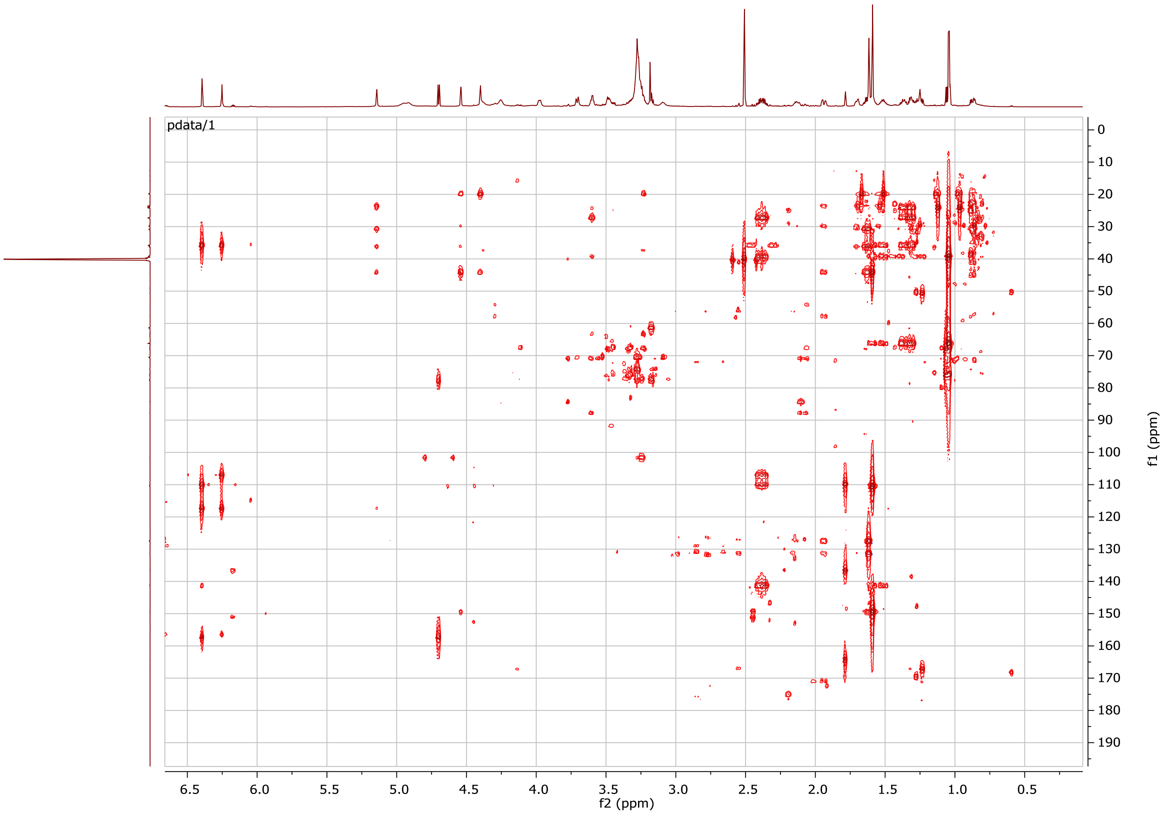

Figure S26. HMBC (long distance correlation ^1H-^{13}C) spectrum of metabolite **3**

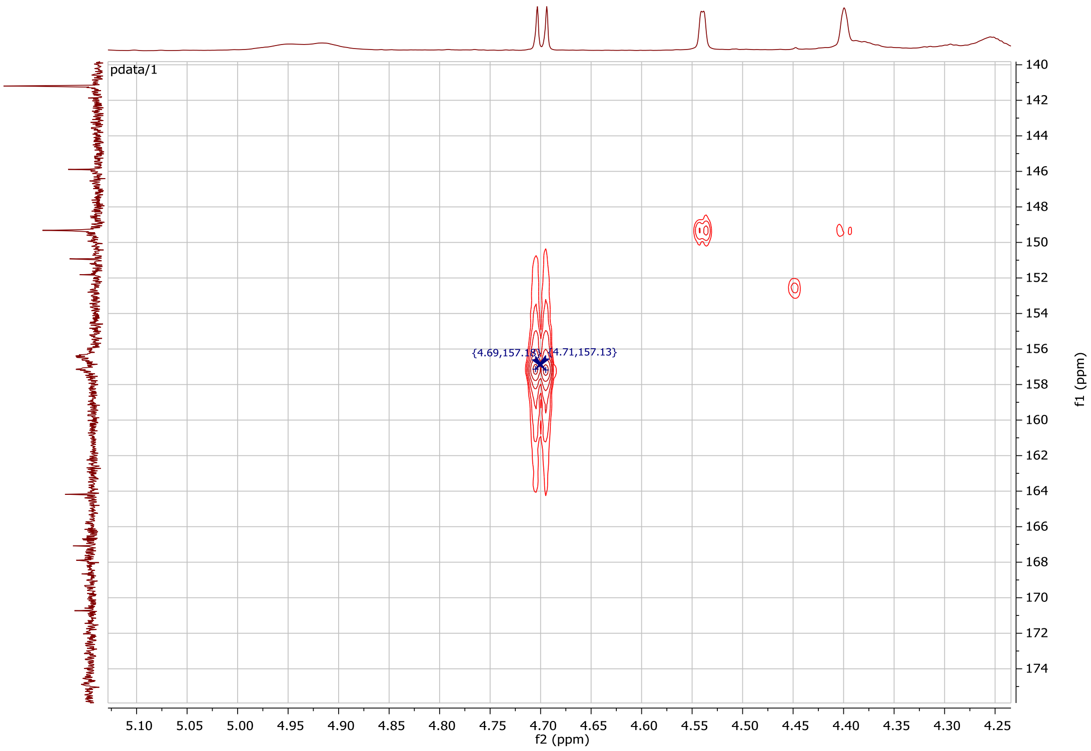

Figure S27. HMBC correlation between C1 carbon of glucose and C2 carbon of aromatic ring in metabolite **3**

4. Metabolite 4: 4”-hydroxycannabidiol

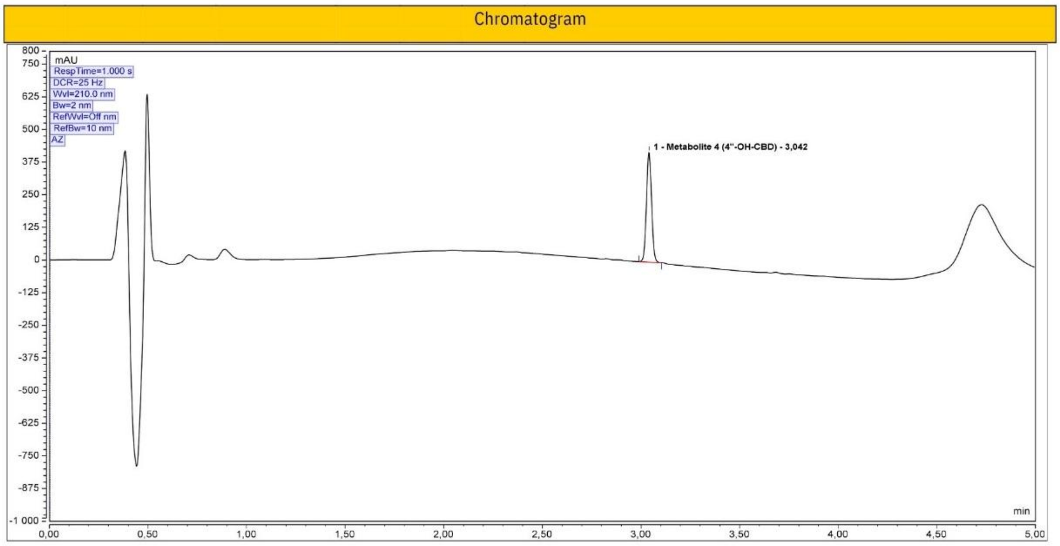

Figure S28. UHPLC-DAD chromatogram of metabolite **4**

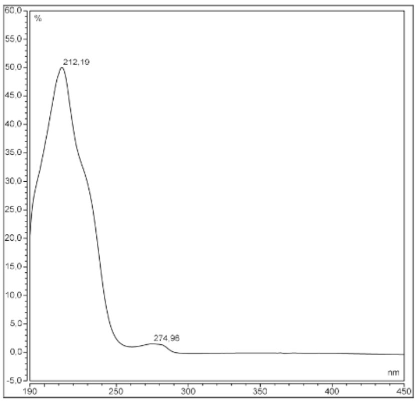

Figure S29. UV spectrum of metabolite **4**

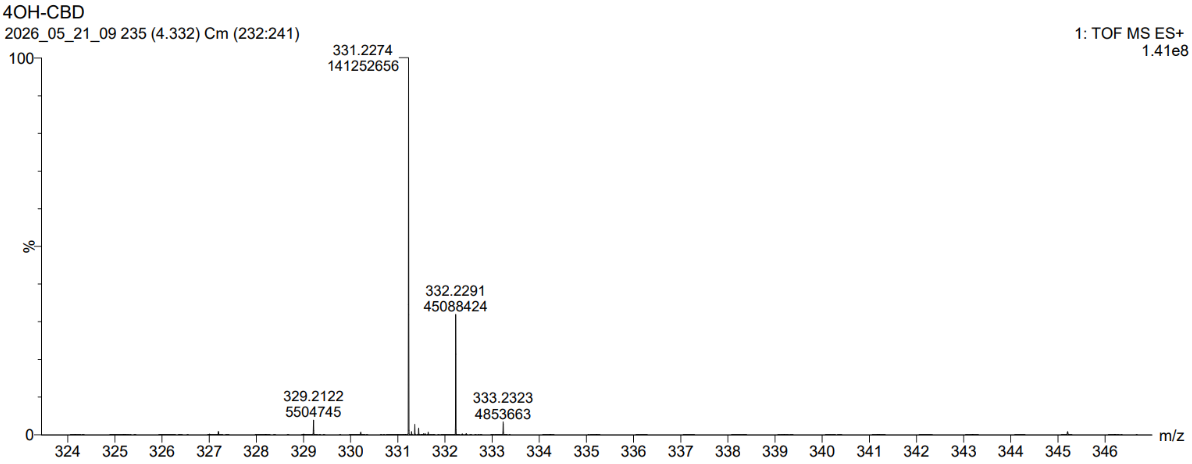

Figure S30. Mass spectrum of metabolite **4**

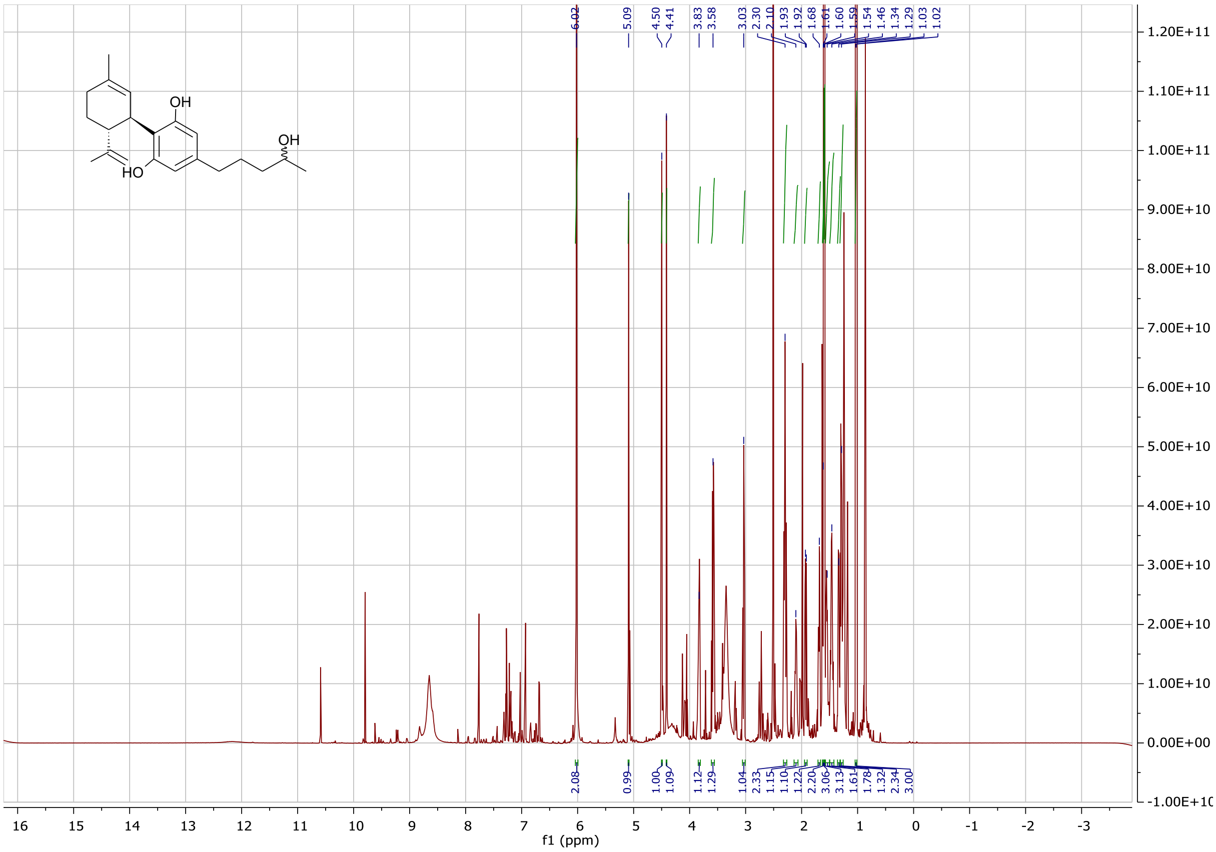

Figure S31. ^{1}H NMR spectrum of metabolite **4**

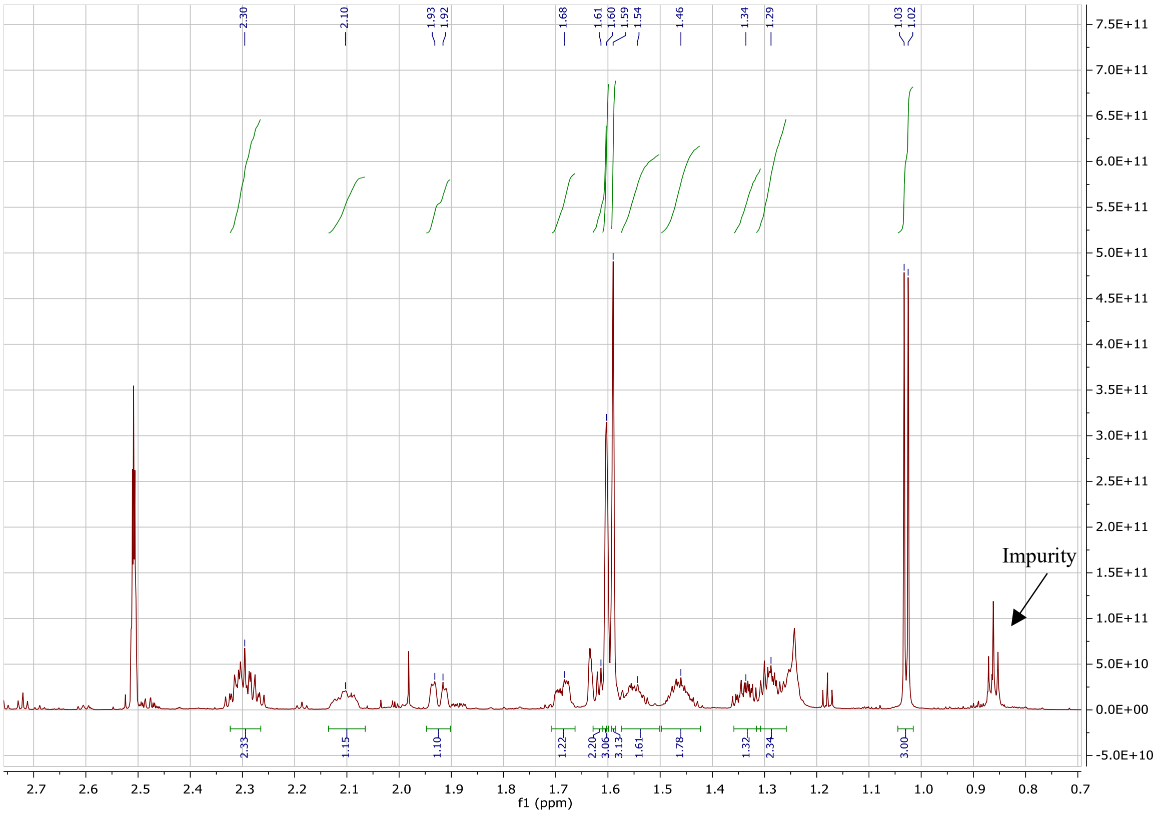

Figure S32. ^{1}H NMR (0.7-2.5) spectrum of metabolite **4**

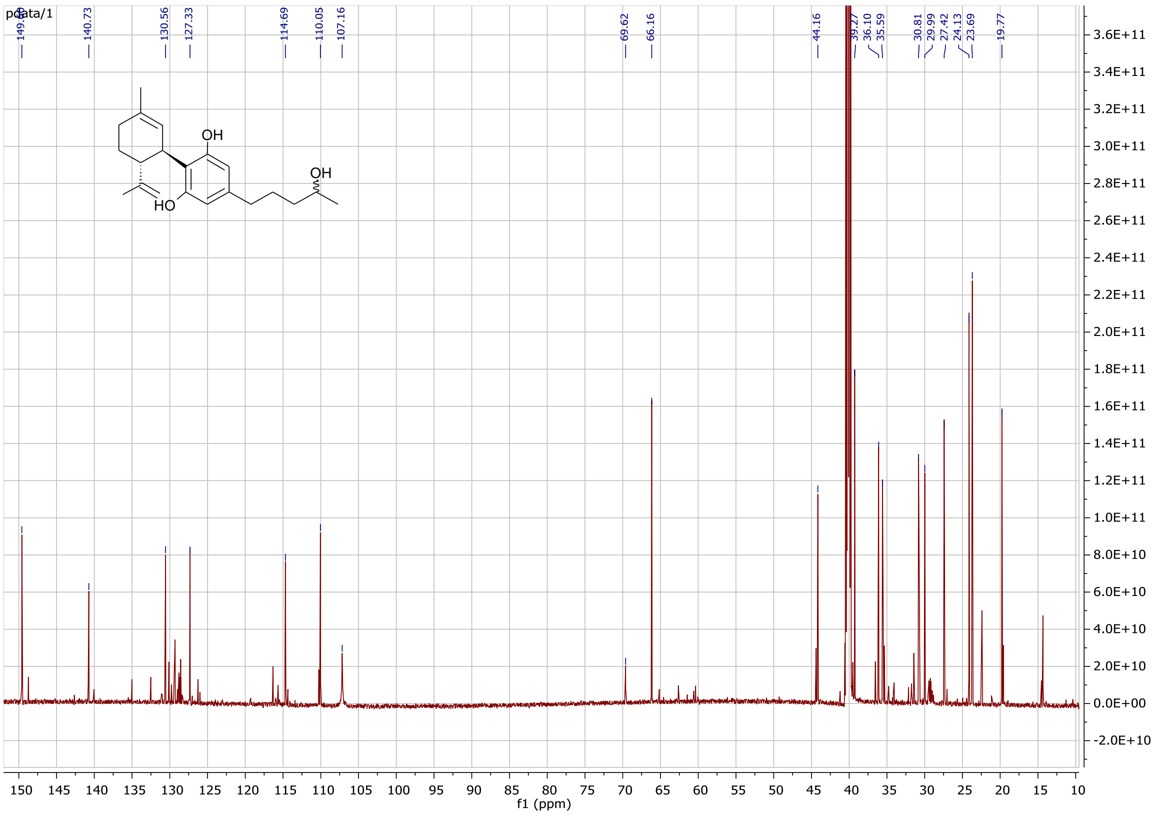

Figure S33. 13C NMR spectrum of metabolite **4**

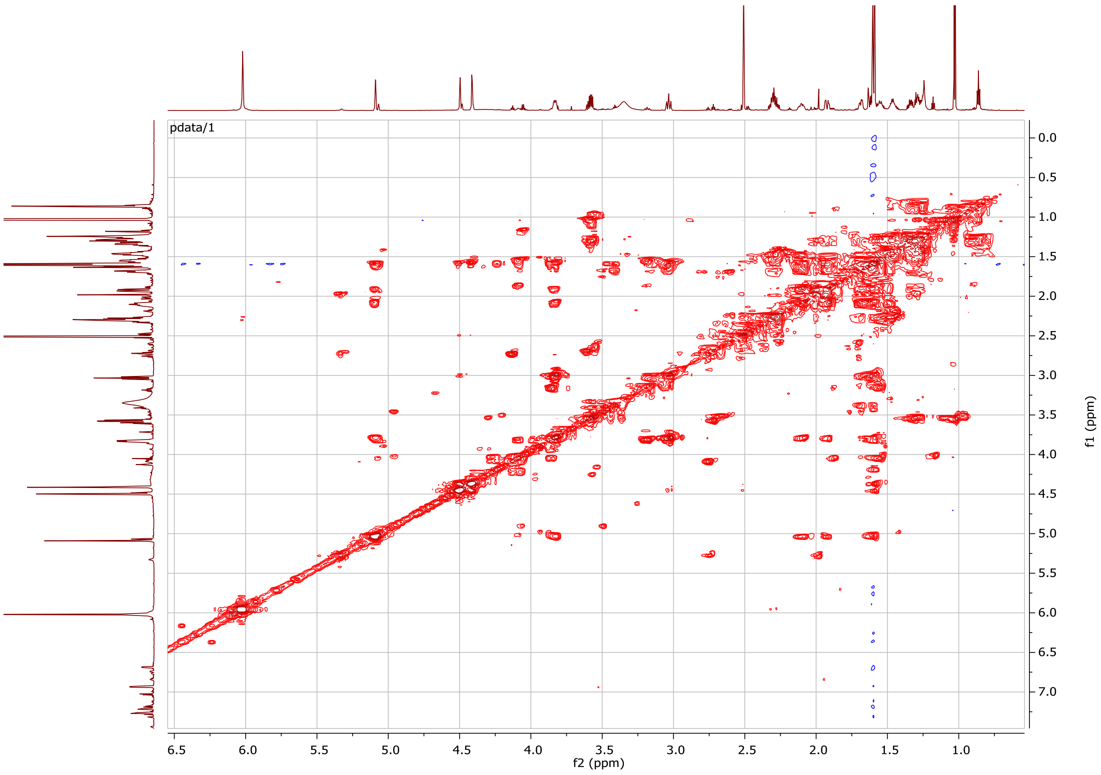

Figure S34. COSY (^{1}H-^{1}H) NMR spectrum of metabolite **4**

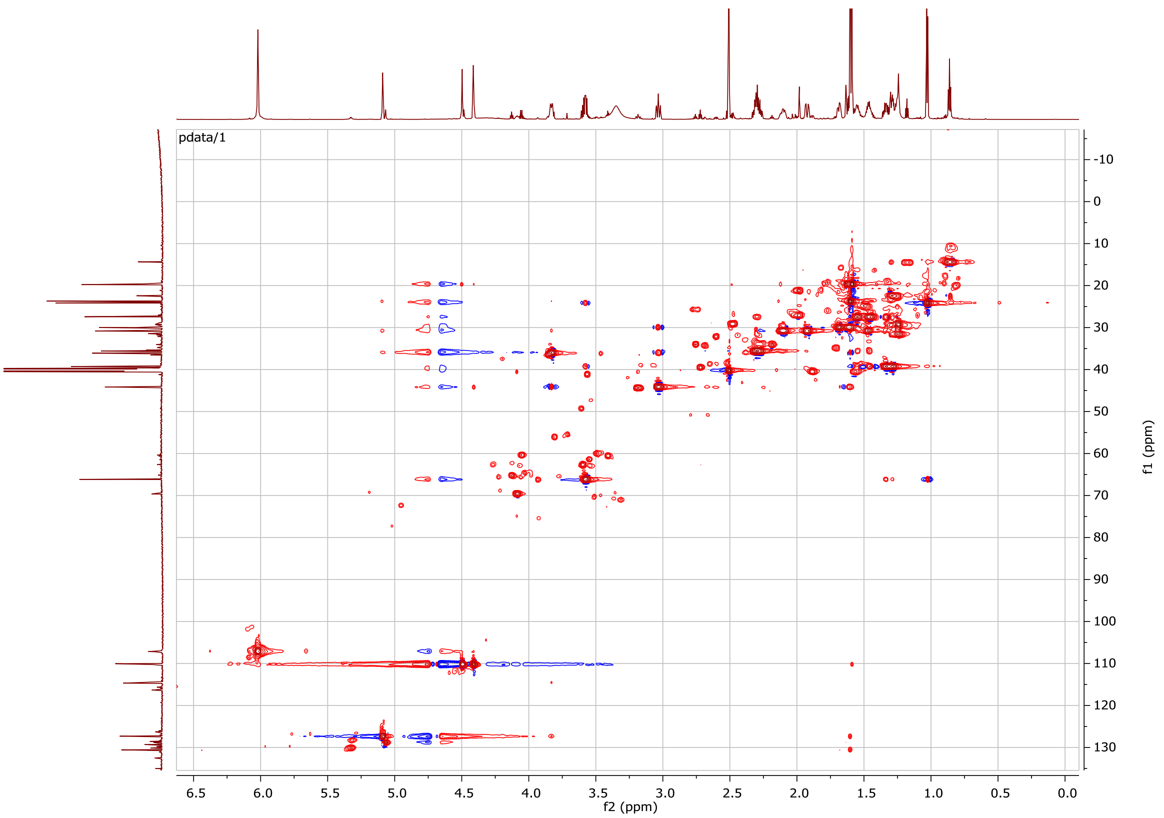

Figure S35. HSQC (^{1}H-^{13}C) NMR spectrum of metabolite **4**

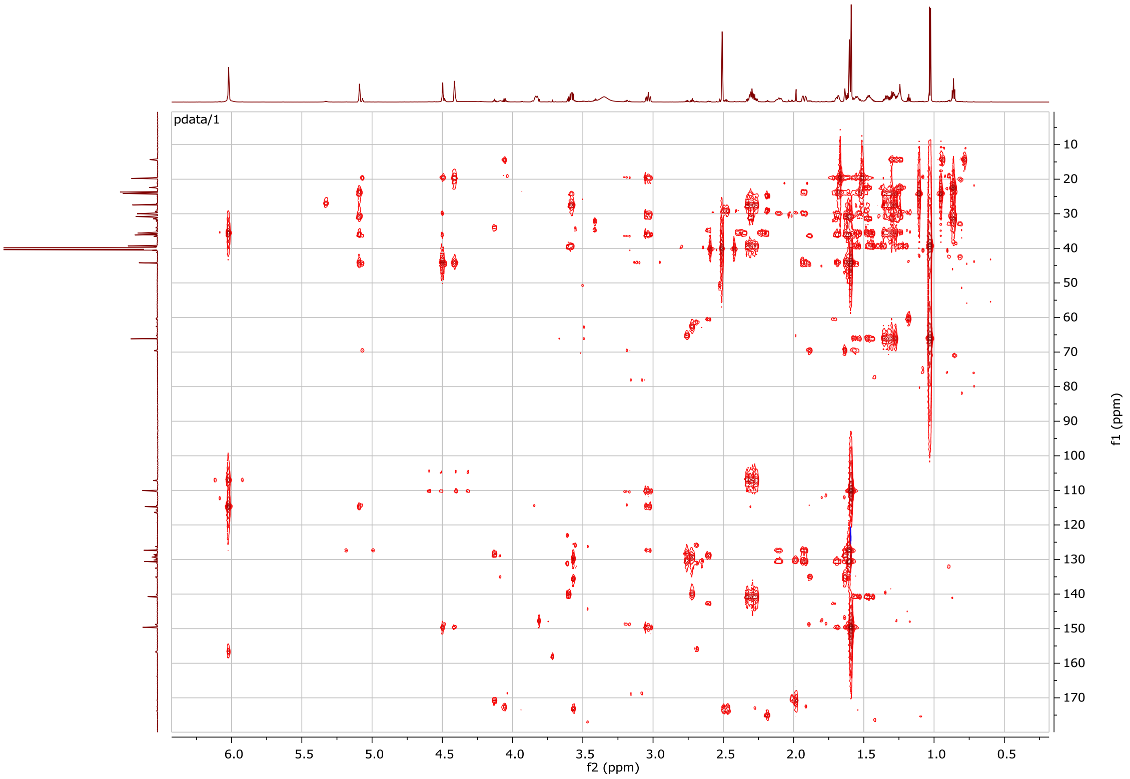

Figure S36. HMBC (long distance correlation ^{1}H-^{13}C) spectrum of metabolite **4**

5. **Metabolite 5: 4,4”-dihydroxycannabidiol**

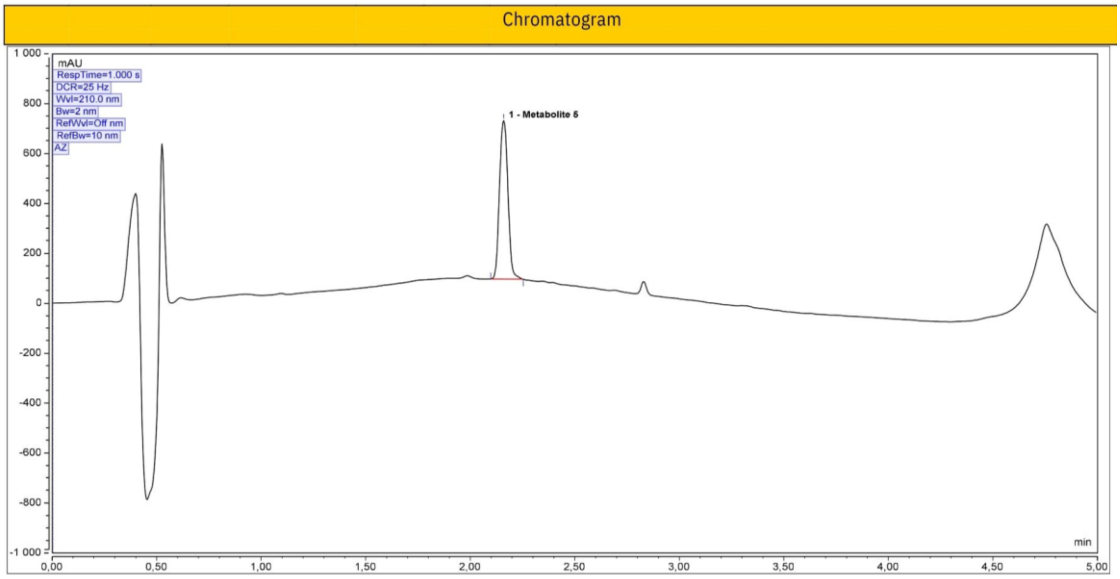

Figure S37. UHPLC-DAD chromatogram of metabolite **5**

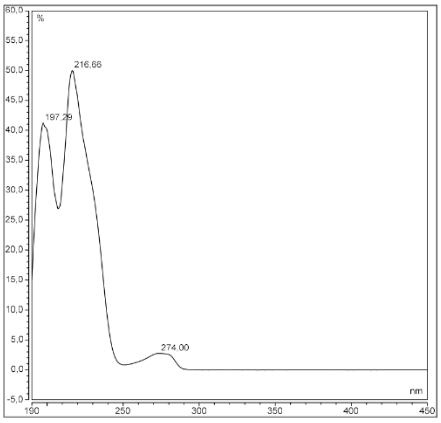

Figure S38. UV spectrum of metabolite **5**

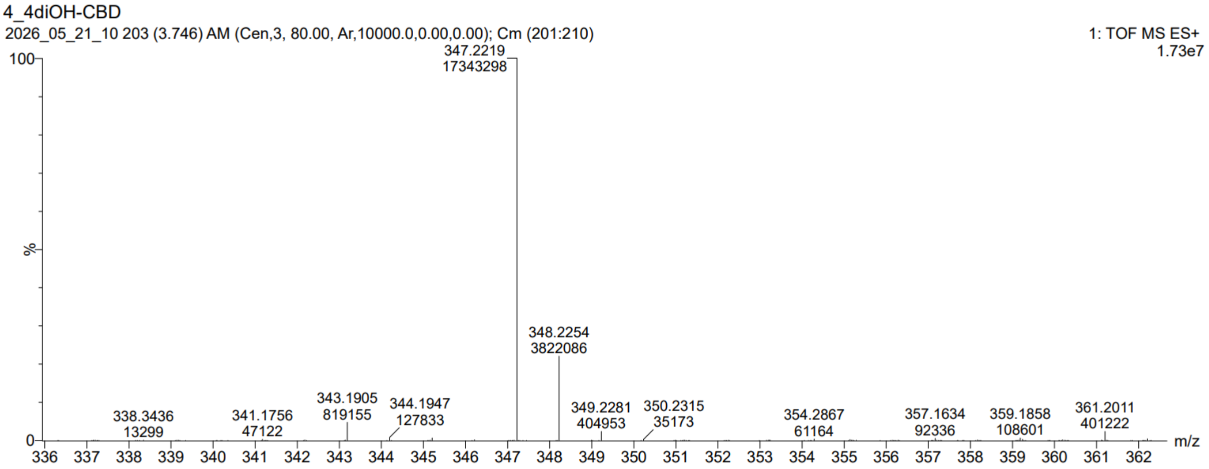

Figure S39. Mass spectrum of metabolite **5**

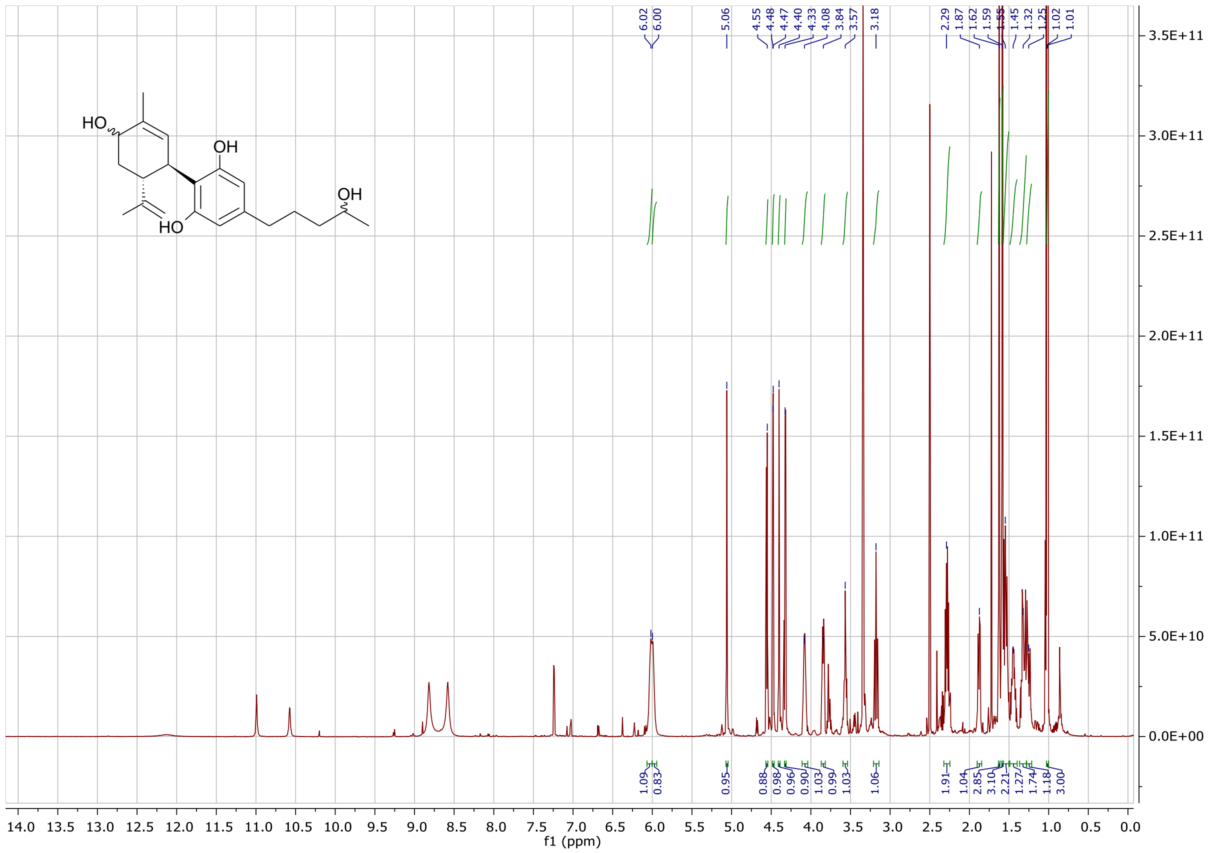

Figure S40. ^{1}H NMR spectrum of metabolite **5**

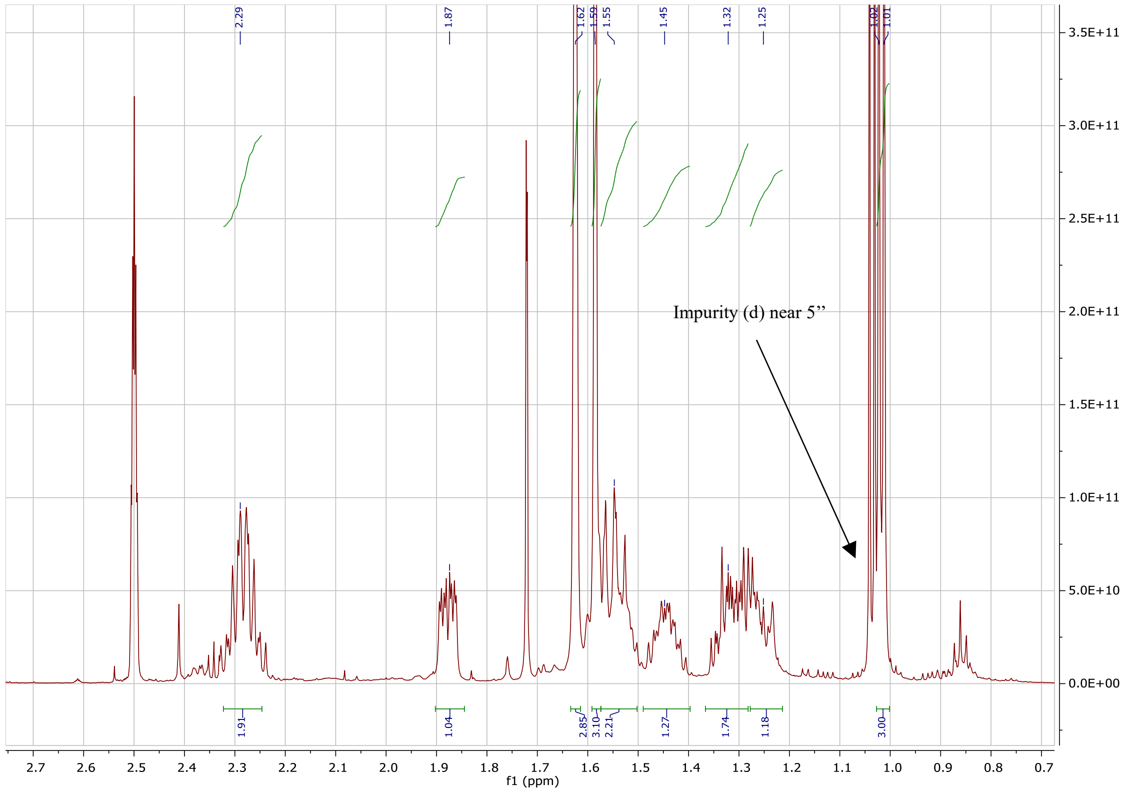

Figure S41. ^{1}H NMR (0.7-2.5) spectrum of metabolite **5**

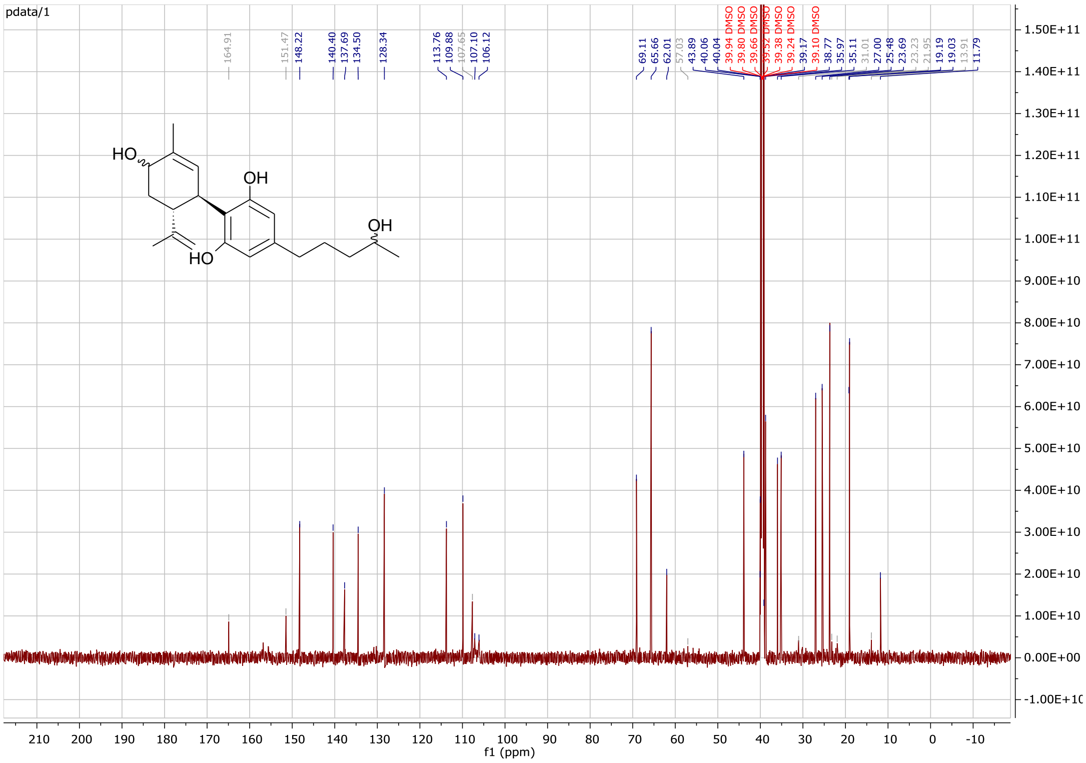

Figure S42. ^{13}C NMR spectrum of metabolite **5**

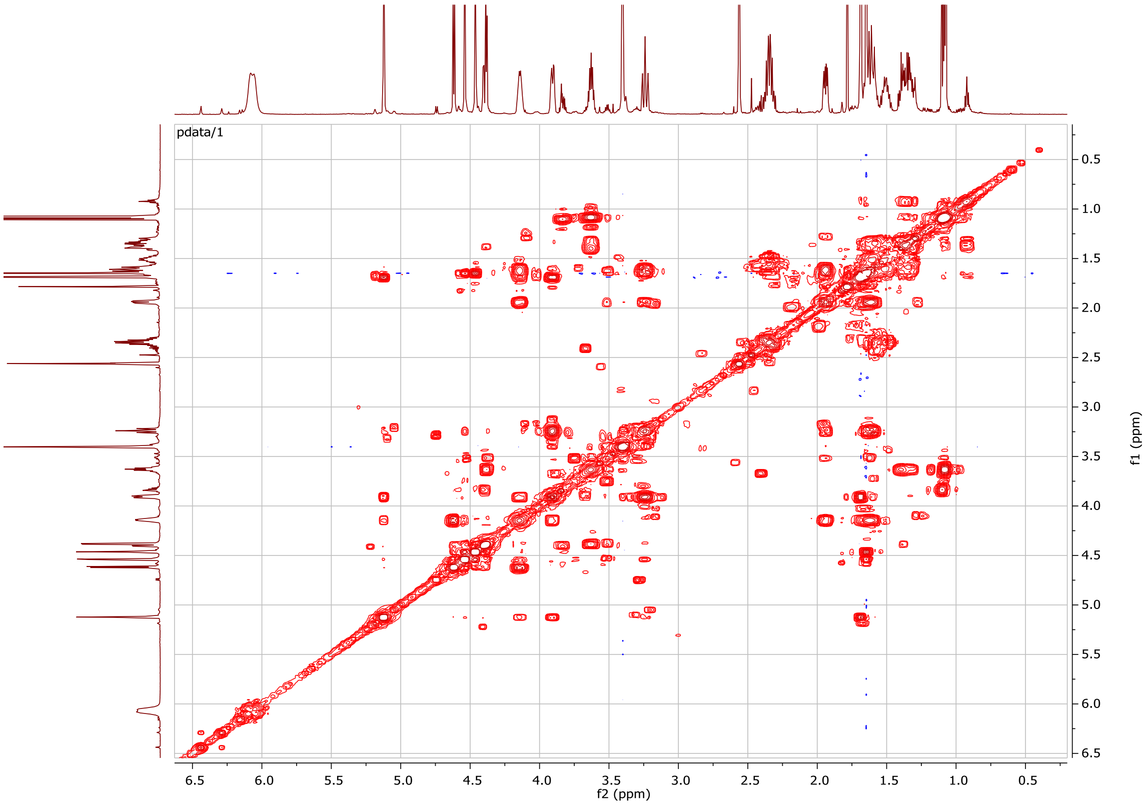

Figure S43. COSY (^{1}H-^{1}H) NMR spectrum of metabolite **5**

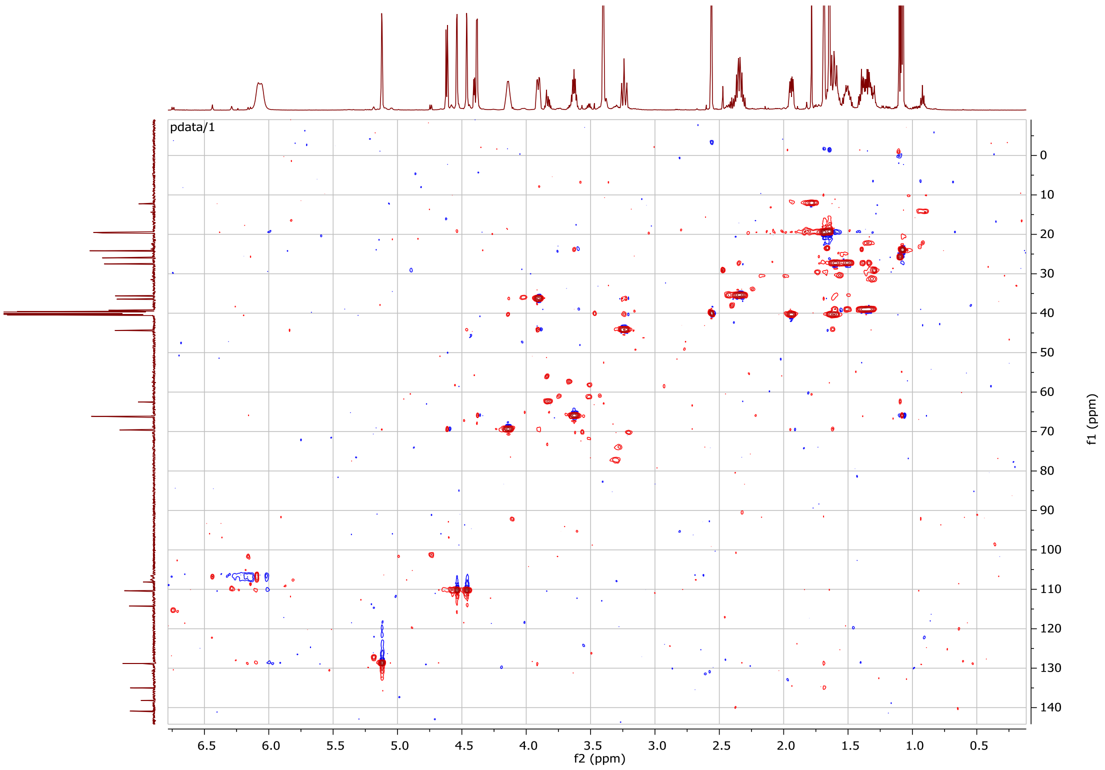

Figure S44. HSQC (1H-13C) NMR spectrum of metabolite **5**

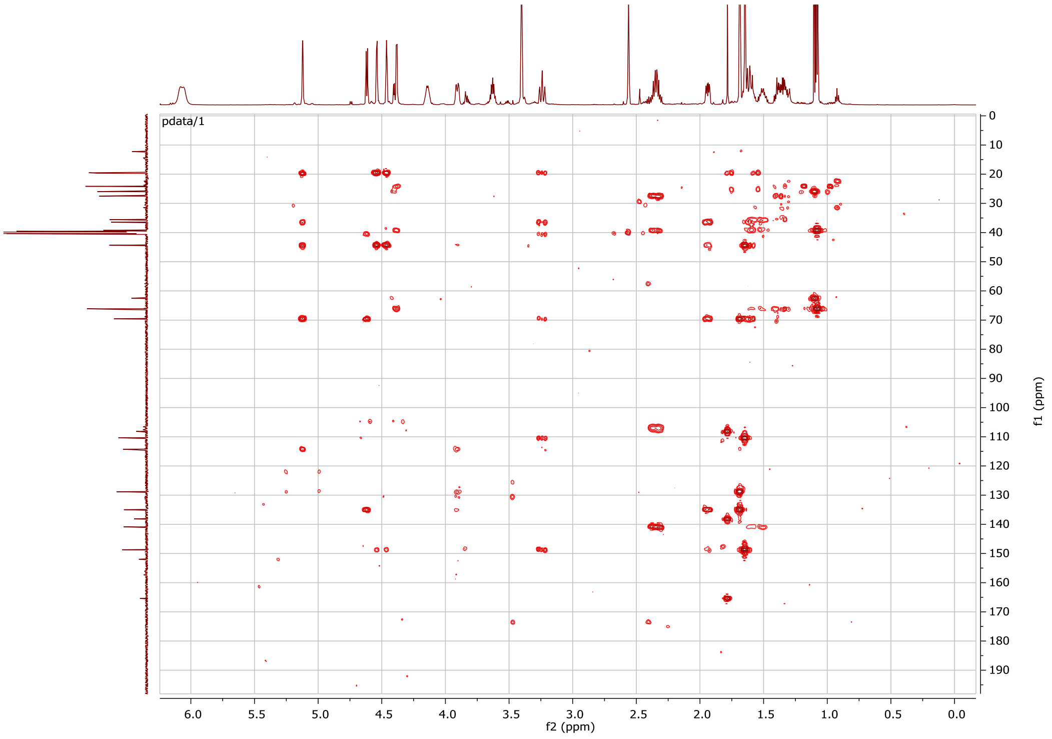

Figure S45. HMBC (long distance correlation ^{1}H-^{13}C) spectrum of metabolite **5**

6. Metabolite 6: 7,4”-dihydroxycannabidiol

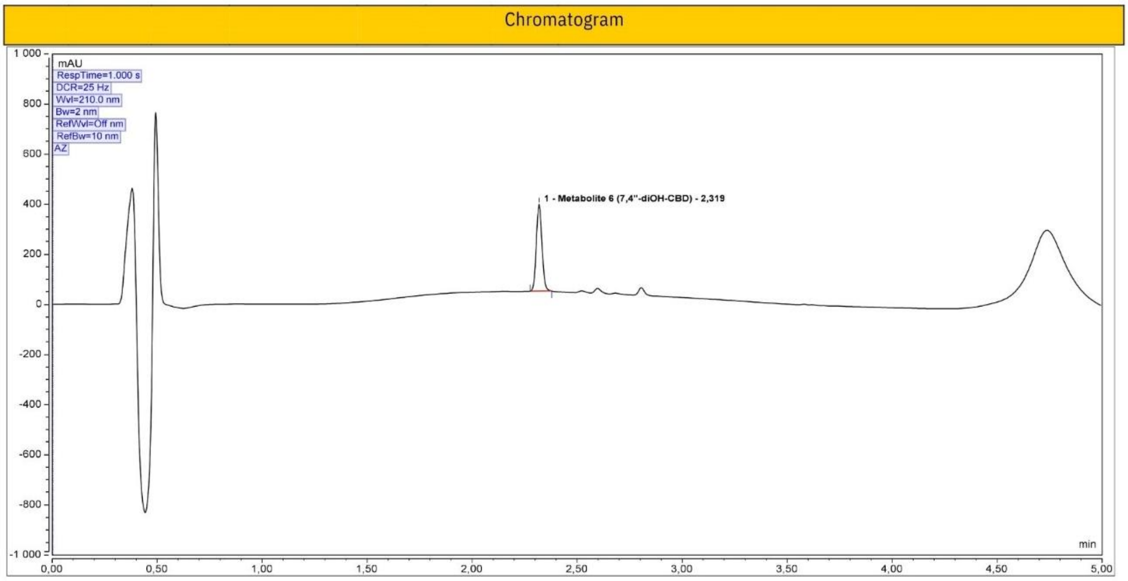

Figure S46. UHPLC-DAD chromatogram of metabolite **6**

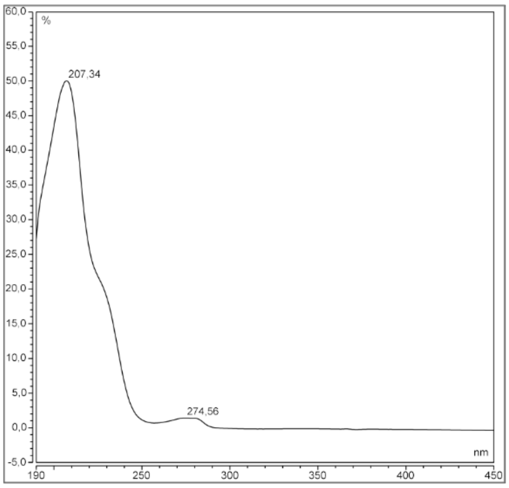

Figure S47. UV spectrum of metabolite 6

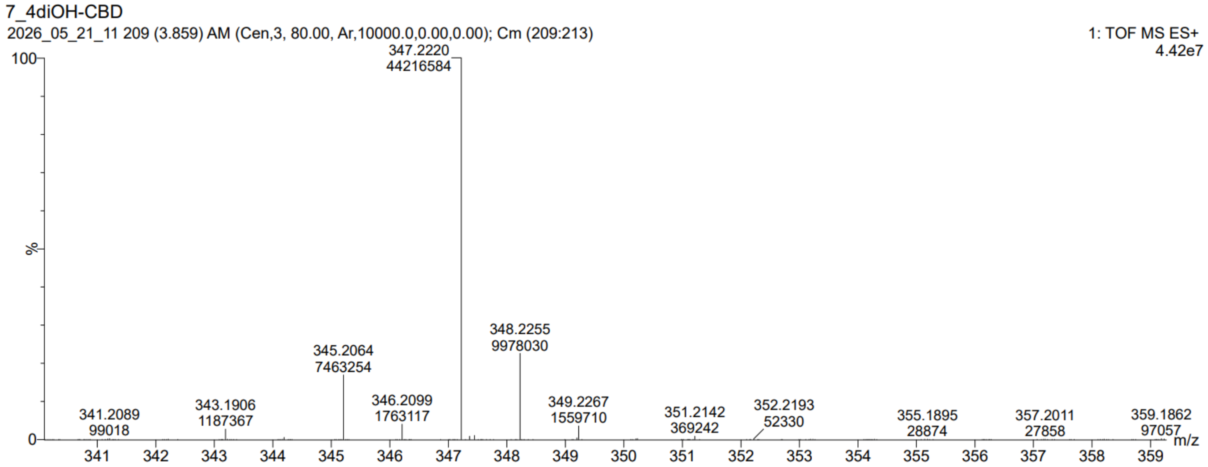

Figure S48. Mass spectrum of metabolite **6**

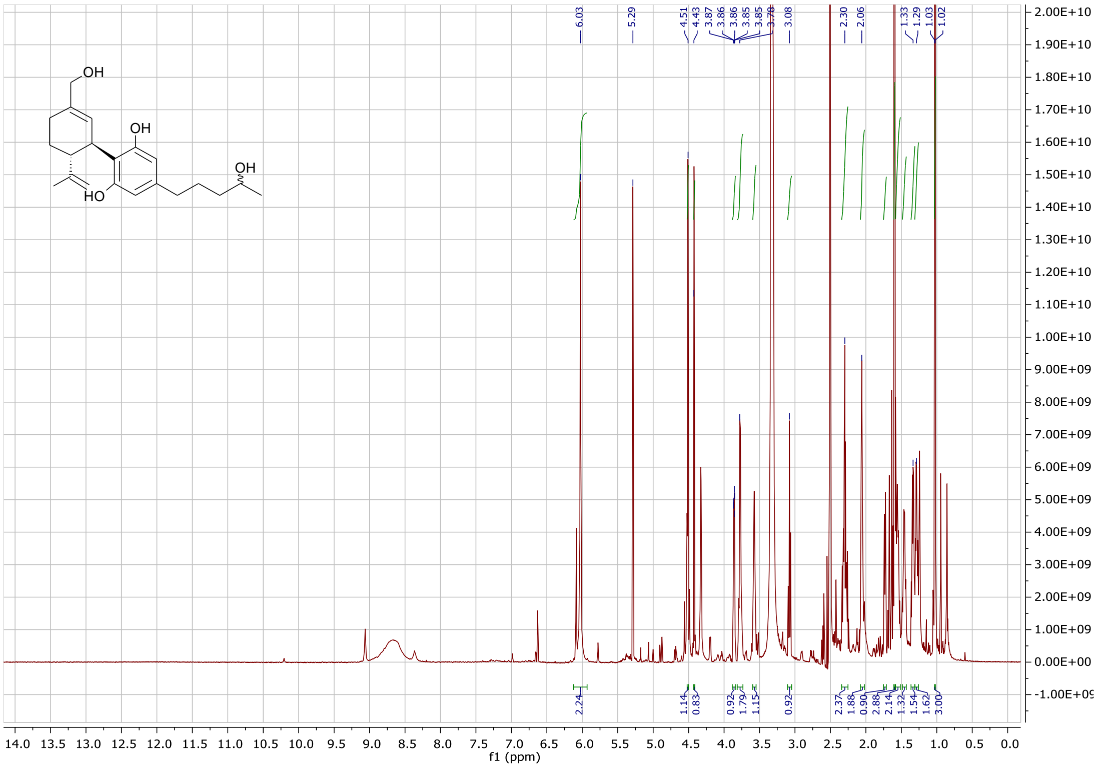

Figure S49. ^{1}H NMR spectrum of metabolite **6**

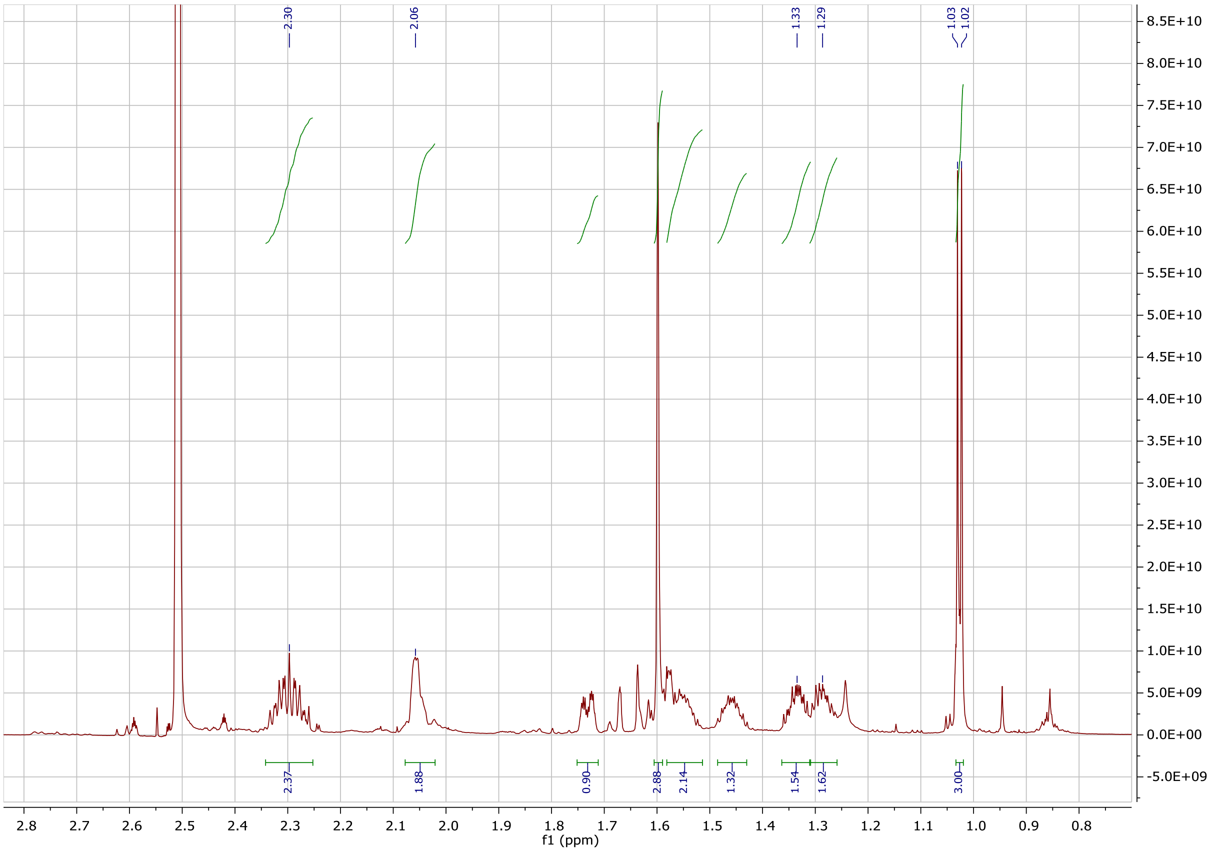

Figure S50. ^{1}H NMR (0.8-2.8) spectrum of metabolite **6**

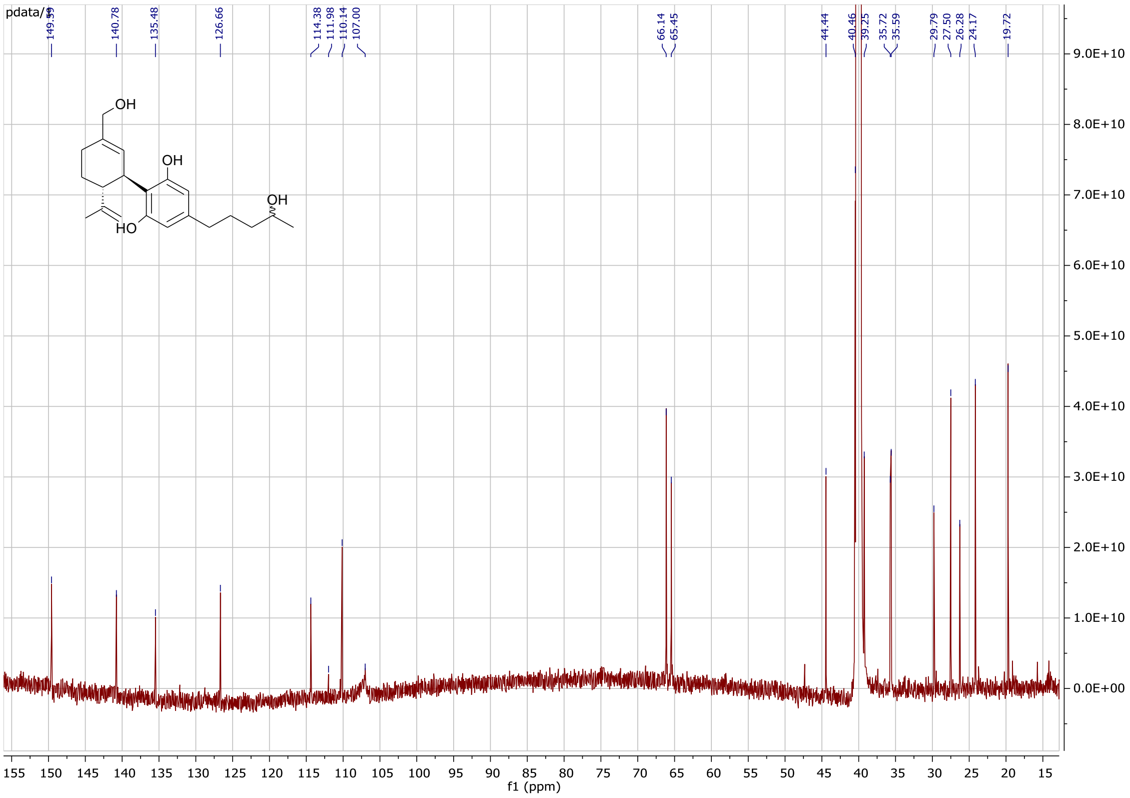

Figure S51. ^{13}C NMR spectrum of metabolite **6**

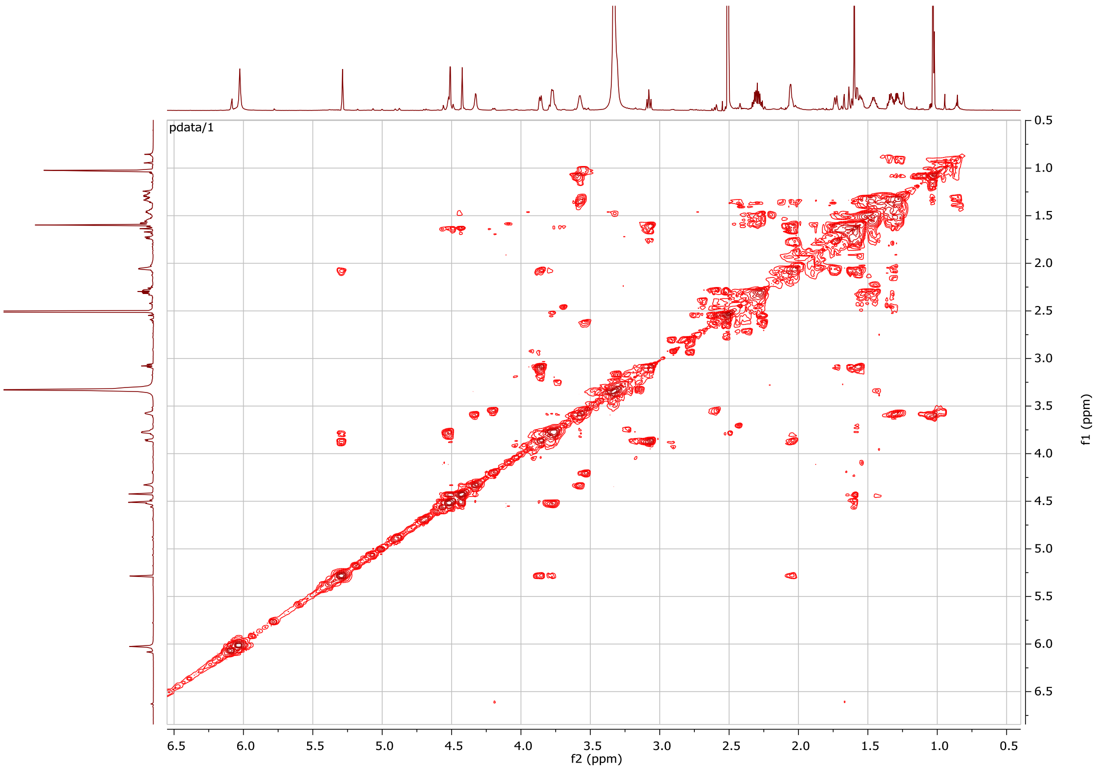

Figure S52. COSY (^{1}H-^{1}H) NMR spectrum of metabolite **6**

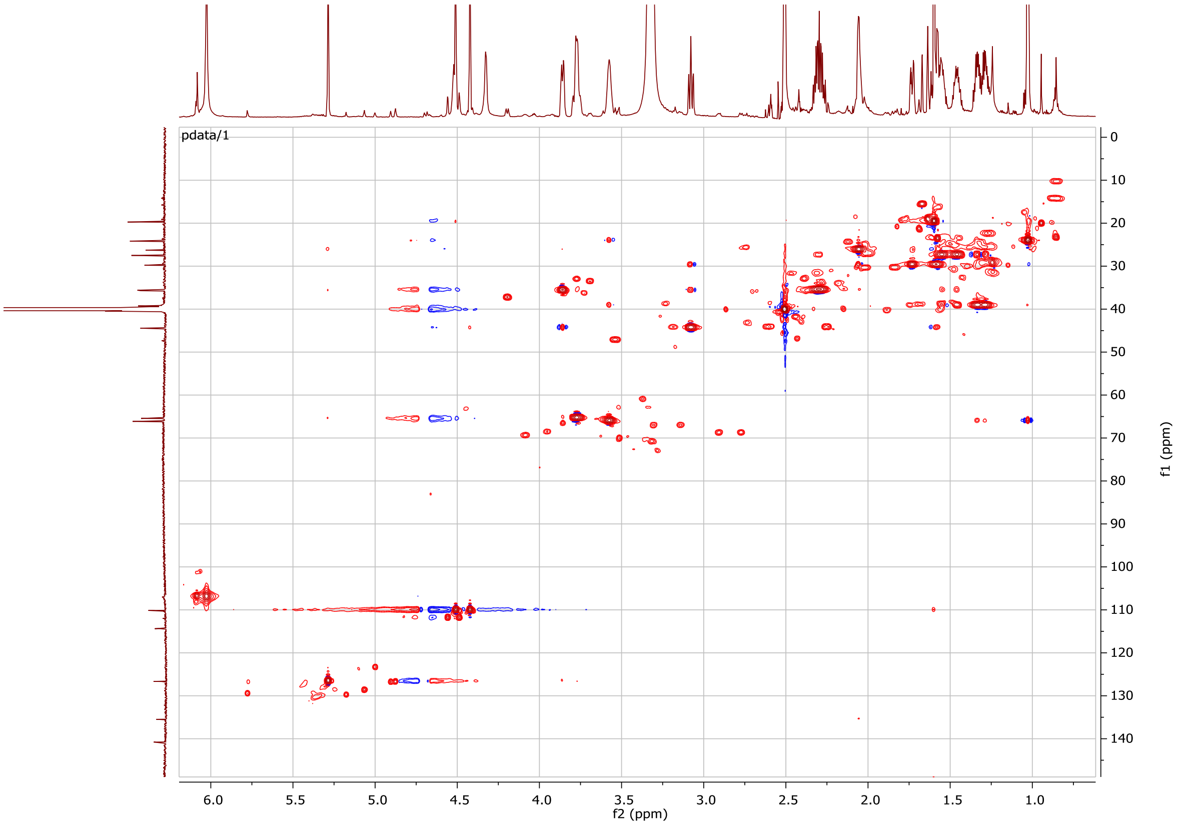

Figure S53. HSQC (^{1}H-^{13}C) NMR spectrum of metabolite **6**

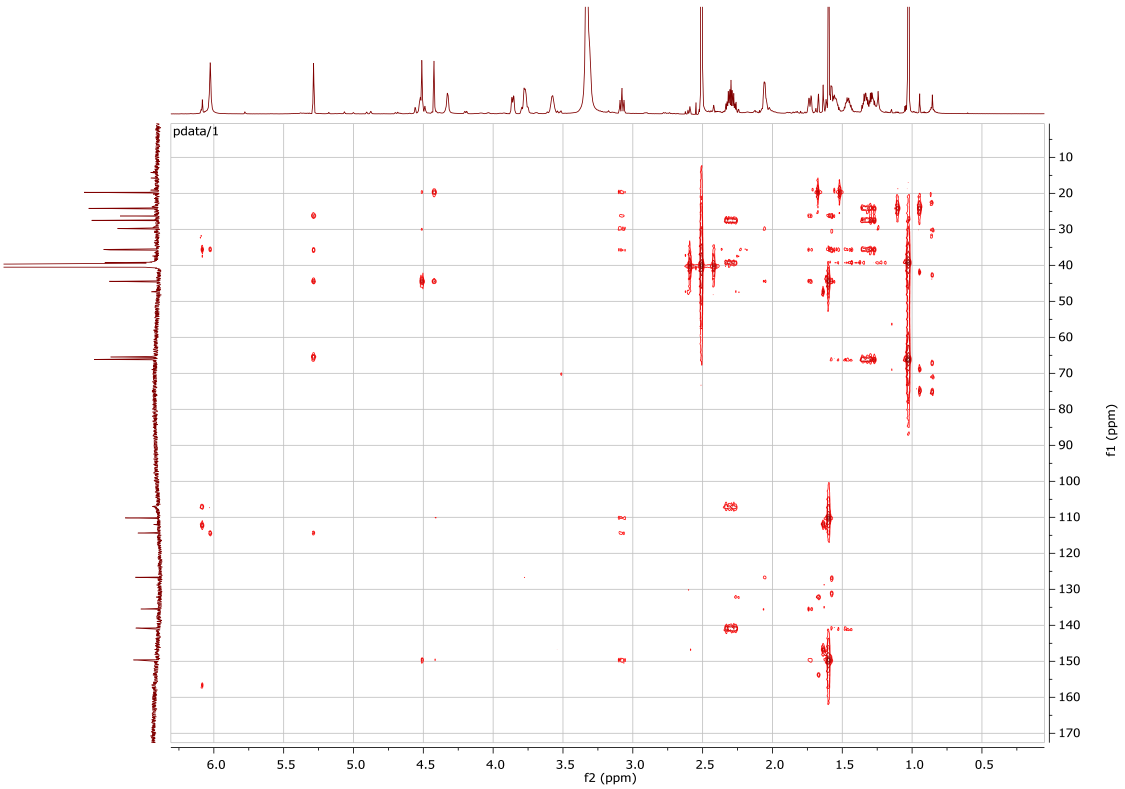

Figure S54. HMBC (long distance correlation ^{1}H-^{13}C) spectrum of metabolite **6**

7. Metabolite 7: 2’-*O*-(4’”-*O*-methyl-β-D-glucopyranosyl)cannabidiol

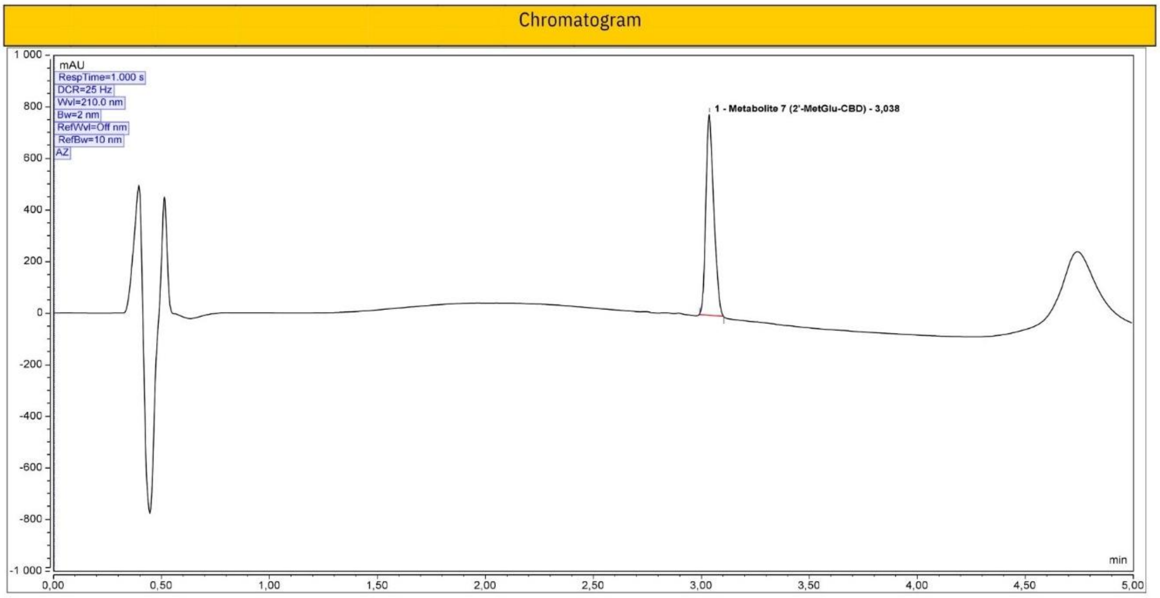

Figure S55. UHPLC-DAD chromatogram of metabolite **7**

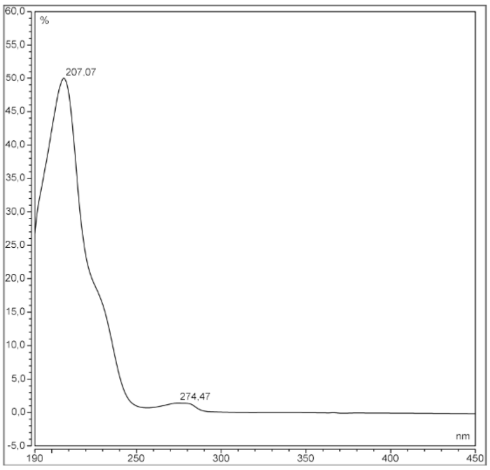

Figure S56. UV spectrum of metabolite **7**

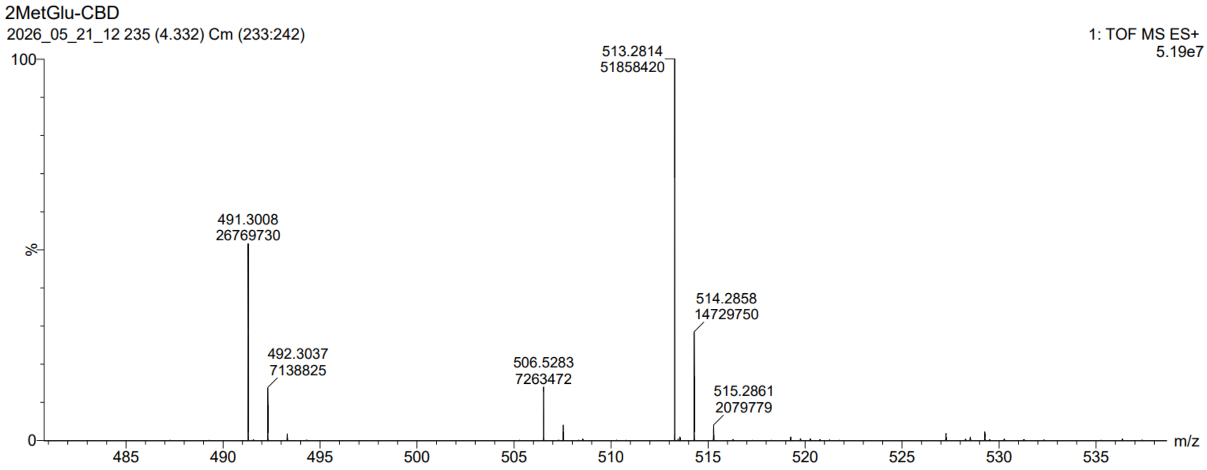

Figure S57. Mass spectrum of metabolite **7**

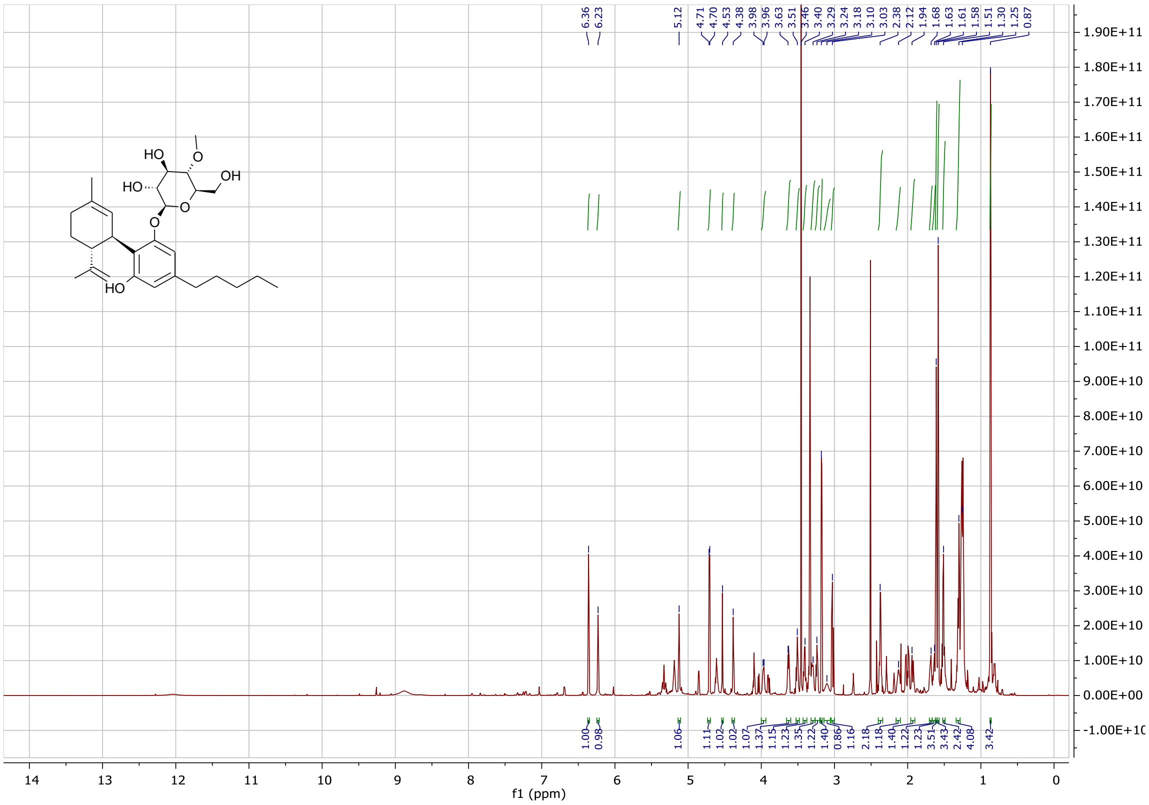

Figure S58. ^{1}H NMR spectrum of metabolite **7**

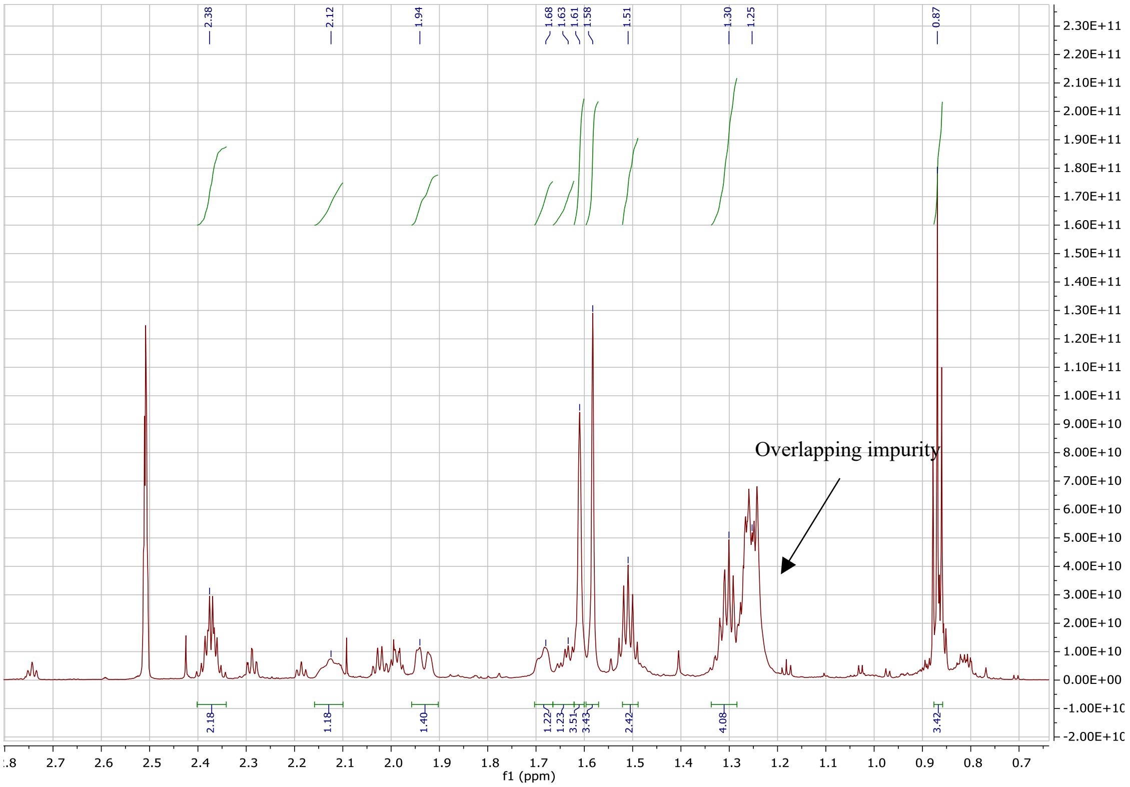

Figure S59. ^{1}H NMR (0.7-2.6) spectrum of metabolite **7**

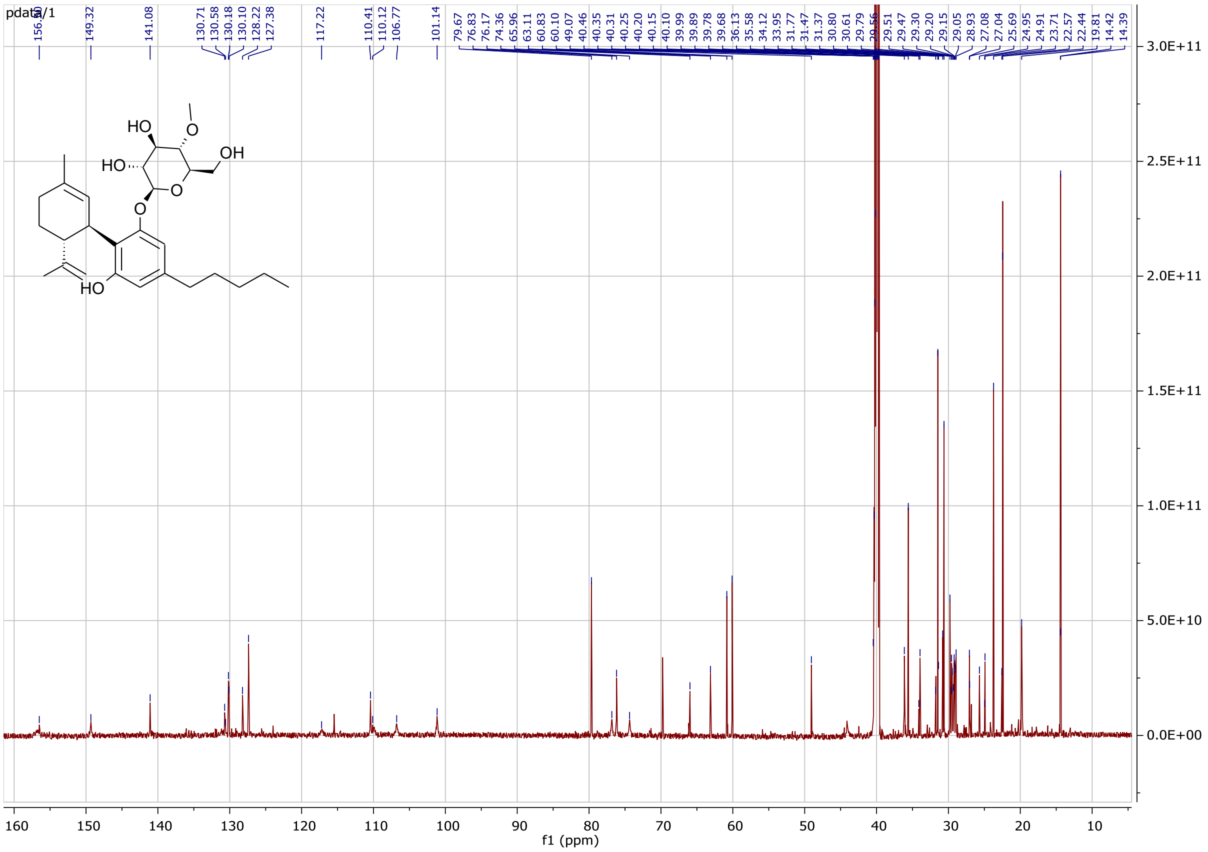

Figure S60. 13C NMR spectrum of metabolite **7**

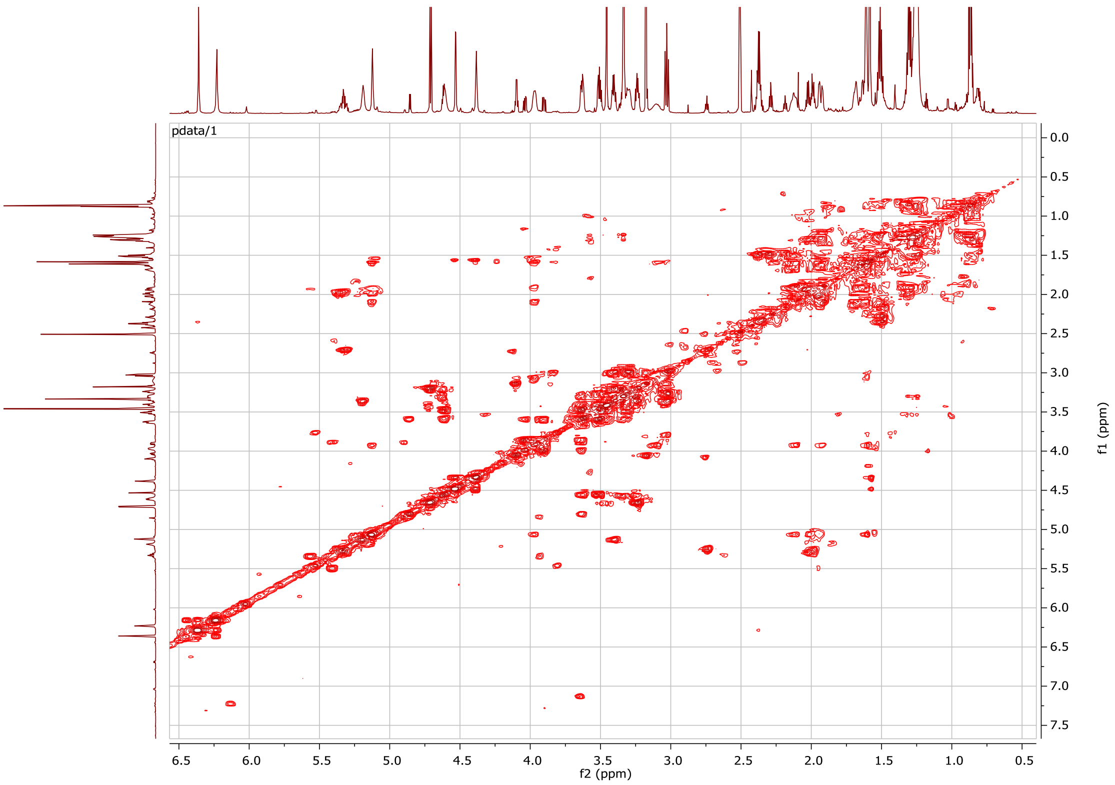

Figure S61. COSY (^{1}H-^{1}H) NMR spectrum of metabolite **7**

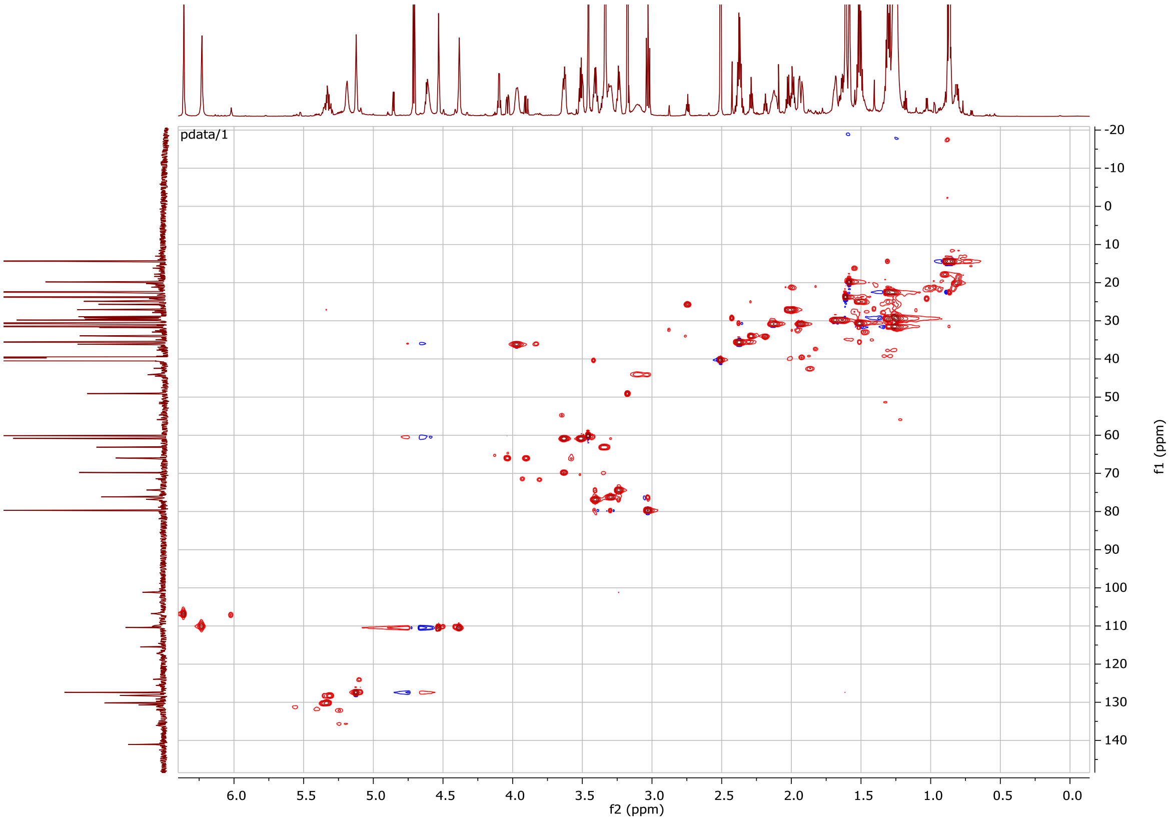

Figure S62. HSQC (^{1}H-^{13}C) NMR spectrum of metabolite **7**

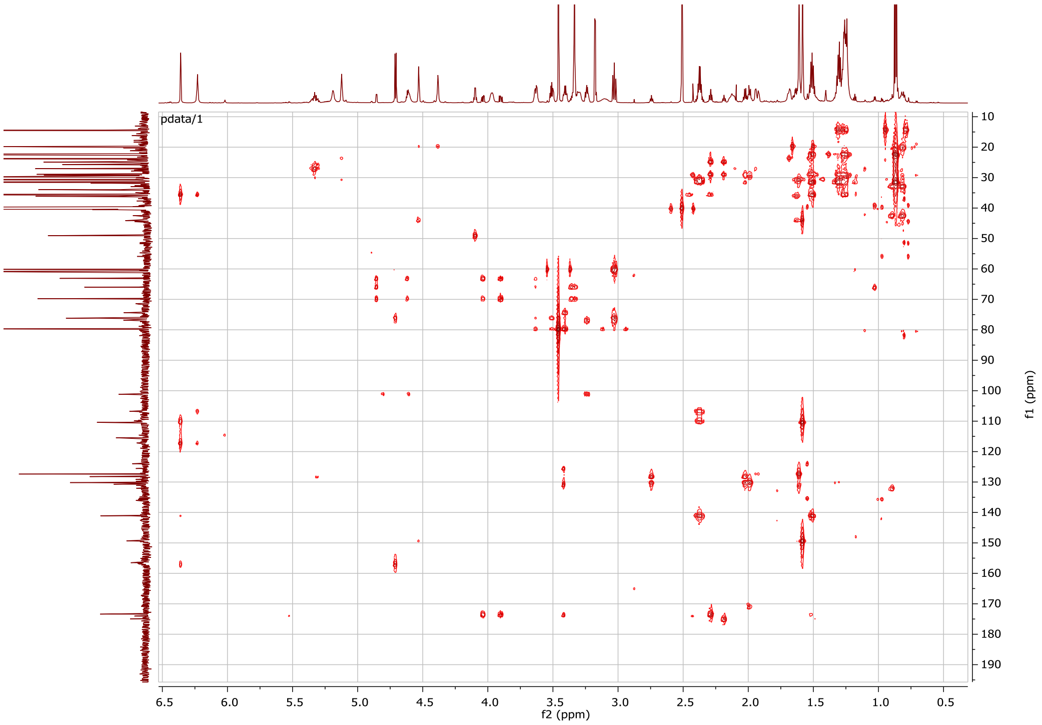

Figure S63. HMBC (long distance correlation ^{1}H-^{13}C) NMR spectrum of metabolite **7**

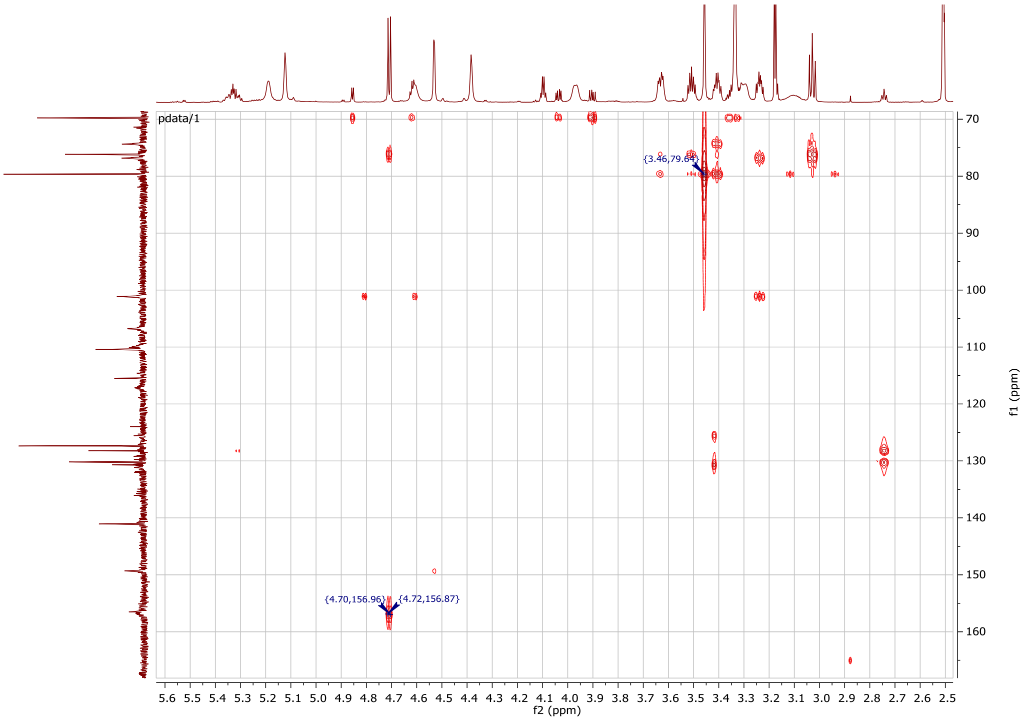

Figure S64. HMBC correlation between C1 carbon of glucose and C2 carbon of aromatic ring. Correlation between -OCH3 methoxyl group and C4 carbon of glucose.

8. Metabolite 8: 2'-*O*-(4'''-*O*-methyl-β-D-glucopyranosyl) 5''-hydroxycannabidiol

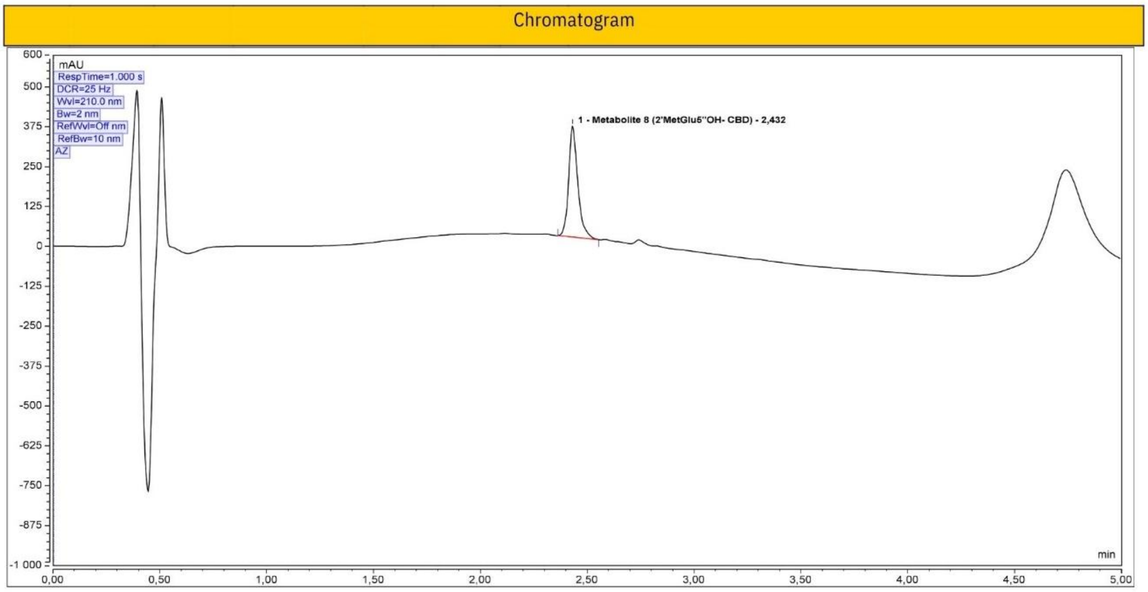

Figure S65. UHPLC-DAD chromatogram of metabolite **8**

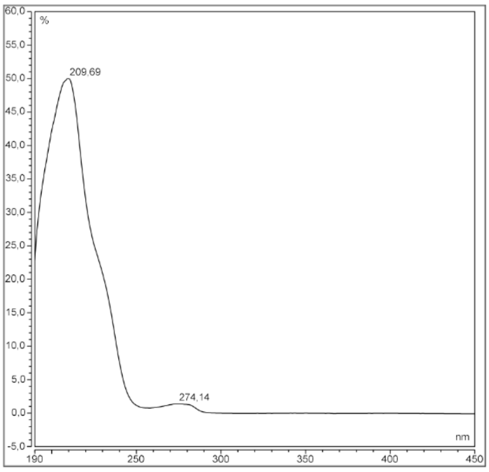

Figure S66. UV spectrum of metabolite **8**

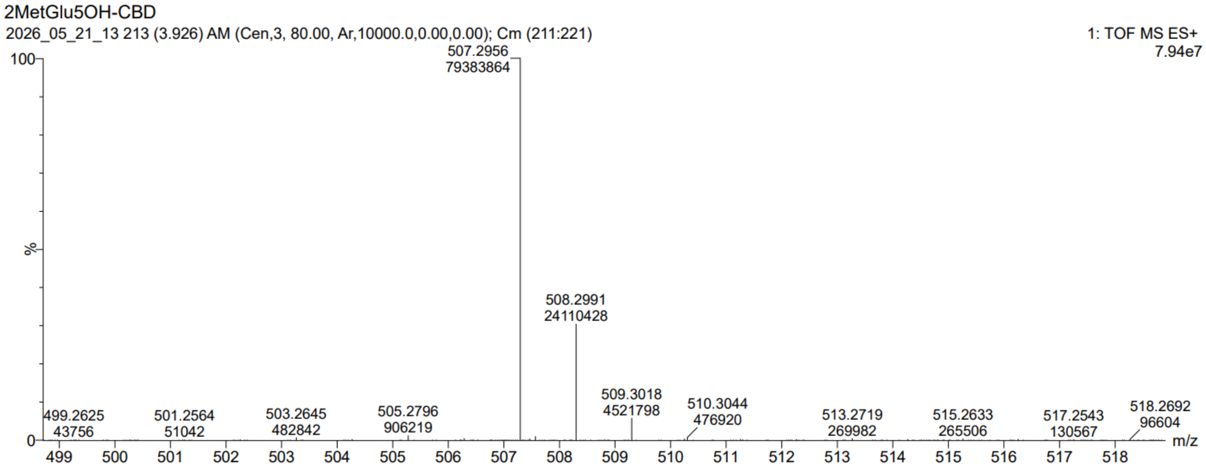

Figure S67. Mass spectrum of metabolite **8**

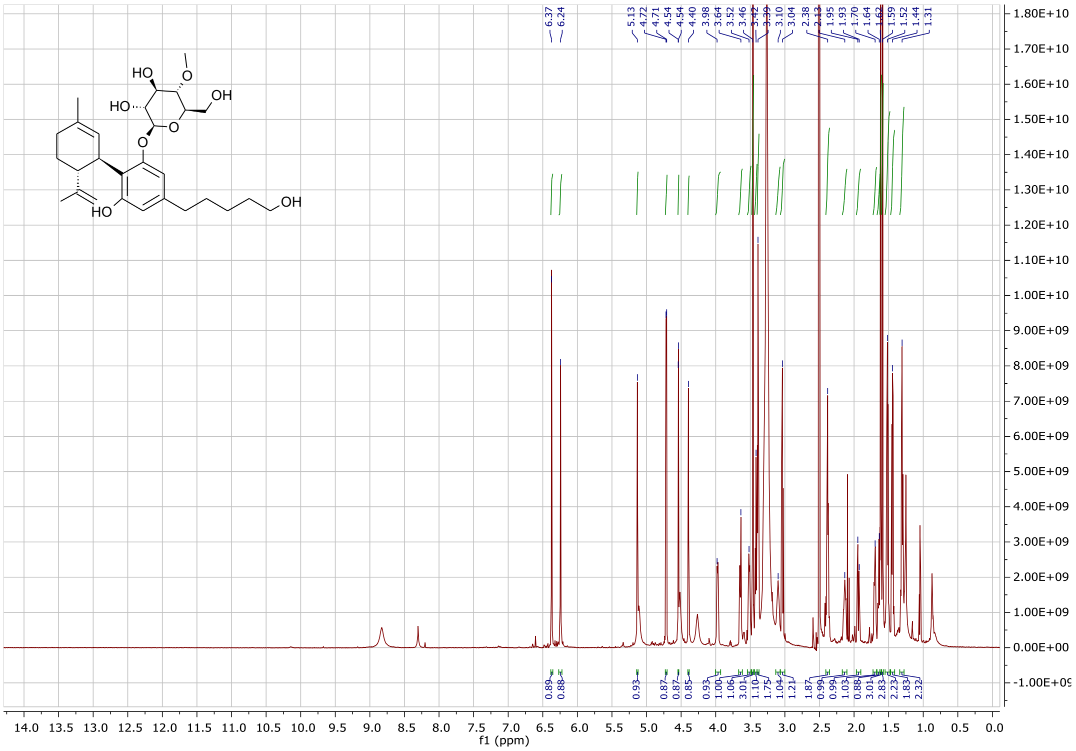

Figure S68. ^{1}H NMR spectrum of metabolite **8**

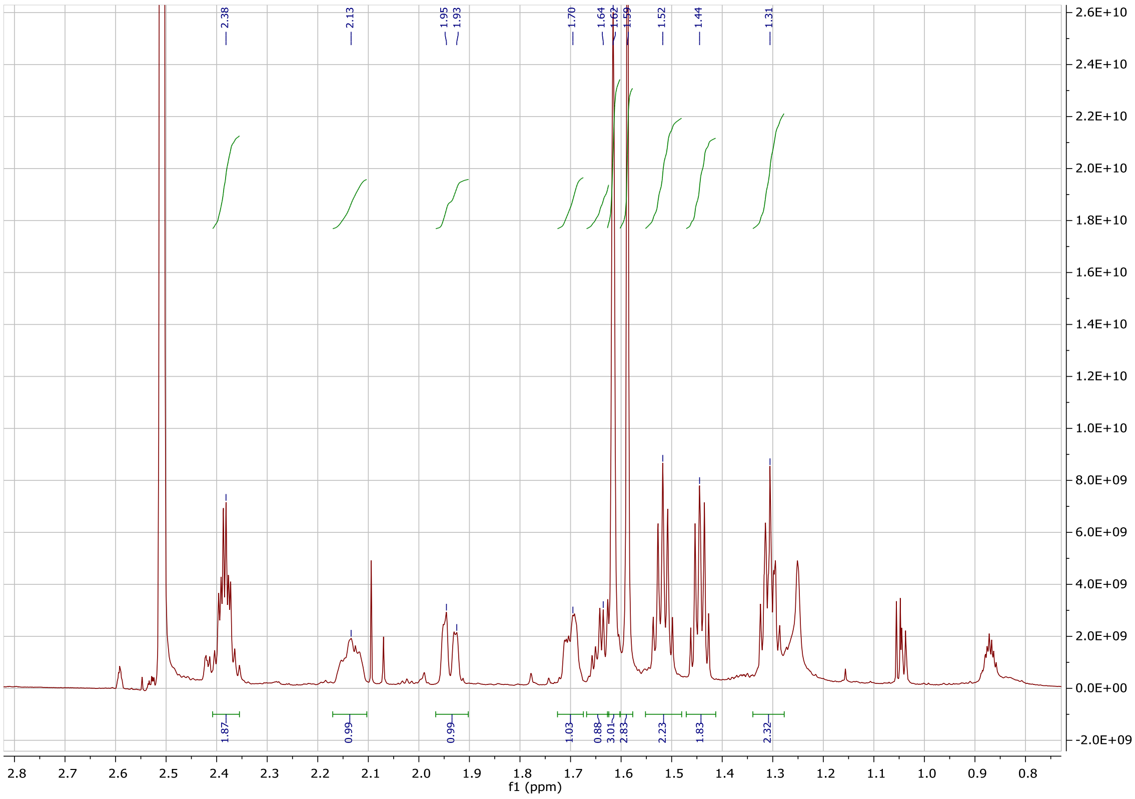

Figure S69. ^{1}H NMR (0.8-2.8) spectrum of metabolite **8**

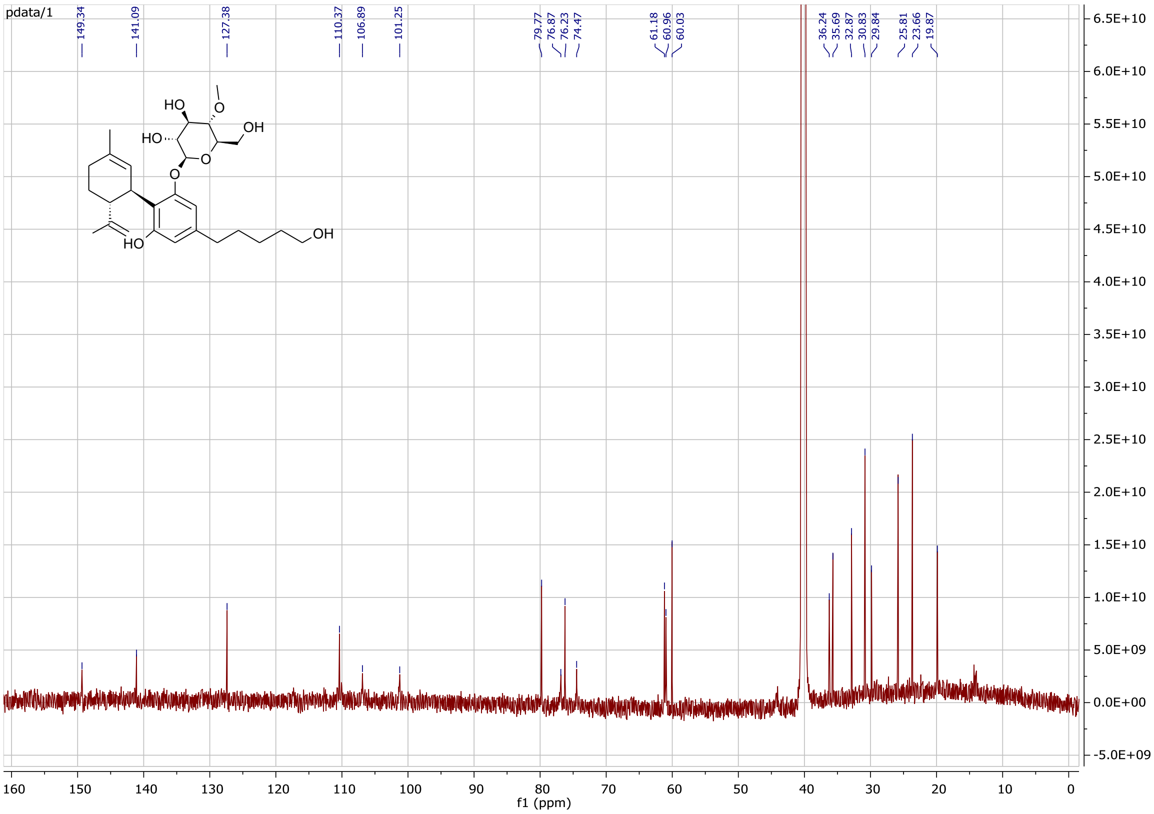

Figure S70. ^{13}C NMR spectrum of metabolite **8**

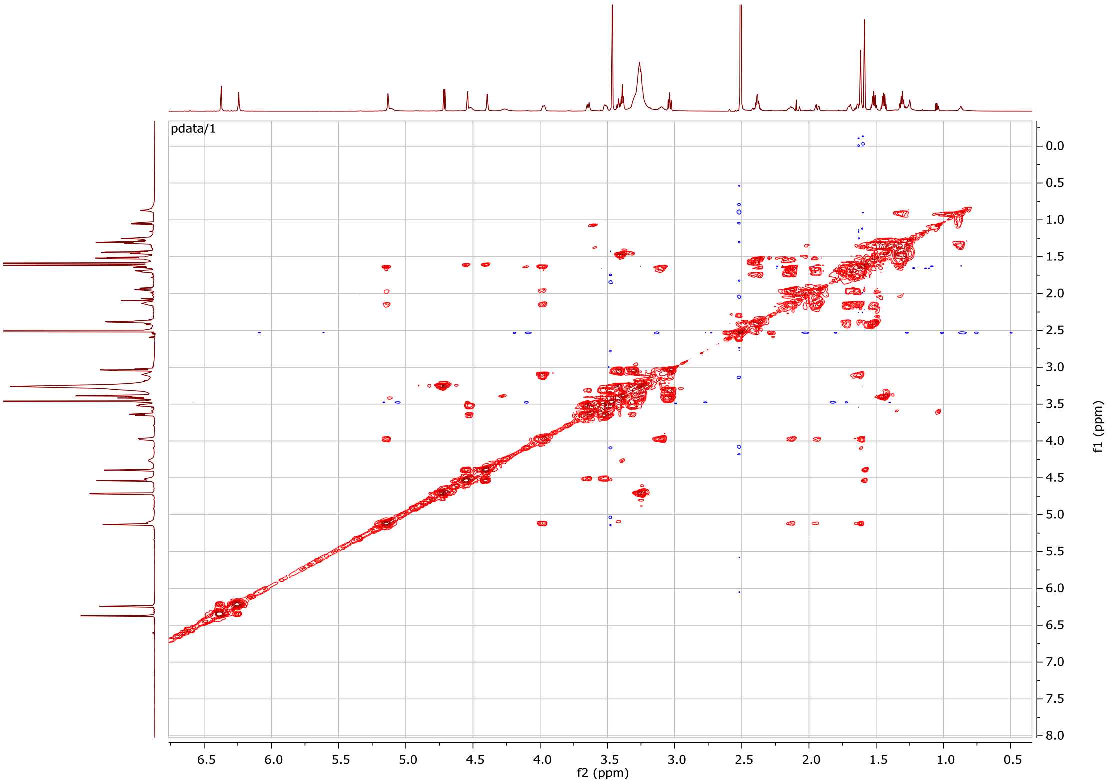

Figure S71. COSY (^{1}H-^{1}H) NMR spectrum of metabolite **8**

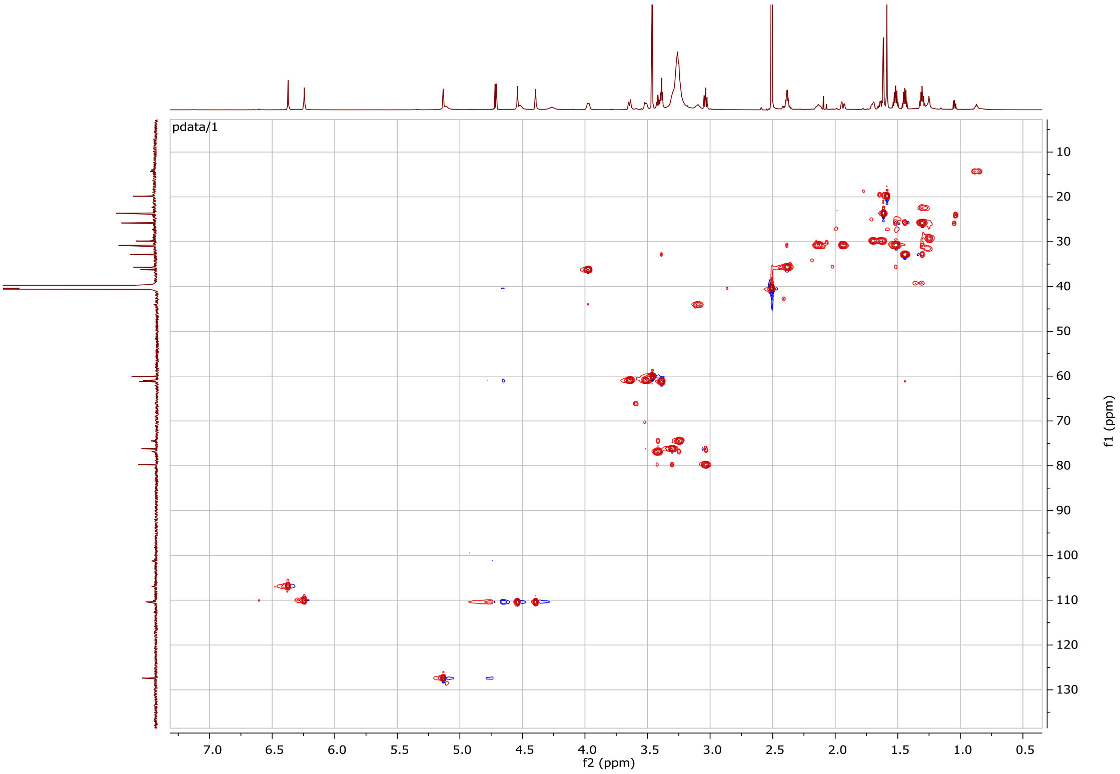

Figure S72. HSQC (^{1}H-^{13}C) NMR spectrum of metabolite **8**

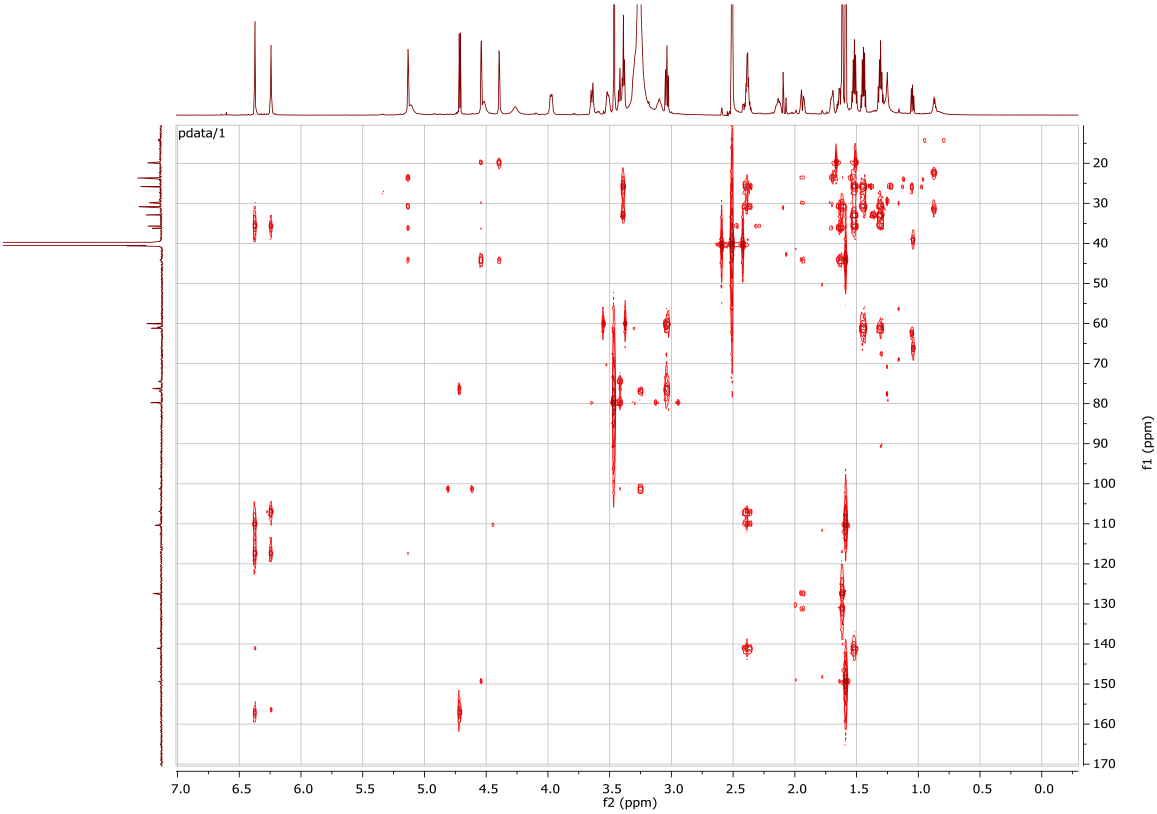

Figure S73. HMBC (long distance correlation ^{1}H-^{13}C) NMR spectrum of metabolite **8**

9. Metabolite 9: 4,2”-dihydroxycannabidiol

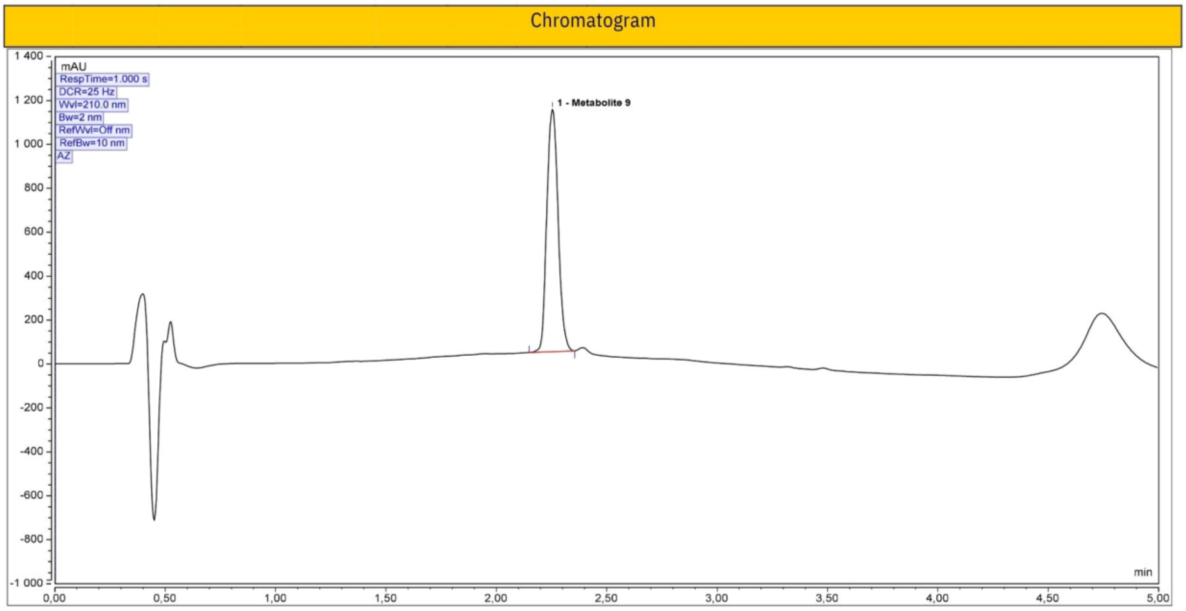

Figure S74. UHPLC-DAD chromatogram of metabolite **9**

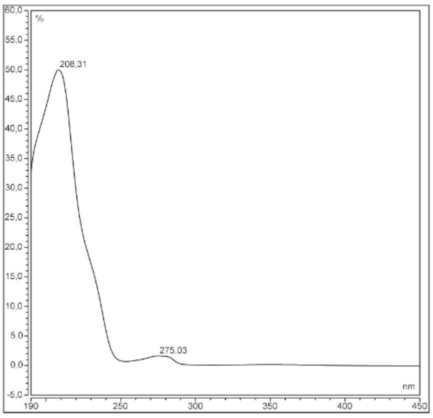

Figure S75. UV spectrum of metabolite **9**

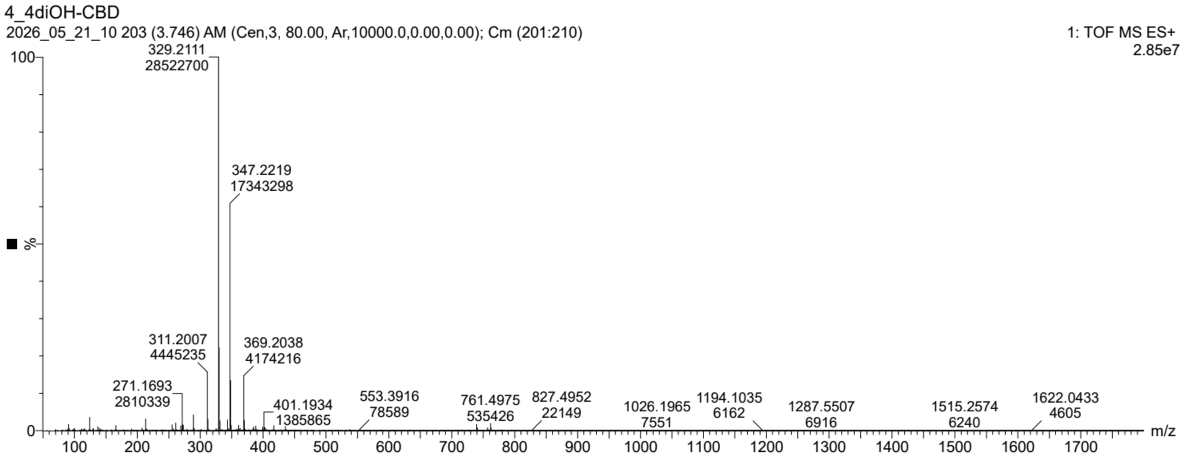

Figure S76. Mass spectrum of metabolite **9**

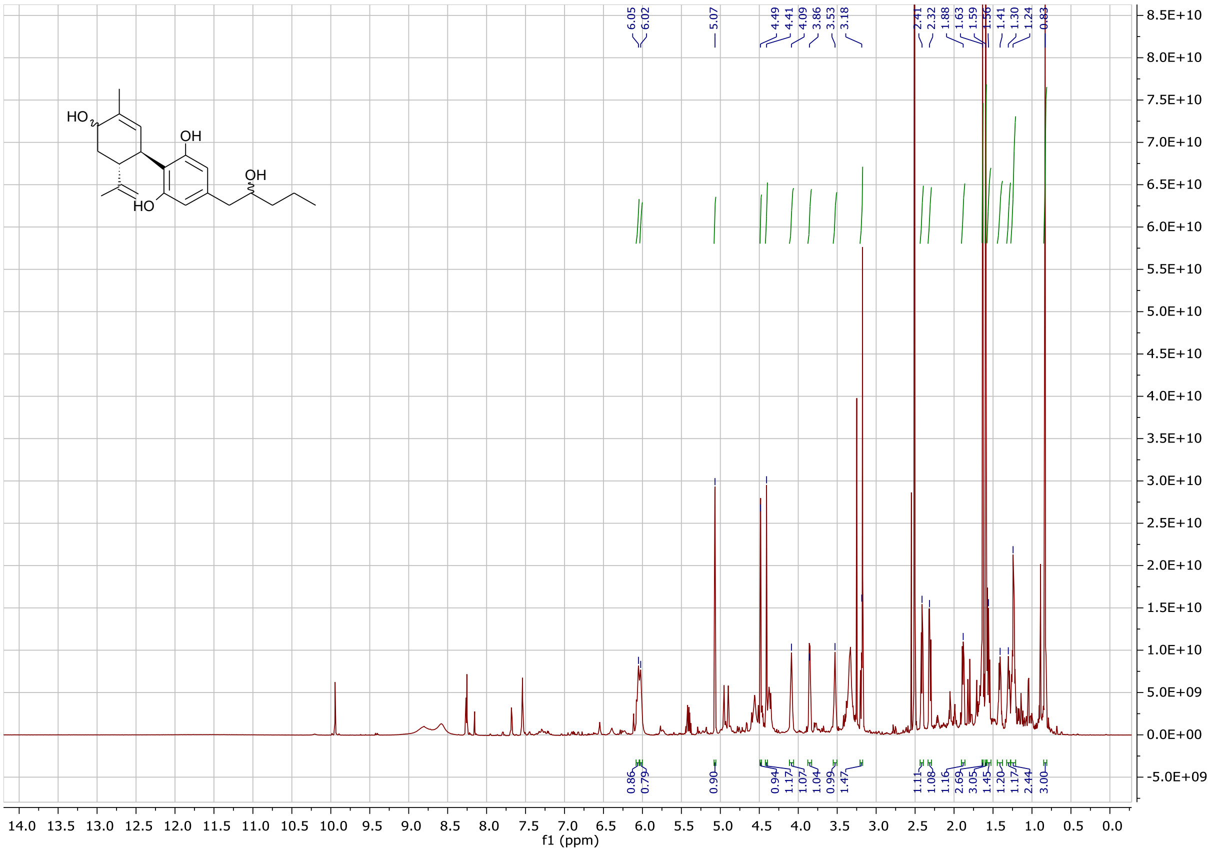

Figure S77. ^{1}H NMR spectrum of metabolite **9**

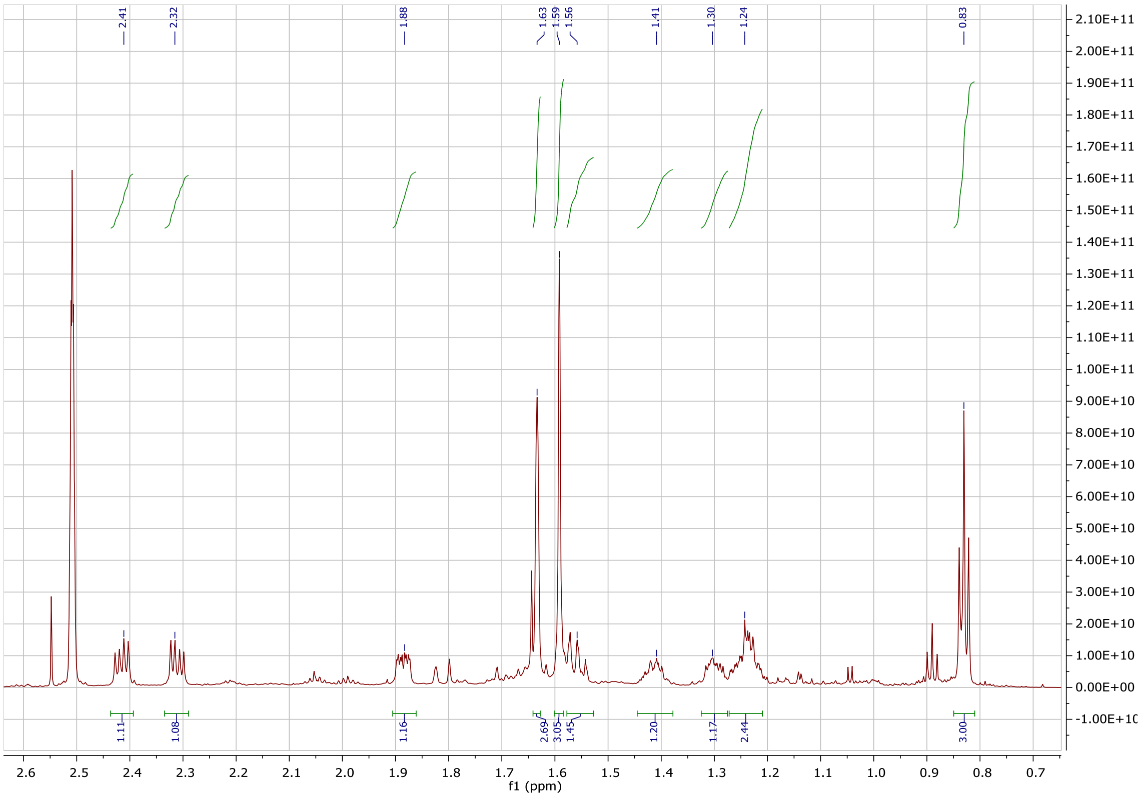

Figure S78. ^{1}H NMR (0.7-2.6 ppm) spectrum of metabolite **9**

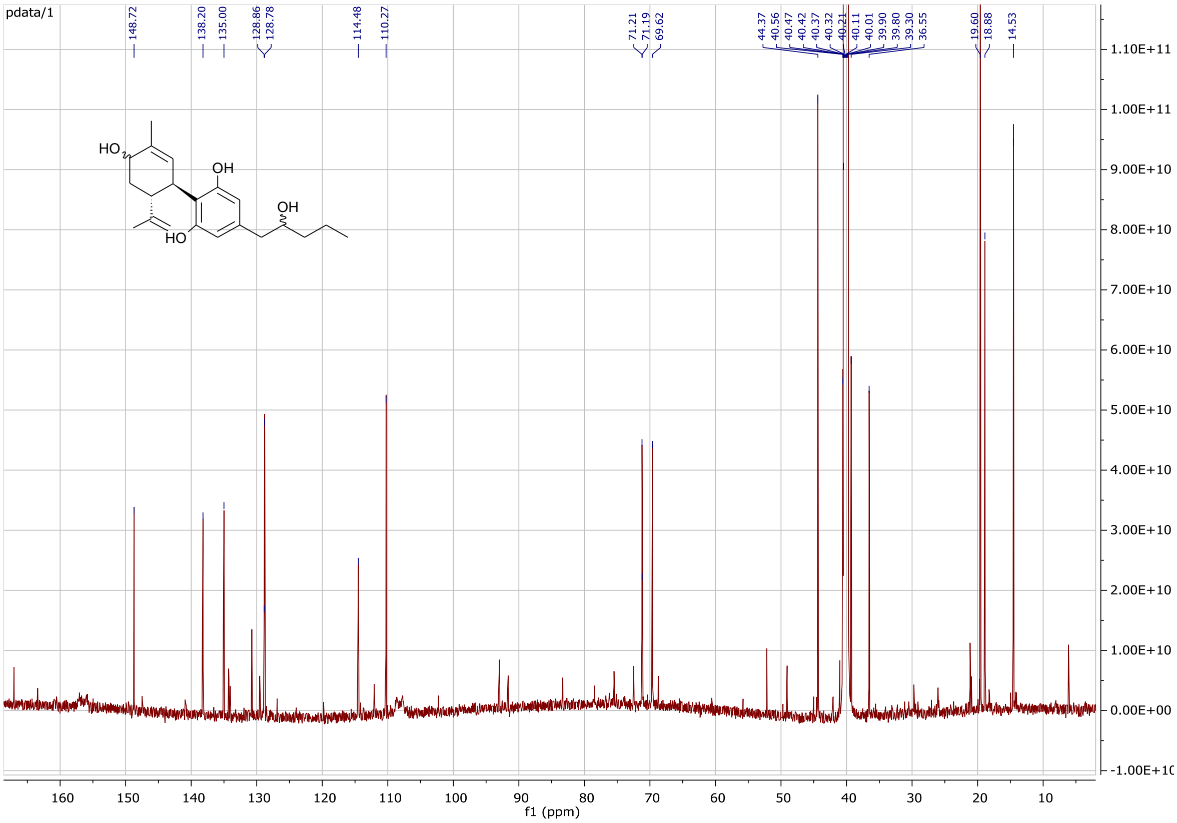

Figure S79. ^{13}C NMR spectrum of metabolite **9**

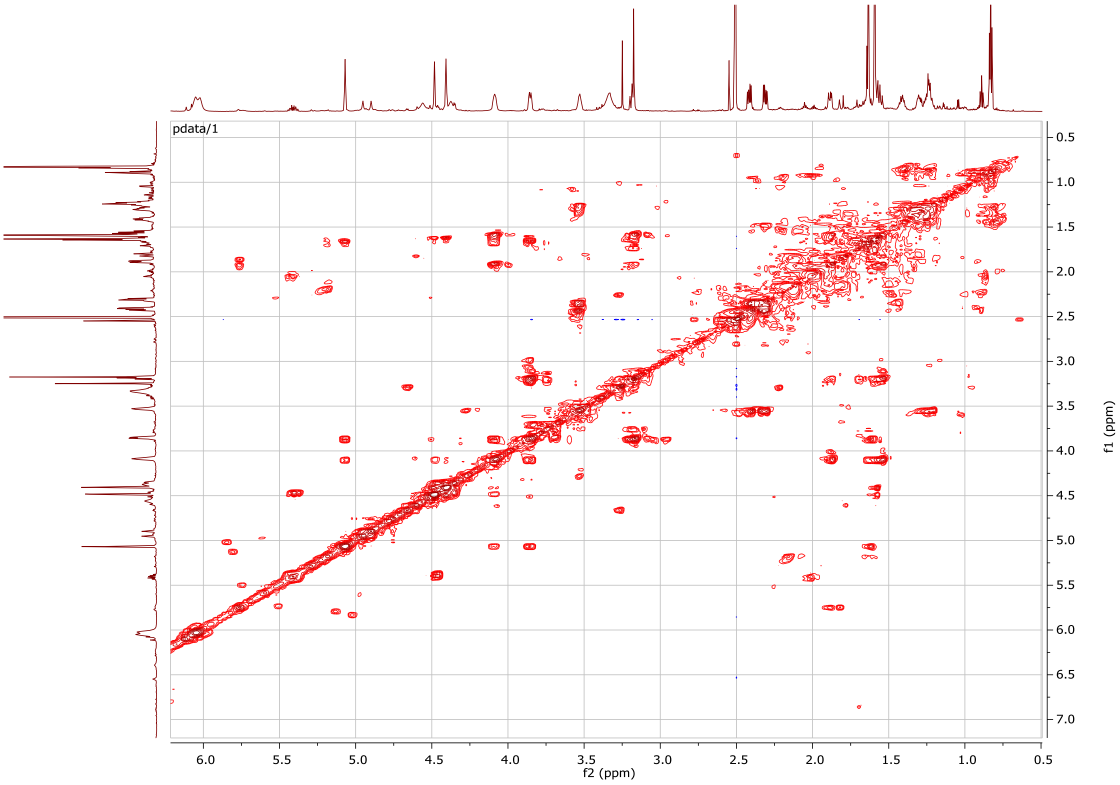

Figure S80. COSY (^1H-^1H) NMR spectrum of metabolite **9**

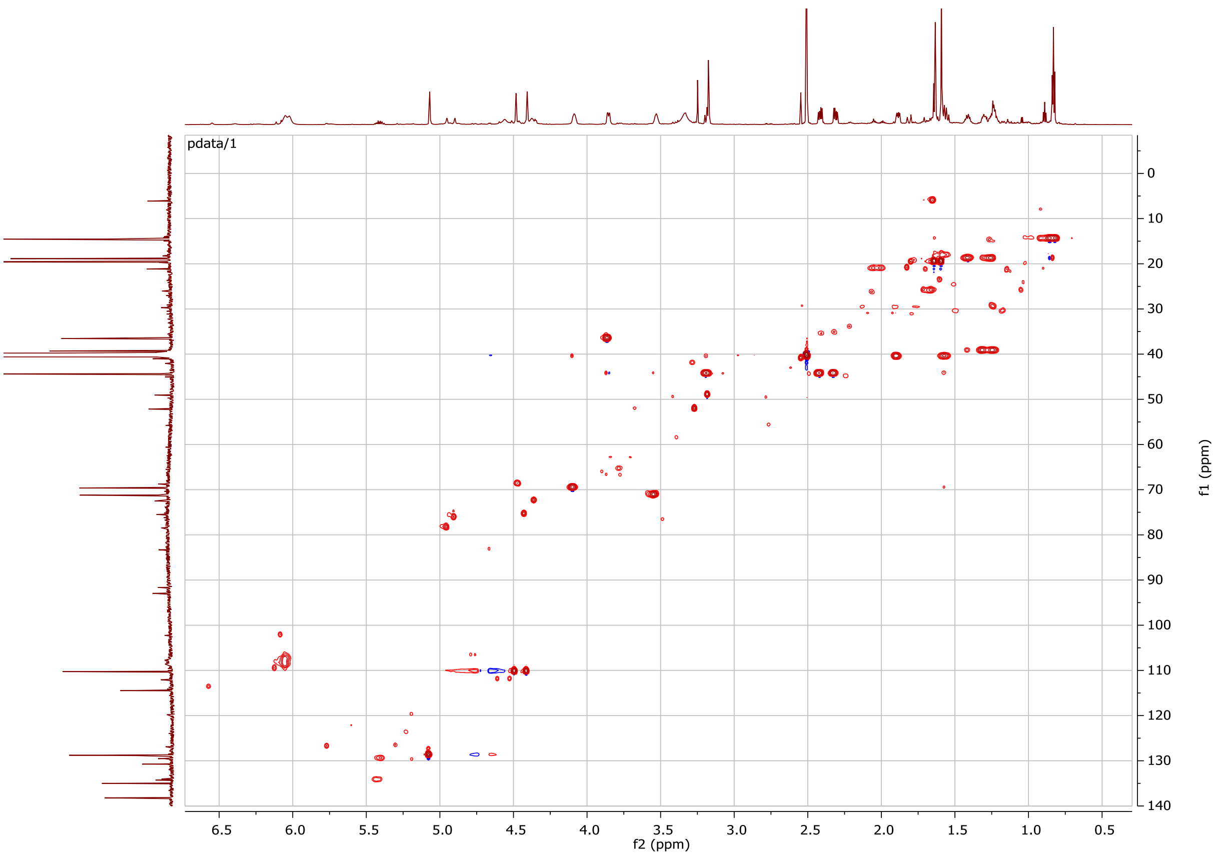

Figure S81. HSQC (1H-13C) NMR spectrum of metabolite **9**

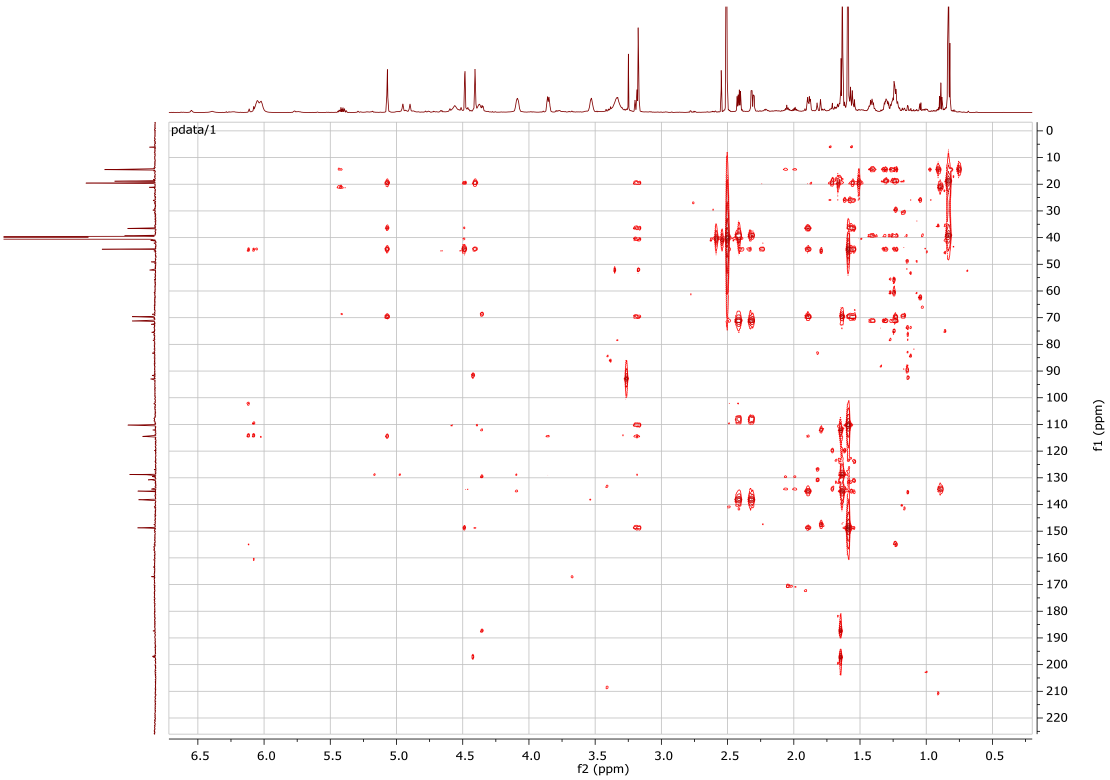

Figure S82. HMBC (long distance correlation ^{1}H-^{13}C) NMR spectrum of metabolite **9**

10. Statistical analysis

Table S1. One-way ANOVA – Time significance

One-way ANOVA

SUMMARY

Groups	*Count*	*Sum*	*Mean*	*Variance*
Day 1	3	12.05135	4.017115	0.106401
Day 3	3	9.621467	3.207156	0.296782
Day 5	3	8.871741	2.957247	0.029211
Day 7	3	8.02416	2.67472	0.584577

One-way ANOVA

Source of Variation	*SS*	*df*	*MS*	*F*	*P-value*	*F critical*
Between Groups	3.005357	3	1.001786	3.940271	0.05368	4.066181
Within Groups	2.033943	8	0.254243			
Total	5.039299	11				

Table S2. One-way ANOVA and Student's t-test– Metabolite **2** over time – *I. fumosorosea* KCh J2

One-way ANOVA

SUMMARY

Groups	Count	Sum	Mean	Variance
Day 1	3	8.916773	2.972258	0.154992
Day 3	3	8.743541	2.914514	0.027841
Day 5	3	6.885831	2.295277	0.003847
Day 7	3	5.308234	1.769411	0.042277

One-way ANOVA

Source of Variation	SS	df	MS	F	P-value	F critical
Between Groups	2.909794	3	0.969931	16.94518	0.000794	4.066181
Within Groups	0.457915	8	0.057239			
Total	3.367708	11				

Metabolite 2

Day 1	Day 7	T-student
3.411478	1.682688	
2.651127	2.004179	0.018109
2.854169	1.621366	

Table S3. One-way ANOVA and Student's t-test– Metabolite **8** over time - *I. fumosorosea* KCh J2

One-way ANOVA

SUMMARY

Groups	Count	Sum	Mean	Variance
Day 1	3	3.398848	1.132949	0.672694
Day 3	3	6.046763	2.015588	0.137246
Day 5	3	6.820451	2.273484	0.062326
Day 7	3	7.398666	2.466222	0.066547

One-way ANOVA

Source of Variation	SS	df	MS	F	P-value	F critical
Between Groups	3.123161	3	1.041054	4.435615	0.040871	4.066181
Within Groups	1.877627	8	0.234703			
Total	5.000788	11				

Metabolite 8

Day 1	Day 7	T-Student
2.079442	2.470639	
0.631272	2.206074	0.095257
0.688135	2.721953	

Table S4. One-way ANOVA and Student's t-test– Metabolite **7** over time - *I. fumosorosea* KCh J2

One-way ANOVA

SUMMARY

Groups	Count	Sum	Mean	Variance
Day 1	3	8.599751	2.866584	0.07391
Day 3	3	11.50696	3.835655	0.014395
Day 5	3	12.31786	4.105952	0.000467
Day 7	3	12.50768	4.169226	0.013407

One-way ANOVA

Source of Variation	SS	df	MS	F	P-value	F critical
Between Groups	3.270257	3	1.090086	42.67314	2.87E-05	4.066181
Within Groups	0.20436	8	0.025545			
Total	3.474617	11				

Metabolite 7		
Day 1	Day 7	T-student
3.160399	4.080753	
2.815409	4.12665	0.006657
2.623944	4.300274	

Table S5. Student's t-test– *M. hiemalis* strains

	KCh W2	AM 450	Ln KCh W2	Ln AM450	T-Student (p)
CBD	1.82	35.6	0.598836501	3.572345638	
	1.36	14.02	0.3074847	2.640484882	0.003124556
	0.6	15.8	-0.510825624	2.76000994	
Metabolite 2	69.76	25.99	4.24506078	3.257711849	
	64.14	29.08	4.161068194	3.370050653	0.002289354
	61.5	22.11	4.119037175	3.096029995	
Metabolite 3	21.66	1.39	3.075467242	0.329303747	
	23.59	0.74	3.160822893	-0.301105093	0.023931245
	27.27	0.18	3.305787197	-1.714798428	
Metabolite 4	2.07	31.24	0.727548607	3.441699325	
	0.31	49.01	-1.171182982	3.892024359	0.014433334
	0.38	53.07	-0.967584026	3.971611797	
Metabolite 5	3.06	2.97	1.118414916	1.088561953	
	4.53	4.16	1.510721939	1.425515074	0.665629202
	3.6	5.42	1.280933845	1.690095815	
Metabolite 6	1.63	2.82	0.488580015	1.036736885	
	6.08	2.99	1.805004696	1.095273387	0.606964905
	6.65	3.42	1.894616855	1.229640551	
